# Module-detection approaches for the integration of multilevel omics data highlight the comprehensive response of *Aspergillus fumigatus* to caspofungin

**DOI:** 10.1186/s12918-018-0620-8

**Published:** 2018-10-20

**Authors:** T. Conrad, O. Kniemeyer, S. G. Henkel, T. Krüger, D. J. Mattern, V. Valiante, R. Guthke, I. D. Jacobsen, A. A. Brakhage, S. Vlaic, J. Linde

**Affiliations:** 1 Systems Biology/Bioinformatics, Leibniz Institute for Natural Product Research and Infection Biology – Hans Knöll Institute, Jena, Germany; 2 Molecular and Applied Microbiology, Leibniz Institute for Natural Product Research and Infection Biology – Hans Knöll Institute, Jena, Germany; 3 BioControl Jena GmbH, Jena, Germany; 4 Biobricks of Microbial Natural Product Syntheses, Leibniz Institute for Natural Product Research and Infection Biology – Hans Knöll Institute, Jena, Germany; 5 Microbial Immunology, Leibniz Institute for Natural Product Research and Infection Biology – Hans Knöll Institute, Jena, Germany; 6 Institute for Microbiology, Friedrich Schiller University, Jena, Germany; 7 Research Group PiDOMICs, Leibniz Institute for Natural Product Research and Infection Biology – Hans Knöll Institute, Jena, Germany; 8 Institute for Bacterial Infections and Zoonoses, Federal Research Institute for Animal Health – Friedrich Loeffler Institute, Jena, Germany; 9 Present address: PerkinElmer Inc., Rodgau, Germany

**Keywords:** Multilevel, Omics, Protein-protein interaction network, Module, *Aspergillus fumigatus*, Caspofungin, Stress response, ModuleDiscoverer

## Abstract

**Background:**

Omics data provide deep insights into overall biological processes of organisms. However, integration of data from different molecular levels such as transcriptomics and proteomics, still remains challenging. Analyzing lists of differentially abundant molecules from diverse molecular levels often results in a small overlap mainly due to different regulatory mechanisms, temporal scales, and/or inherent properties of measurement methods. Module-detecting algorithms identifying sets of closely related proteins from protein-protein interaction networks (PPINs) are promising approaches for a better data integration.

**Results:**

Here, we made use of transcriptome, proteome and secretome data from the human pathogenic fungus *Aspergillus fumigatus* challenged with the antifungal drug caspofungin. Caspofungin targets the fungal cell wall which leads to a compensatory stress response. We analyzed the omics data using two different approaches: First, we applied a simple, classical approach by comparing lists of differentially expressed genes (DEGs), differentially synthesized proteins (DSyPs) and differentially secreted proteins (DSePs); second, we used a recently published module-detecting approach, ModuleDiscoverer, to identify regulatory modules from PPINs in conjunction with the experimental data. Our results demonstrate that regulatory modules show a notably higher overlap between the different molecular levels and time points than the classical approach. The additional structural information provided by regulatory modules allows for topological analyses. As a result, we detected a significant association of omics data with distinct biological processes such as regulation of kinase activity, transport mechanisms or amino acid metabolism. We also found a previously unreported increased production of the secondary metabolite fumagillin by *A. fumigatus* upon exposure to caspofungin. Furthermore, a topology-based analysis of potential key factors contributing to drug-caused side effects identified the highly conserved protein polyubiquitin as a central regulator. Interestingly, polyubiquitin UbiD neither belonged to the groups of DEGs, DSyPs nor DSePs but most likely strongly influenced their levels.

**Conclusion:**

Module-detecting approaches support the effective integration of multilevel omics data and provide a deep insight into complex biological relationships connecting these levels. They facilitate the identification of potential key players in the organism’s stress response which cannot be detected by commonly used approaches comparing lists of differentially abundant molecules.

**Electronic supplementary material:**

The online version of this article (10.1186/s12918-018-0620-8) contains supplementary material, which is available to authorized users.

## Background

The permanent growth in the development and improvement of new measurement techniques have led to a wealth of data from heterogeneous sources. The integration of all available data obtained from diverse studies has the potential to provide a more comprehensive and deeper understanding of the studied subject [1–3]. One example is the investigation of an organism’s response to an external stimulus at different molecular levels. Large-scale studies at molecular levels like transcriptomics, proteomics, lipidomics or metabolomics can be summarized by the term ‘omics levels’. These omics levels are linked to each other and are considered in their entirety. They describe the overall biological processes which occur in the analyzed organism. Potential links can be characterized by level-shared (‘overlapping’) components (such as genes or proteins) or the participation of components of different molecular levels in level-shared pathways.

As widely reported, the integration and analysis of data from multiple levels measured with diverse techniques at different time points are challenging. In an intuitive and commonly used approach (‘simple approach’), the analysis of several sets of omics data is based on the comparison of lists of differentially expressed genes (DEGs) and differentially synthesized proteins (DSyPs) identified in experimental datasets. However, the use of only DEGs and DSyPs is threshold-dependent and usually incomplete due to experimental limitations. For example, the use of liquid chromatography-mass spectrometry (LC-MS/MS)-based shotgun proteomics analysis for the identification of DSyPs is usually limited in the quantification of low abundant proteins due to the large dynamic range of protein abundances that needs to be covered [4, 5]. Other approaches, including diverse pathway enrichment analyses, assign both differentially and non-differentially expressed genes or their synthesized proteins to specific pathways which are part of biological processes. The level of activity of such pathways can be estimated by taking into account measurements of changes in gene expression or protein synthesis. However, as these approaches are based on pre-defined lists of pathways, they exclude unknown pathways which may also have important functions [6]. Over the last decades, the analysis of protein-protein interaction networks (PPINs) has become a useful approach [7]. By identifying direct (physical) contacts and indirect interactions (e.g., via regulatory cascades) between two or more proteins, PPINs point to structural and functional relationships between their nodes [8]. Several de novo network enrichment approaches were developed to extract connected sub-networks from larger interaction networks. Such sub-networks containing sets of closely related proteins are defined as modules [9]. There are many examples in the literature demonstrating the usefulness of modules in research data interpretation. For instance, Stuart et al. analyzed genetic modules to detect co-expressed genes that are involved in similar biological processes [10], while Trevino et al. [11] have shown the usefulness of investigating inter-module connectivity to identify molecular cross-talk between normal prostate epithelial and prostate carcinoma cells.

Another very interesting application of modules is the identification of prognostic or drug response biomarkers [12]. In this context, modules also show their potential for the characterization of drug-caused side effects occurring in addition to effects on the intended primary drug target. Wang et al. [13] demonstrated that major contributing factors of such side effects can be investigated by considering the primary drug target and its local network structure.

**Fig. 1:**
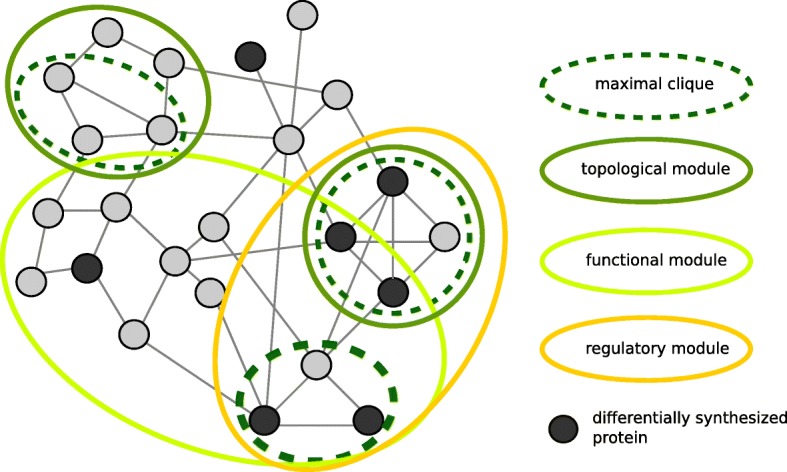
Module categories. Exemplarily selected categories of modules within protein-protein interaction networks. Proteins are represented by circles, interactions by edges

Several categories of modules have been described until now (Fig. 1). Examples are topological modules composed of proteins showing a high degree of inner-connectiveness or functional modules that contain proteins associated to specific biological functions [14, 15]. So-called regulatory modules are defined as sets of co-expressed genes which share a common function [16]. Popular methods for the detection of regulatory modules are: DEGAS [17], MATISSE [15], KeyPathwayMiner [18] and ModuleDiscoverer [19]. Among them, the recently published ModuleDiscoverer (MD) includes a heuristic that approximates the PPIN’s underlying community structure based on maximal cliques. While a community defines a group of proteins featuring a higher within-edge density in comparison to the edge density connecting them, a clique represents a set of proteins with edges between each pair of them. A clique is maximal if no node (e.g., protein) exists which extends that clique. MD was shown to be very efficient in the detection of regulatory modules for gene expression data in the context of animal models of non-alcoholic fatty liver disease [19].

In this study, we applied the simple approach (SA), the recently published module-detection approach MD as well as KeyPathwayMiner to experimental data of different molecular levels, measurement techniques and time points. As a case study, we analyzed the molecular response of the human pathogenic fungus *Aspergillus fumigatus* to the antifungal drug caspofungin. *A. fumigatus* causes local and systemic infections in immunocompromised individuals [20]. One therapeutic approach is the use of the lipopeptide caspofungin of the group of echinocandins. Caspofungin specifically targets the fungal cell wall by inhibiting the synthesis of the polysaccharide β-(1,3)-D-glucan [21]. Fungal cells respond to caspofungin by the adaption of gene expression and, consequently, protein biosynthesis and secretion of molecules [22]. Therefore, we analyzed the transcriptomic, proteomic and secretomic response of *A. fumigatus* to caspofungin at several time points to gain a deeper understanding of the overall molecular response of this fungus to this drug.

We demonstrated the so far untested capacity of MD to integrate multilevel omics data and showed that this level of integration is not achievable using SA. Moreover, module-detecting approaches facilitate the identification of potential key players in the organism’s stress response which are not detectable by commonly used approaches comparing lists of differentially abundant molecules.

## Methods

### Omics data and data processing

Data analyses were performed in R version 3.4.1 using packages provided by Bioconductor [23].

### Strain and culture conditions

Mycelia of the *Aspergillus fumigatus* strain CEA17 Δ*akuB* [24] were pre-cultured for 16 h in *Aspergillus* minimal medium (AMM, [25]) containing 50 mM glucose and 70 mM NaNO_3_ and then stressed with a sub-inhibitory concentration of caspofungin (100 ng/ml) as described in Altwasser et al. [26]. Liquid cultures were inoculated with 1 × 10^6^ conidia/ml and cultivated at 37 °C with shaking at 200 rpm. Samples for analyzing the transcriptomic, proteomic and secretomic response of the fungus were taken at the indicated time points after treatment. Secreted proteins were precipitated overnight from culture supernatants as described below.

### Transcriptome data

RNA extraction, cDNA library construction and RNA-Seq analysis by Illumina next-generation sequencing of samples taken at 0 h, 0.5 h, 1 h, 4 h and 8 h after caspofungin treatment were performed as described in [26]. Likewise, data were pre-processed as described in [26]. Genes were annotated by identifiers provided by the *Aspergillus* Genome Database (AspGD, as of September 2015 [27]). In addition, identifiers provided by the Central *Aspergillus* Data Repository (CADRE) [28] were obtained using the package *biomaRt* [29] provided by Bioconductor as of February 2017. For each time point, expression values were compared to the control sample taken at 0 h. Only those genes with an absolute log2 Fold Change (log2FC) value greater 1 and a False Discovery Rate (FDR) corrected *p*-value below 0.05 were considered to be differentially expressed.

### Proteome and secretome data

Samples for proteome analysis were taken at 0 h, 4 h and 8 h after treatment. The mycelium was collected by filtering through Miracloth (Merck Millipore), subsequently washed with water and snap frozen with liquid nitrogen. Sample preparation of the mycelium for the proteome analysis was performed as previously described [30]. Samples for secretome analysis were taken at 0 h and 8 h after treatment and prepared as follows: Cell free-filtered supernatant of AMM medium from *A. fumigatus* cultures was precipitated by trichloroacetic acid (TCA) at 15% (*w*/*v*) final concentration (4 °C, overnight). Precipitates were washed with acetone and resolubilized in trifluoroethanol (TFE) mixed 1:1 with 100 mM triethylammonium bicarbonate (TEAB). Samples containing 100 μg of total protein (in 100 μl) were reduced with 50 mM tris(2-carboxyethyl)phosphine (TCEP) for 1 h at 55 °C and subsequently cysteine thiols were alkylated with 12.5 mM iodoacetamide for 30 min at room temperature. Proteins were digested at 37 °C for 18 h with trypsin+LysC mix (Promega) at 1:25 protease:protein ratio. Proteome samples were labeled with tandem mass tags (TMT) 6plex and secretome samples were labeled with isobaric tags for relative and absolute quantification (iTRAQ) 4plex according to the manufacturer’s protocols.

LC-MS/MS analysis was performed as previously described [30] with the following modifications: Eluents A (0.1% *v*/v formic acid in H_2_O) and B (0.1% v/v formic acid in 90/10 ACN/H_2_O *v*/v) were mixed for 10 h gradient elution: 0–4 min at 4% B, 15 min at 5.5% B, 30 min at 6.5%, 220 min at 12.5% B, 300 min at 17% B, 400 min at 26% B, 450 min at 35% B, 475 min at 42% B, 490 min at 51% B, 500 min at 60% B, 515–529 min at 96% B, 530–600 min at 4% B. Precursor ions were monitored at m/z 300–1500, *R* = 140 k (FWHM), 3e6 AGC (automatic gain control) target, and 120 maximum injection time (maxIT). Top ten precursor ions (0.8 Da isolation width; z = 2–5) underwent data-dependent higher-energy collisional dissociation (HCD) fragmentation at normalized collision energy (NCE) 36% using N_2_ gas. Dynamic exclusion was set to 40 s. MS^2^ spectra were monitored at *R* = 17.5 k (FWHM), 2e5 AGC target, and 120 maxIT. The fixed first mass was set to m/z 110 to match the iTRAQ reporter ions (m/z 114–117).

Database searches were performed by Proteome Discoverer (PD) 1.4 (Thermo Fisher Scientific, Dreieich, Germany) using the AspGD protein database of *A. fumigatus* Af293 [31] and the algorithms of MASCOT 2.4.1 (Matrix Science, UK), SEQUEST HT (integral search engine of PD 1.4), and MS Amanda 1.0. Two missed cleavages were allowed for tryptic digestion. The precursor mass tolerance and the integration tolerance (most confident centroid) were set to 5 ppm and the MS2 tolerance to 0.02 Da. Static modifications were carbamidomethylation of cysteine and either TMT6plex (proteome) or iTRAQ4plex (secretome) at lysine residues and the peptide N-terminus. Dynamic modifications were oxidation of methionine and either TMT6plex of threonine or iTRAQ4plex of tyrosine. Percolator and a reverse decoy database were used for *q*-value validation of the spectral matches (Δcn < 0.05). At least two peptides per protein and a strict target FDR < 1% were required for confident protein hits. The significance threshold for differential protein abundances for TMT and iTRAQ experiments was set to factor 1.5.

With the aid of the *biomaRt* package, proteins were annotated using identifiers provided by AspGD as of September 2015 and CADRE as of February 2017.

### Chemical analysis of secondary metabolites

For quantification of fumagillin, fungal cultures were extracted and run on a LC-MS system consisting of an HPLC, UltiMate 3000 binary RSLC with photo diode array detector (Thermo Fisher Scientific, Dreieich, Germany) and the mass spectrometer (LTQ XL Linear Ion Trap from Thermo Fisher Scientific, Dreieich, Germany) with an electrospray ion source as described in Jöhnk et al. [32]. Data were obtained from three biological replicates and three technical replicates. A standard curve (1000, 500, 250, 125 and 62.5 μg/mL) using an authentic fumagillin standard (Abcam, United Kingdom) was calculated. The Xcalibur Quan Browser software (Thermo Fisher Scientific, Dreieich, Germany) was used to calculate the amounts of fumagillin.

### Application of module-detecting approaches

A high-confidence (score > 0.7) PPIN of *A. fumigatus* strain A1163 was downloaded from STRING version 10 [33]. Both the PPIN and the pre-processed omics data were taken as input for the module-detecting approaches. Thereby, protein identifier annotations provided by CADRE were used.

### ModuleDiscoverer

In order to apply MD for transcriptome data, the background contains all known *A. fumigatus* proteins described in AspGD. Analyzing proteome and secretome data, all proteins detected via LC-MS/MS were taken as background. The single-seed MD algorithm was applied to the input data as described by Vlaic et al. [19]. In brief, maximal cliques were identified using only one seed node in the PPIN. Cliques were tested for their enrichment with DEGs/DSyPs/DSePs using a permutation-based test as described in Vlaic et al. [19]. Cliques with a *p*-value < 0.01 were considered significantly enriched. Based on the union of these significantly enriched cliques, the regulatory module was assembled.

For the integration of different omics datasets, all regulatory modules were merged by forming the union of all nodes and edges. The resulting union regulatory module is defined as ‘overall regulatory module’ (ORM). Sub-modules with a number of nodes < 10 were not considered. Cytoscape version 3.2.1 [34] was used to visualize and analyze regulatory modules, for example, regarding their nodes’ degree and betweenness centrality.

### KeyPathwayMiner

KeyPathwayMiner (KPM) detects maximal connected sub-networks. In these sub-networks, all but a specific number *K* components are DEGs, DSyPs or DSePs in all but at most a specific number *L* cases [18]. In this study, cases are defined as the available time points. In a first analysis (I), KPM was applied to each single experimental dataset to receive one module for each time point of the respective molecular level. In the single-level analysis (II), the modules for each molecular level over all time points were identified. A third analysis (III) directly combined all of the experimental datasets to get the overall regulatory module. For the KPM input, one matrix for each time point (I) or molecular level ((II) and (III)) were generated consisting of information about the components’ regulation at the respective time points. For (II) and (III), only those components were considered that were DEGs/DSyPs/DSePs in at least one of the time points of the respective molecular level. With these matrices, the *A. fumigatus* PPIN and with the aid of KeyPathwayMiner Cytoscape App [18], sub-networks were computed using following settings: Ant colony optimization meta heuristic (ACO) as search algorithm, individual node exceptions (INEs) as search strategy, maximum of exception nodes *K* = 2. For (I) and (II), the maximal case exception parameter was set to *L* = 0. For the multilevel omics analysis (III), the logical connector of the different levels was set to the logical ‘OR’ and *L* was set to *L1* = 3 (transcriptome data),* L2* = 1 (proteome data) and *L3* = 0 (secretome data). These *L* values were based on the number of time points of the respective molecular level. The assumption was that the considered component is a DEG/DSyP/DSeP in at least one measured time point. For instance, as four measured transcriptome time points were available, a gene was allowed to be not differentially expressed in maximal three out of four time points. The top ten best-scoring sub-networks were selected for further analysis. A KPM regulatory module describes the union of these top ten sub-networks of the respectively considered datasets.

### Comparison of the simple approach and a module-detecting approach

#### Overlap of components

The overlap (percentage value) is defined as fraction of the intersection of the respective datasets from the union of the datasets. For the simple approach (SA), the overlap of different molecular levels was analyzed by comparing lists of DEGs, DSyPs and DSePs at the considered time points. For the module-detecting approach, the overlap of all components of the respective regulatory modules was considered.

In addition to the comparison of percentage values of overlapping components, a more objective measurement based on a permutation-based test was considered. Considering all known *A. fumigatus* proteins (*N*) described in AspGD, *D* ∈ *N* is a set of components (DEGs, DSyPs or DSePs) for each of the molecular levels. In *I* = 100,000 iterations, datasets *B* were created where each set consists of |*D*| components sampled from *N*. In every iteration, the overlap *P* of the molecular levels was calculated based on the generated datasets for transcriptome, proteome and secretome. The *p*-value was calculated by dividing the number of iterations in which *P* ≥ *O,* where *O* represents the overlap received by SA or MD, and the total number of iterations *I*.

#### Correlation of the components’ regulation

All components detected in at least one of the transcriptomic and one of the proteomic time points were considered for correlation analyses. The distance between results obtained for different molecular levels and time points was estimated based on the correlation of ranked lists of the components’ absolute gene expression or protein synthesis regulation values (absolute log2FCs). Lists of ordered, absolute regulation values were rank-transformed. Indices corresponding to ties (equal values) were randomly ordered. Spearman’s rank correlation coefficient *r* was calculated. The ranking was repeated 1000 times. Over all repeats, the final correlation between the regulation lists was averaged. The distance *d* is defined as *d =* 1 - *r*.

### Generalized topological overlap

The ORM was clustered via the generalized topological overlap measure (GTOM) as described in [35]. Matrix ${T}^{\left[m\right]}=\left[{t}_{ij}^{\left[m\right]}\right]$ is called the *m*-th order GTOM matrix and includes the overlap of nodes reachable from the nodes *i* and *j* within *m* steps:

$$ {t}_{ij}^{\left[m\right]}=\frac{\left|{N}_m(i)\cap {N}_m(j)\right|+{a}_{ij}+{I}_{i=j}}{\mathit{\min}\left\{\left|{N}_m(i)\right|,\left|{N}_m(j)\right|\right\}+1-{a}_{ij}} $$

*A* = [*a*_*ij*_] is defined as adjacency matrix, *N*_*m*_(*i*) as the set of neighbors of *i,* the Identity matrix *I*_*i* = *j*_equals 1 if *i = j* and zero else, |·| denotes the number of elements (cardinality) in its argument *j.* The clustering was performed for second-order connections. With the aid of the *hclust* function *(method = average),* a dendrogram based on all distances between proteins were generated. A cutoff of 0.65 was chosen to receive the clusters. R packages *RcolorBrewer* [36] *a*nd *WGCNA* [37] were applied for coloring the single clusters.

### Enrichment analysis (functional annotation of biological processes)

Gene Ontology (GO) terms were applied for functional annotation concerning biological processes. Gene (gene product) terms of *A. fumigatus* were retrieved from AspGD as of October 2017. In particular, GO information about the Af293 strain was extracted and imported into R and was transformed into custom annotation objects by packages *AnnotationDbi* [38] and *GSEABase* [39] (each of version 1.38.2 as part of Bioconductor package collection version 3.5). In addition, the packages *GO.db* [40], *GOstats* [41] as well as the helper function *GSEAGOHyperGParams* of package *Category* [42] were applied for the enrichment analysis. For SA, all *A. fumigatus* proteins described in AspGD were taken as background. For the MD approach, all proteins which are part of the PPIN downloaded from STRING, were taken as background. GO terms composed of at least two members, associated with at least two components and leading to *p*-values below 0.05 were considered as significantly enriched.

## Results

### Data overview

**Fig. 2:**
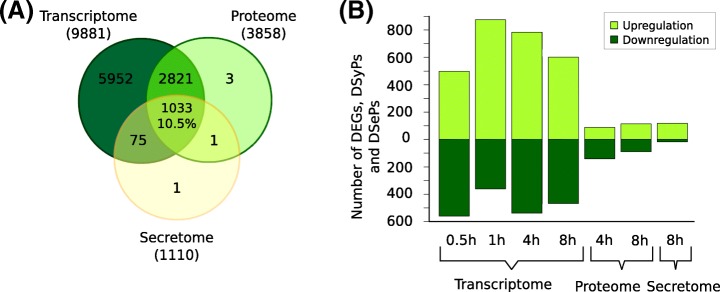
Overview of the available datasets. **a** Number and overlap of all measured genes and proteins. **b** Number of differentially expressed genes (DEGs), differentially synthesized proteins (DSyPs) and differentially secreted proteins (DSePs) in all available experimental datasets

We used experimental omics data of a *A. fumigatus* study that investigated the stress response to the antifungal drug caspofungin at different molecular levels (transcriptome, proteome, secretome) including different time points. Figure 2a provides an overview of the available datasets including all genes and proteins detected by RNA-Seq and LC-MS/MS. Over all considered time points, 9881 genes were measured for the transcriptomic response, 3858 proteins for the proteomic response and 1110 proteins for the secretome. Filtering the data for DEGs, DSyPs and DSePs resulted in 1058 DEGs (498 upregulated (↑), 560 downregulated (↓)) at 0.5 h, 1237 DEGs (876 ↑, 361 ↓) at 1 h, 1322 DEGs (784 ↑, 538 ↓) at 4 h and 1068 DEGs (600 ↑, 468 ↓) at 8 h after caspofungin treatment. In the proteome, 230 DSyPs (88 ↑, 142 ↓) were identified at 4 h after treatment, and 204 DSyPs (114 ↑, 90 ↓) at the 8 h time point. 136 DSePs (118 ↑, 18 ↓) were detected for the secretome at 8 h after treatment (Fig. 2b). Complete lists of DEGs, DSyPs and DSePs are provided in the Additional file 1.

### Overlap of datasets of the different molecular levels

**Fig. 3:**
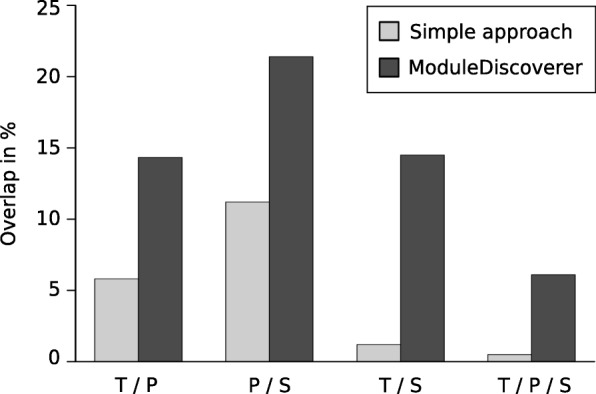
Overlap of molecular levels. Overlap of transcriptome (T), proteome (P) and secretome (S) regarding their components (genes or proteins)

We started to analyze the molecular level overlap by comparing all measured genes or proteins (hereafter called ‘components’) independently of their differential regulation and time points. This comparison showed that the overlap of all three molecular levels amounted to 10.5% (Fig. 2a). Applying SA and MD to the experimental data (Fig. 3), this level overlap accounted for 0.5% (SA) and 6.1% (MD). Considering only two out of three molecular levels (including data of all considered time points, respectively), both approaches resulted in the highest overlap for the proteome/secretome comparison (11.2% SA, 21.4% MD). This observation was not surprising as the secreted proteins are also included in the global proteome. We found that MD provided an up to 12-fold higher overlap than SA.

A further analysis of overlapping components considered a more objective measurement based on a permutation-based test. In 100,000 iterations, random datasets for transcriptome, proteome and secretome were generated and the overlap of all three datasets was calculated. The median-value of all 100,000 random overlaps equaled 3. Thus, the level overlap accounted for 0.1%. For the SA-obtained overlap (11 components or 0.5% as presented in Fig. 3), we calculated a *p*-value = 2.8 × 10^− 4^ which is statistically significant in comparison to random overlaps. In contrast, the MD-received overlap (58 components or 6.1% as presented in Fig. 3) resulted in the smaller *p*-value = 1.0 × 10^− 5^. Comparing the overlap percentage values, SA produced 5-fold and MD even 61-fold higher overlap values than random overlaps. The comparison of the SA- and MD-received overlap values resulted in the above-mentioned 12-fold higher values for MD.

### Estimation of the best match of transcriptomic and proteomic time points

The selection of measured time points was based on the following assumption: The expression of a gene and the synthesis of its corresponding protein do not occur at the same time since they are consecutive processes. Thus, changes in the transcriptional regulation are also reflected in the differential synthesis of proteins at the proteomic level but most likely at a later time point. Therefore, different time points at the transcriptomic and proteomic level were selected to consider the delay between transcription and translation during the fungal response. Hence, we analyzed our results regarding best matches of level- and time point-dependent sub-responses.

We tested two approaches for estimating the best transcriptome-proteome time point match: Comparison of components, and correlation of the components’ regulation. The first estimation approach aimed at analyzing overlapping components in the transcriptome and proteome which can be observed, for instance, as transcripts and their synthesized proteins. For the second estimation approach, the correlation of the components’ regulation was calculated based on absolute gene expression or protein synthesis regulation values. This approach represents the regulation of response pathways which not necessarily contain overlapping components but also other genes or proteins contributing to these pathways. Therefore, in this approach not only the overlapping components were analyzed but also components which are part from at least one of the respectively compared transcriptome and proteome time points. This leads to a higher number of considered components.

**Fig. 4:**
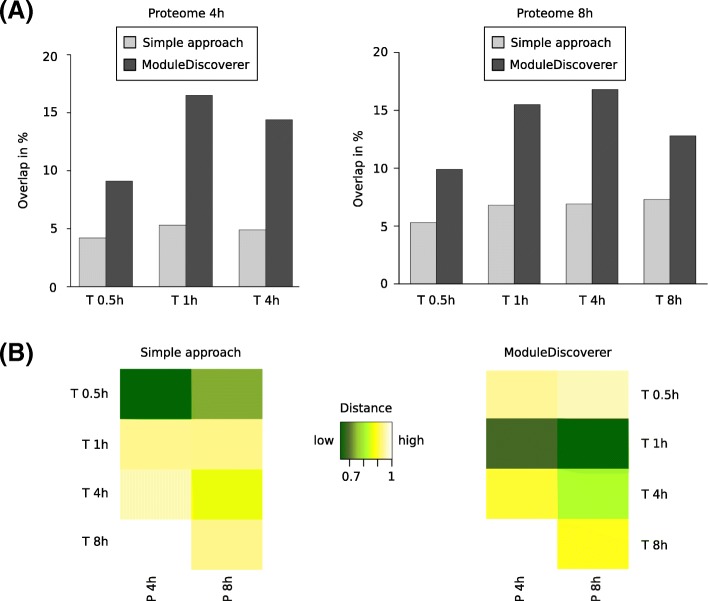
Transcriptome-proteome time point match. Estimation of the best time point match for transcriptome (T) and proteome (P) time points regarding **a** comparison of components and **b** correlation of the components’ regulation. Distance is defined as 1 minus correlation coefficient

Starting with the comparison of components (Fig. 4a), both SA and MD demonstrated the best match for the transcriptomic response 1 h and the proteomic response 4 h after caspofungin treatment (5.3% SA, 16.5% MD). While SA resulted in the best match of transcriptome at 8 h with proteome at 8 h (7.3%), MD showed the best match with transcriptome at 4 h (16.8%). Consequently, for both time point comparisons, MD-produced results indicated a delay of 3–4 h between the different sub-responses. Taking into account also the correlation of components’ regulation, Fig. 4b shows that similar to the previous analyses MD provided a better, i.e., here lower, distance for MD values than for SA. Oppositely to SA, the MD results confirmed the best time point match of transcriptome at earlier time point (1 h) and proteome at the later one (8 h) (Fig. 4b), similarly to the aforementioned comparison of components (Fig. 4a). The lowest distances were observed for the proteome at 8 h and transcriptome at 1 h (Fig. 4b, highlighted in dark green), followed by the proteome at 4 h and transcriptome at 1 h (Fig. 4b, dark green). These findings were also in agreement with the highest and second highest overlap values in Fig. 4a. Together with the observation that both approaches showed very high distance values (yellow and light yellow) between the same transcriptome and proteome time points, our results support the assumption of a time delay between level-dependent sub-responses (transcription and translation). Tendencies in the coherence of time points and an estimation of the resulting time delay between molecular levels may be helpful for further wet-lab studies regarding time- and cost-saving by focusing on the most relevant time points.

Another observation can be made by comparing the respective results of the two estimation approaches: There is a tendency that the correlation-based approach resulted in best matches for earlier transcriptome time points than the overlap-based approach. This observation may be based on the activation of stress response pathways induced by the fungus shortly after the caspofungin treatment. As such response pathways could involve components from both molecular levels transcriptome and proteome, we assume that the actual regulation of response pathways represented by the correlation-based approach already starts before the main translation process of potentially involved components occurs (represented by the overlap-based approach).

### Integration of multilevel omics data

#### Analysis of the overall fungal response to caspofungin

**Fig. 5:**
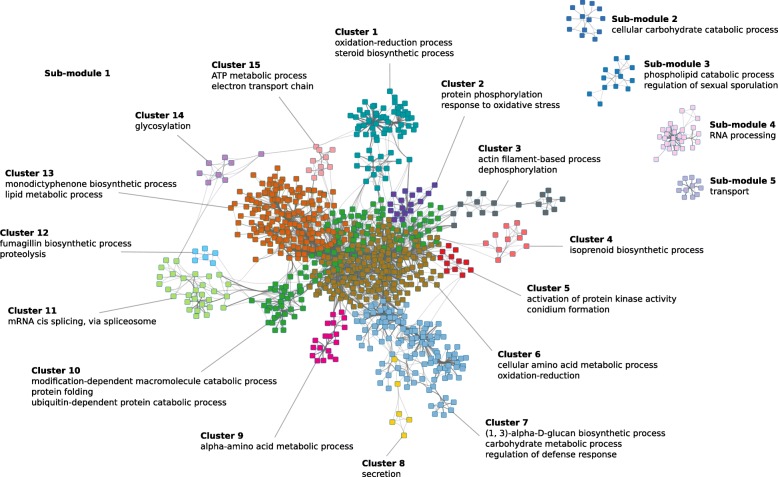
Overall regulatory module representing the response of *A. fumigatus* to caspofungin. The overall regulatory module identified by ModuleDiscoverer is composed of five sub-modules including 894 components (see Additional file 2: Table S2). Clusters with exemplarily selected significantly enriched biological processes are color-coded

All regulatory modules of each molecular level and time point identified by MD (Table 1 and Additional file 2: Table S1) can be considered to be part of the overall fungal response to caspofungin. Forming the union of them, the resulting overall regulatory module (ORM) was composed of five sub-modules including 894 components (Fig. 5). For a focused enrichment analysis based on the ORM’s underlying topology, we performed a generalized topological overlap measurement regarding the main sub-module 1. Figure 5 represents the ORM with its five sub-modules and the 15 clusters of sub-module 1, where the cluster membership of each protein is color-coded. An overview of all components of the ORM including sub-modules and clusters is available in Additional file 2: Table S2). GO term enrichment analyses showed that the clusters were significantly enriched with distinct biological functions (see Additional file 2: Tables S3–21) for a list of all significantly associated biological processes of each cluster and the remaining sub-modules). Examples of such processes are protein phosphorylation and response to oxidative stress (cluster 2, Additional file 2: Table S4), actin filament-based process (cluster 3, Additional file 2: Table S5), regulation of kinase activity (cluster 5, Additional file 2: Table S7), amino acid metabolic processes (cluster 6, Additional file 2: Table S8 and cluster 9, Additional file 2: Table S11), (1,3)-alpha-D-glucan biosynthesis (cluster 7, Additional file 2: Table S9), secondary and lipid metabolic process (cluster 12, Additional file 2: Table S14 and cluster 13, Additional file 2: Table S15) or transport mechanisms (cluster 15, Additional file 2: Table S17 and sub-module 5, Additional file 2: Table S21).

**Table 1:** Regulatory modules generated by ModuleDiscoverer

Underlying experimental dataset	Number of nodes (components)	Number of edges (interactions)
Transcriptome 0.5 h	511	2967
Transcriptome 1 h	256	1336
Transcriptome 4 h	313	1604
Transcriptome 8 h	256	1208
Proteome 4 h	147	845
Proteome 8 h	124	520
Secretome 8 h	293	2413
Overall regulatory module	894	6111

Number of nodes (representing gene or protein components) and edges (representing interactions between the components) of the regulatory modules received by ModuleDiscoverer

### Polyubiquitin and CBF/NF-Y family transcription factor as potential key factors contributing to the caspofungin-induced response

To investigate potential key factors in the fungal response contributing to, e.g., caspofungin-caused side effects, we analyzed the underlying topological network structure of the ORM. We took into account the network node-associated degree (number of edges connected to the node) and betweenness centrality (number of shortest paths that go through each node) [13]. We identified the node representing polyubiquitin UbiD with the fifth highest degree (Table 2) and the third highest betweenness centrality (Table 3). It was furthermore the only node that could be found in the top ten lists of both measures. Ubiquitin is a highly conserved 76-residue protein which can be found in all eukaryotic organisms [43]. In *Saccharomyces cerevisiae,* the orthologous gene UBI4, one out of four ubiquitin genes in yeast, was shown to be essential for resistance to different stresses including high temperatures and starvation [44].

**Table 2:** Nodes of the overall regulatory module with highest degree

CADRE-IDs	AspGD-IDs	Protein names	Degree	BC	log2FC						
					T 0.5 h	T 1 h	T 4 h	T 8 h	P 4 h	P 8 h	S 8 h
CADAFUBP00004294	AFUB_043760	Fatty acid synthase beta subunit, putative	146	0.126	−1.159	−0.628	− 0.693	− 0.828	− 0.006	− 0.136	0.534
CADAFUBP00004295	AFUB_043770	Fatty acid synthase alpha subunit FasA, putative	142	0.082	−1.008	−0.517	− 0.678	− 0.726	−0.038	− 0.105	1.448
CADAFUBP00002402	AFUB_024590	Acetyl-CoA carboxylase	124	0.122	−1.697	−0.812	−1.285	− 1.380	− 0.456	−0.628	1.518
CADAFUBP00004404	AFUB_044900	Nonribosomal peptide synthase SidE	114	0.048	1.077	1.918	1.613	1.229	NA	NA	NA
CADAFUBP00006564	AFUB_067450	Polyubiquitin UbiD/Ubi4, putative	111	0.396	−0.762	0.238	−0.248	−0.688	NA	NA	NA
CADAFUBP00007473	AFUB_076690	ATP citrate lyase, subunit 1, putative	98	0.037	−1.636	−0.705	−0.698	− 0.584	−0.474	− 0.521	0.683
CADAFUBP00007537	AFUB_077330	Bifunctional pyrimidine biosynthesis protein (PyrABCN), putative	82	0.073	−0.438	−0.468	− 0.214	−0.974	− 0.206	−0.340	1.586
CADAFUBP00000761	AFUB_007730	Glutamate synthase Glt1, putative	74	0.035	−2.168	−0.525	−0.119	− 0.483	−0.255	− 0.234	1.135
CADAFUBP00001006	AFUB_010250	Succinyl-CoA synthetase, alpha subunit, putative	72	0.021	−0.497	−0.273	0.167	−0.011	0.234	0.069	NA
CADAFUBP00003062	AFUB_031240	Sulfite reductase, putative	68	0.047	−0.900	−0.774	− 0.103	−0.598	− 0.016	−0.022	1.5

Top ten nodes of the overall regulatory module showing the highest degree and additional information regarding their betweenness centrality (BC) and gene- or protein-associated log2 Fold Change (log2FC) measured for the transcriptomic (T), proteomic (P) and secretomic (S) fungal response to caspofungin at all time points, respectively

**Table 3:** Nodes of the overall regulatory module with highest betweenness centrality

CADRE-IDs	AspGD-IDs	Protein names	Degree	BC	log2FC						
					T 0.5 h	T 1 h	T 4 h	T 8 h	P 4 h	P 8 h	S 8 h
CADAFUBP00007914	AFUB_081260	Peptidyl-arginine deiminase domain protein	4	0.6	0.696	3.125	2.328	1.647	NA	NA	NA
CADAFUBP00001626	AFUB_016580	Long-chain-fatty-acid-CoA ligase, putative	11	0.405	−1.395	−1.605	−0.406	−0.750	NA	NA	NA
CADAFUBP00006564	AFUB_067450	Polyubiquitin UbiD/Ubi4, putative	111	0.396	−0.762	0.238	−0.248	−0.688	NA	NA	NA
CADAFUBP00008739	AFUB_089890	Mandelate racemase/muconate lactonizing enzyme family protein	10	0.304	1.791	0.546	0.247	0.055	NA	NA	NA
CADAFUBP00003378	AFUB_034540	Lysophospholipase 3	5	0.303	0.906	1.357	1.185	1.357	0.706	0.937	1.513
CADAFUBP00002707	AFUB_027690	Lysophospholipase	10	0.273	−1.454	−0.217	−0.854	−1.700	0.387	0.192	NA
CADAFUBP00006379	AFUB_065540	Patatin-like phospholipase domain-containing protein	10	0.273	−0.639	− 0.570	−0.931	−1.460	NA	NA	NA
CADAFUBP00008747	AFUB_089980	Ribosome biogenesis protein (Rrs1), putative	26	0.259	1.045	−0.096	−0.154	0.413	0.185	0.045	NA
CADAFUBP00004062	AFUB_041460	Plasma membrane ATPase	4	0.25	−1.535	−0.670	−1.002	−1.152	0.041	−0.023	1.910
CADAFUBP00005096	AFUB_052070	Plasma membrane ATPase	4	0.25	−0.378	− 0.299	0.441	0.463	NA	NA	NA
CADAFUBP00000491	AFUB_004970	Alcohol dehydrogenase, zinc-containing, putative	3	0.167	−1.712	−1.178	−1.125	−0.941	−0.759	−0.454	0.914

Top ten nodes of the overall regulatory module showing the highest betweenness centrality (BC) and additional information regarding their node degree and gene- or protein-associated log2 Fold Change (log2FC) measured for the transcriptomic (T), proteomic (P) and secretomic (S) fungal response to caspofungin at all time points, respectively

In addition to this topology-based approach, we also applied an approach focused on transcription factors. Transcription factors play an important role in regulating the compensatory stress response to drugs. However, in many cases, it is difficult to measure transcription factors’ activity since they are often constitutively expressed and/or activated posttranscriptionally. Therefore, we scanned the ORM for transcription factors connected to DEG-associated proteins, DSyPs or DSePs (Table 4). Among them, we detected the CBF/NF-Y family transcription factor. It shows similarities to DNA polymerase epsilon subunit *DPB4* of *S. cerevisiae* and *Schizosaccharomyces pombe*.

**Table 4:** Transcription factors within the overall regulatory module

CADRE-IDs	AspGD-IDs	Protein names	log2FC						
			T 0.5 h	T 1 h	T 4 h	T 8 h	P 4 h	P 8 h	S 8 h
CADAFUBP00000978	AFUB_009970	CBF/NF-Y family transcription factor, putative	0.230	0.447	−0.124	0.002	−0.034	−0.150	NA
CADAFUBP00001789	AFUB_018340	HLH transcription factor, putative	−1.441	−0.236	0.128	−0.355	NA	NA	NA
CADAFUBP00003751	AFUB_038290	Zinc knuckle transcription factor/splicing factor MSL5/ZFM1, putative	0.366	0.062	0.111	0.143	−0.368	−0.322	3.379
CADAFUBP00004232	AFUB_043140	Transcription elongation factor SPT6, putative	−0.369	−0.235	− 0.177	−0.563	0.143	0.188	NA
CADAFUBP00005084	AFUB_051950	PHD transcription factor (Rum1), putative	−1.069	0.036	0.089	−0.780	−0.071	− 0.042	NA
CADAFUBP00007653	AFUB_078520	Stress response regulator/HFS transcription factor, putative	0.317	−0.271	0.034	−0.102	−0.130	0.072	NA
CADAFUBP00001318	AFUB_013400	TFIIH complex helicase Rad3, putative	0.154	0.034	−0.055	− 0.226	−0.032	0.222	NA
CADAFUBP00003811	AFUB_038920	Ccr4-Not transcription complex subunit (NOT1), putative	−0.786	−0.076	0.038	−0.834	− 0.100	−0.041	NA

Transcription factors detected in the overall regulatory module and their log2 Fold Change (log2FC) measured for the transcriptomic (T), proteomic (P) and secretomic (S) fungal response to caspofungin at all time points, respectively

**Fig. 6:**
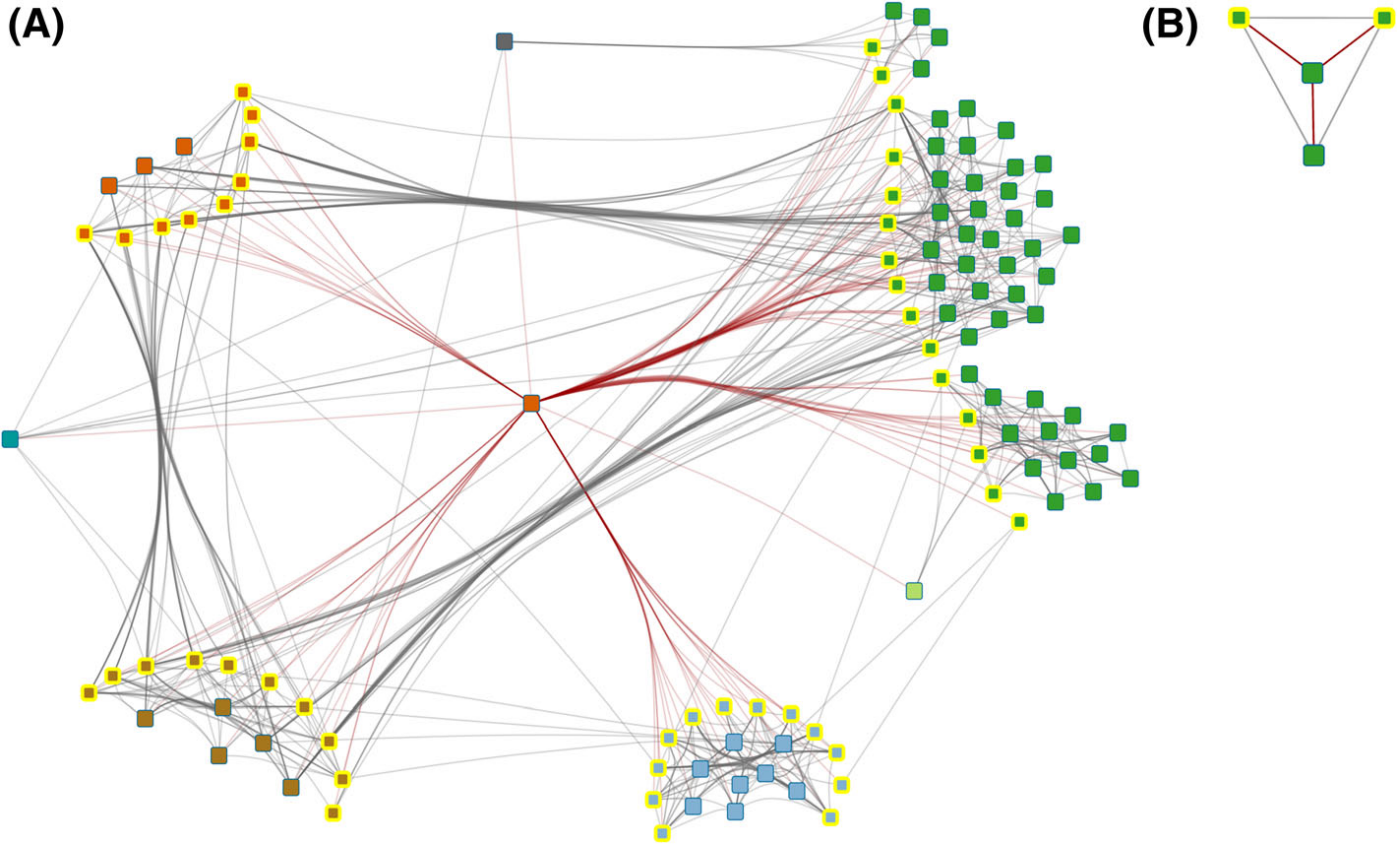
Potential key factors within the overall regulatory module contributing to the caspofungin-caused fungal response. **a** Polyubiquitin and **b** CBF/NF-Y family transcription factor (centrally arranged, respectively) and their first neighbors in the overall regulatory module. DEG-associated proteins, DSyPs and DSePs are highlighted with a yellow border

Both polyubiquitin and the CBF/NF-Y family transcription factor were detected in all transcriptome and, in case of the CBF/NF-Y family transcription factor, proteome time points but neither as DEG nor as DSyP. Figure 6 represents these two nodes and their respective first neighbors (including DEGs, DSyPs or DSePs) within the ORM.

The investigation of potential key factors in the drug-induced response, like polyubiquitin and CBF/NF-Y family transcription factor, may help to better understand the position and dynamics of drug targets and associated proteins in the interaction network and can potentially contribute to increase the safety of drugs.

### Caspofungin induces increased production of the secondary metabolite fumagillin

**Fig. 7:**
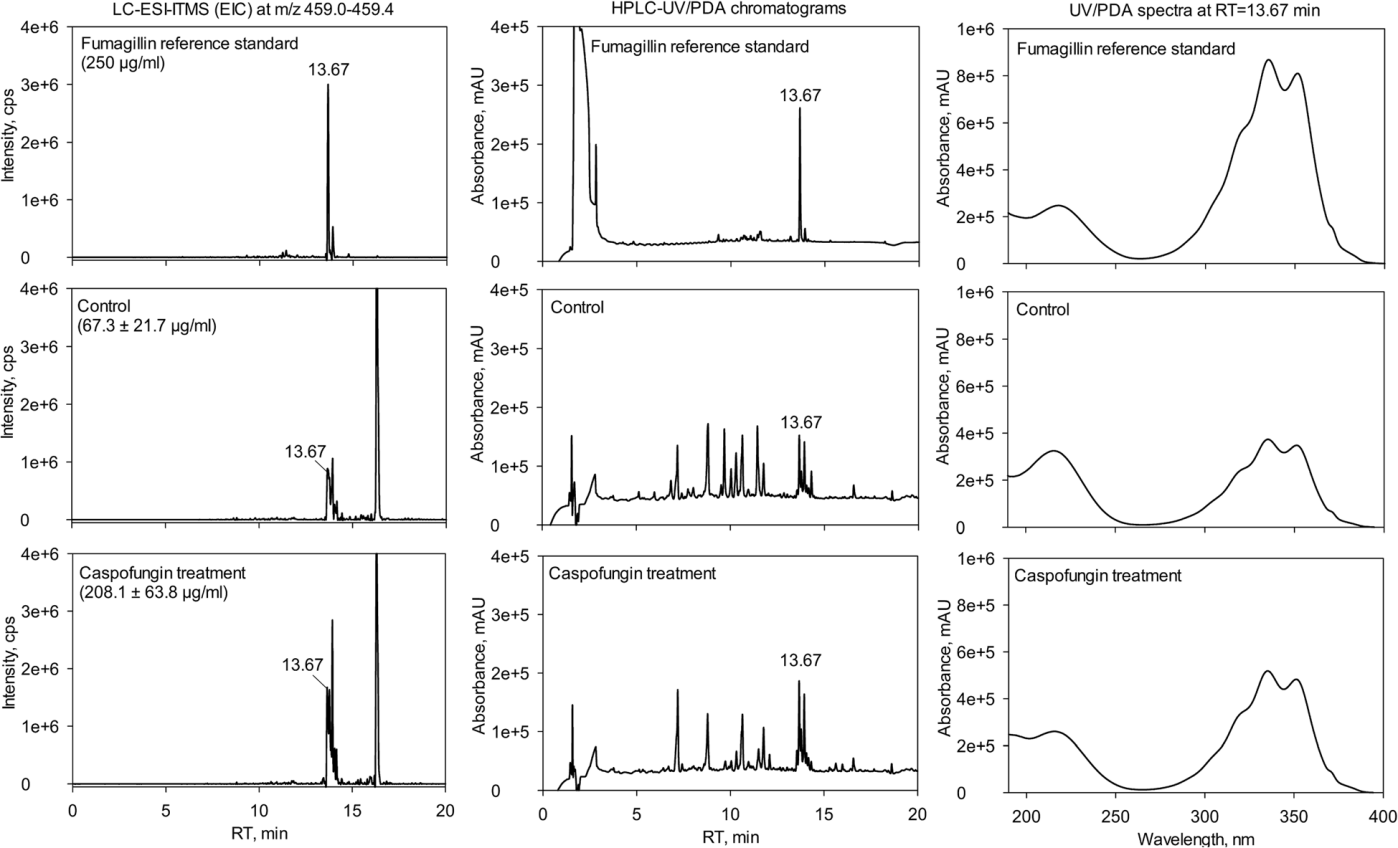
Caspofungin-induced increased production of the secondary metabolite fumagillin. LC-ESI-ITMS extracted ion chromatograms (EIC) at m/z 459.0–459.4 amu (left), HPLC-UV/PDA chromatograms (center) and UV/PDA spectra at RT = 13.67 min (right) of 250 μg/ml fumagillin reference standard (top) and crude extract of *A. fumigatus* without (center) and with caspofungin treatment (bottom)

As described above, the ORM contained two clusters, cluster 12 and 13, which included several enzymes that are involved in the biosynthesis of secondary metabolites. In particular, transcripts and their corresponding proteins of the antimicrobial agent fumagillin biosynthesis gene cluster (11 out of 15 cluster genes) showed increased levels after exposure of *A. fumigatus* to caspofungin. To verify whether caspofungin triggers the production of this meroterpenoid, we extracted *A. fumigatus* cultures exposed for 8 h to caspofungin (100 ng/ml) and control cultures with ethyl acetate and determined the fumagillin concentration by LC-MS. In cultures without caspofungin the concentration of fumagillin was 67.3 ± 21.7 μg/ml, while in cultures with caspofungin the concentration increased by 3-fold to 208.1 ± 63.8 μg/ml (Fig. 7). The level of other secondary metabolites such as pseurotin A stayed almost unchanged (Additional file 3).

### Comparison of ModuleDiscoverer- and KeyPathwayMiner-generated regulatory modules

To estimate the comprehensiveness of MD-generated regulatory modules, we applied another available module-detecting approach, KeyPathwayMiner (KPM), to our experimental datasets and compared the identified regulatory modules with those identified by MD (Table 5).

**Table 5:** Comparison of ModuleDiscoverer- and KeyPathwayMiner-detected regulatory modules

Underlying experimental dataset	Component number of MD modules	Overlap (percentage value regarding KPM module)	Component number of KPM modules
Transcriptome 0.5 h	511	134 (75.7%)	177
Transcriptome 1 h	256	62 (63.9%)	97
Transcriptome 4 h	313	123 (74.1%)	166
Transcriptome 8 h	256	89 (65.0%)	137
Proteome 4 h	147	36 (75.0%)	48
Proteome 8 h	124	30 (63.8%)	47
Secretome 8 h	293	42 (93.3%)	45
Overall regulatory module	894	343 (59.6%)	576

Comparison of ModuleDiscoverer (MD) and KeyPathwayMiner (KPM) regarding their number of module components. The overlap is defined as fraction of the intersection of the respective datasets from the KPM datasets

Table 5 shows the numbers of components of the KPM-produced regulatory modules for each time point and the overall regulatory module in comparison with those based on MD. Exemplarily, the comparison showed that the ORM received by MD contains a 1.5-fold higher number of components by covering more than 60% of KPM module components. Considering the modules of the single time point datasets, e.g. secretome at 8 h, we found an up to 6.5-fold higher component number by covering up to 93% of KPM components. Hence, we focused on the results received by MD. Nevertheless, additional KPM analyses regarding the overlap of molecular levels and the estimation of the best match of transcriptomic and proteomic time points are shown in Additional file 2: Figures S1 and S2 and Additional file 4.

## Discussion

In this study, we focused on the integration of omics data derived from heterogeneous sources. Therefore, we used experimental data of an *A. fumigatus* study investigating the stress response to the antifungal drug caspofungin at different molecular levels and time points. For the analyses, we applied SA considering only DEGs/DSyPs/DSePs and the regulatory module-detecting single-seed MD approach considering DEGs/DSyPs/DSePs, non-DEGs/DSyPs/DSePs as well as structural PPIN information. We focused on the single-seed approach instead of the also available multi-seed MD approach since the single-seed approach is comparable with other well-established maximal clique enumeration problem-based algorithms (e.g., Barrenäs et al. [45] or Gustafsson et al. [46]). In addition, Vlaic et al. showed that the multi-seed-identified modules can be essentially considered as an extension of the single-seed modules. However, we also applied the multi-seed approach to our experimental data set. In summary, the multi-seed MD approach allows for effectively integrating multilevel omics data. Multi-seed-generated results contain the regulatory modules received by the single-seed approach and are even more comprehensive. The overall regulatory module generated by the multi-seed approach confirms the already observed key players and significantly associated processes. Details on the analyses can be found in the Additional files 2 and 5.

### Relation of transcriptomic, proteomic and secretomic data

The comparison of all three molecular levels regarding all measured, SA- or MD-considered components resulted in only small overlap values. This observation is in agreement with other integrative transcriptomic and proteomic studies reporting that there is no or only a weak correlation between different molecular levels [47–49]. Potential explanations are biological (e.g., translational regulation or differences in protein and mRNA half-lives in vivo*)* or methodological origins (e.g., detection limits of the techniques or the choice of measured time points) [48, 49]. Figures 2a and 3 show an apparently contradictory outcome regarding the overlap of datasets of different molecular levels: Fig. 2a shows the highest overlap percentage value for transcriptome and proteome, Fig. 3 for proteome and secretome. This can be explained by the fact that Figs. 2a and 3 are based on analyses that considered diverse datasets. For Fig. 2a, all detected genes and proteins were analyzed. In contrast, Fig. 3 comprises only a fraction of these components because of a further filtering step to only compare DEGs/DSyPs/DSePs (SA) or regulatory module components (DEGs/DSyPs/DSePs and associated background proteins, MD). Actually, in Fig. 3, both approaches MD and SA showed the highest overlap between proteome and secretome. On the one hand, this highest overlap percentage reflects the same underlying measurement technique. In this study, the transcriptome was measured by RNA-Seq, the proteome and secretome by LC-MS/MS. As the techniques themselves are very different, also differences in their respective outcome can be expected. Therefore, as the intracellular proteome and secretome are based on the same measurement technique, they are more similar to each other than, for instance, transcriptome and proteome. On the other hand, the highest overlap also demonstrates the biological similarity in terms of immediately consecutive protein-based levels. Thus, both levels consist of proteins which differ only in the secretion step via classical (i.e., N-terminal secretory signal peptide triggered) or non-classical (i.e., without involvement of N-terminal signal peptides) secretory pathways [50]. Hence, proteome and secretome can be considered as immediately consecutive levels which can both be measured by LC-MS/MS.

By a general comparison of MD- and SA-received results, we determined up to 12-fold higher overlap values provided by MD than those calculated by SA. This is reasonable as SA focuses on the comparison of lists of DEGs, DSyPs and DSePs, exclusively. Hence, non-DEGs/DSyPs/DSePs measured in the experimental background were not considered which results in a high loss of data for the analyses. In contrast, the additional information considered by MD led to a much higher number of (overlapping) components.

### Analysis of the overall fungal response and potential key factors

With the aid of the ORM, we analyzed the *A. fumigatus* response to caspofungin over all molecular levels and time points. We found that ORM clusters are significantly enriched with biological functions like (1,3)-alpha-D-glucan biosynthesis and carbohydrate metabolic processes, actin filament-based processes, activation of protein kinase activity and response to oxidative stress. These results are in agreement with a genome-wide expression profiling study of *Aspergillus niger* in response to caspofungin [51]. Here, many of the upregulated genes were predicted or confirmed to function in cell wall assembly and remodeling, cytoskeletal organization, signaling and oxidative stress response. Also, genes and proteins of the electron transport chain were specifically enriched which supports the hypothesis that caspofungin acts as an effector of mitochondrial oxidative phosphorylation [52]. This is consistent with results from Cagas et al. [47] who analyzed the proteomic response of *A. fumigatus* to caspofungin and identified the largest change in a mitochondrial protein that has a role in mitochondrial respiratory chain complex IV assembly. The significant enrichment of genes and proteins of the amino acid metabolic process is best explained by the growth inhibitory activity of caspofungin that leads to the downregulation of the primary metabolisms including amino acid biosynthesis [53].

The cluster 5 represents (gene-associated) proteins involved in the activation of protein kinase activity. Mitogen-activated kinases (MAPK) are important regulators in the fungal response to stress that is induced by environmental changes or the disruption of cell wall integrity ([54], and references therein) which are both consequences of the caspofungin treatment. Also cellular transport mechanisms were influenced by this antifungal drug leading to osmotic stress as already reported in Altwasser et al. [26]. In addition, we observed the association of ORM cluster components with the (1,3)-alpha-D-glucan biosynthesis as well as carbohydrate metabolic processes. Consistently, caspofungin inhibits the synthesis of β-(1,3)-glucan which is the principal component of the fungal cell wall [55]. As a compensatory response, the production of other cell wall polymers was stimulated. Another interesting finding was the increased production of the secondary metabolite fumagillin upon exposure of *A. fumigatus* to caspofungin. So far, only the release of the secondary metabolite gliotoxin has been reported for cultures of *A. fumigatus* in the presence of caspofungin [56]. Fumagillin has anti-angiogenic activity [57] and induces cell death in erythrocytes [58]. It is therefore possible that administration of caspofungin induces the production of secondary metabolites that have adverse effects on host cells during the infection. Another interesting aspect of our finding is that the induction of fumagillin production upon caspofungin exposure may represent a form of ‘microbial communication’ between fungi, in particular taking into account that echinocandins like caspofungin are produced by a diverse set of fungi [59].

As Wang et al. [13] reported, studying key factors of a drug-induced response by analyzing the underlying network structure may help to better understand the position and dynamics of drug targets and associated proteins potentially involved in drug-caused side effects. Here, in addition to the main target β-(1,3)-D-glucan synthase, we detected polyubiquitin UbiD among the top five nodes of the ORM ranked by both node degree and betweenness centrality. Polyubiquitin is known to encode multiple ubiquitin units in tandem, each of these transcribed as a single transcript. It is involved in several metabolic pathways and plays an important role in the regulation of the proteasome-based protein degradation processes [43, 60]. Some recent studies have already reported the importance of polyubiquitin in the fungal stress response. In the pathogenic yeast *Candida albicans*, Leach et al. [61] have shown that polyubiquitin is required for the adaption to sudden stress induced, e.g., by heat or caspofungin and is critical for the fungus’ pathogenicity. In another study in *S. cerevisiae*, Lesage et al. [62] described ubiquitin-related protein degradation as an important process in the compensation for defects in glucan biosynthesis. We hypothesize that polyubiquitin is an important player in the compensatory response of *A. fumigatus* to caspofungin. In line, the corresponding gene *ubi4* was shown to be induced upon heat-shock in *A. nidulans* [43].

Exemplarily, CBF/NF-Y family transcription factor was detected among the list of TFs. Its *C. albicans* ortholog DPB4 represents a putative DNA polymerase epsilon subunit D and was shown to be involved in filamentous growth and maintenance of the mitochondrial DNA genome [63]. This role in mitochondrial processes in conjunction with caspofungin treatment is in agreement with the in previous studies shown importance of mitochondrial functions for drug tolerance and virulence of fungal pathogens ([47], and references therein). Also for *C. albicans*, Khamooshi et al. [64] have reported that deletion of DPB4 results in a decreased resistance to caspofungin in drop plate assays. These facts could indicate an involvement of CBF/NF-Y family transcription factor in the resistance of *A. fumigatus* to caspofungin*.*

Interestingly, in our study, both the polyubiquitin and the CBF/NF-Y family transcription factor were detected in all transcriptome and, in case of CBF/NF-Y family transcription factor, proteome time points but neither as DEG nor as DSyP. However, their location within the ORM had shown that they are closely related to DEGs, DSyPs or DSePs. Consequently, by considering DEGs, DSyPs or DSePs for data analyses by SA, these proteins would not have been taken into account as factors in the fungal response despite the fact that they likely have a strong influence on DEGs, DSyPs or DSePs as shown in the ORM. To our knowledge, the role of both the polyubiquitin and the CBF/NF-Y family transcription factor has not been examined yet in the context of caspofungin-induced stress in *A. fumigatus.* Hence, our analyses offer novel hypotheses which have to be verified in future studies.

### The module-detecting approach KeyPathwayMiner

In addition to MD, also other approaches identifying regulatory modules are available, for instance, KPM. Similar to MD, KPM can be used for the analyses of both, single-level and multilevel omics data. However, it does not make assumptions about community structures. KPM combines DEGs, DSyPs or DSePs with non-DEG/DSyP/DSeP exception nodes acting as ‘bridges’ to detect maximal connected sub-networks [15]. The comparison of MD- and KPM-generated regulatory modules showed that MD generates modules with a significant higher number of components than KPM. Additionally, these MD module components cover most of the KPM components. As these findings indicate that MD-generated modules are more comprehensive than modules derived by KPM, we focused on the results obtained by MD.

### PPIN information as limiting factor

The basis of module-detecting approaches like MD or KPM is information from underlying organism-specific PPINs. Hence, the quality of results provided by these approaches also depends on the comprehensiveness of the underlying PPIN itself. Only those components of the experimental data which do also occur in the PPIN are considered for the regulatory module. For example, the PPIN of *A. fumigatus* strain A1163 downloaded from STRING consists of 4123 proteins. But according to current information provided by CADRE, the fungus itself is known to comprise 9916 protein-coding genes. Hence, more than half of the known fungal components cannot be considered for analyses based on this PPIN. Consequently, the available PPIN information can be considered as limiting factor in the data analyses. Thus, while our results highlight the benefits and potential provided by the regulatory module detection-based analysis of multilevel omics data, future studies will have to focus on the expansion of organism-specific PPINs.

## Conclusion

PPINs enable the consideration of both structural and functional relationships between network proteins. Thus, they facilitate a focused view on closely related components in terms of modules. In this study, we demonstrated so far untested capacity of the module-detecting MD approach to integrate omics data coming from different molecular levels and time points. Moreover, we showed that this level of integration is not achievable using a simple approach of comparing lists of DEGs/DSyPs/DSePs. The integration of these data in one ORM can provide an overview of the overall organism’s response to an external stimulus. We presented several approaches for analyzing this response and potential key factors contributing to, e.g., drug-caused side effects in more detail. With the aid of the regulatory module-detecting approach, it is possible to identify potential response key factors which cannot be detected in commonly used approaches comparing DEGs, DSyPs and DSePs, exclusively.

## Additional files

Additional file 1: Lists of differentially expressed genes, differentially synthesized proteins and differentially secreted proteins. (XLSX 227 kb)

Additional file 2: Supplementary Materials. (PDF 3110 kb)

Additional file 3: Quantification of the secondary metabolites fumagillin and pseurotin A. (XLSX 21 kb)

Additional file 4: KeyPathwayMiner-generated overall regulatory module and significantly enriched biological processes. (XLSX 55 kb)

Additional file 5: Significantly enriched biological processes of the MD-multi-seed-based overall regulatory module. (XLSX 49 kb)
