# Supplementary material for: Module-detection approaches for the integration of multilevel omics data highlight the comprehensive response of Aspergillus fumigatus to caspofungin
Source: BMC Syst Biol. 2018 Oct 20;12:88. doi: 10.1186/s12918-018-0620-8 (PMC6195963; doi:10.1186/s12918-018-0620-8)
Supplement: Supplementary file 2 — Supplementary Materials. (PDF 3110 kb) [file 12918_2018_620_MOESM2_ESM.pdf]

Supplementary Materials

Module-detection approaches for the integration of multilevel omics data highlight the comprehensive response of *Aspergillus fumigatus* to caspofungin

T. Conrad[1], O. Kniemeyer[2], S. G. Henkel[3], T. Krüger[2], D. J. Mattern[2*], V. Valiante[4], R. Guthke[1], I. D. Jacobsen[5,6], A. A. Brakhage[2,6], S. Vlaic[1] and J. Linde[7,8]

1 Systems Biology/Bioinformatics, Leibniz Institute for Natural Product Research and Infection Biology – Hans Knöll Institute, Jena, Germany
2 Molecular and Applied Microbiology, Leibniz Institute for Natural Product Research and Infection Biology – Hans Knöll Institute, Jena, Germany
3 BioControl Jena GmbH, Jena, Germany
4 Biobricks of Microbial Natural Product Syntheses, Leibniz Institute for Natural Product Research and Infection Biology – Hans Knöll Institute, Jena, Germany
5 Microbial Immunology, Leibniz Institute for Natural Product Research and Infection Biology – Hans Knöll Institute, Jena, Germany
6 Institute for Microbiology, Friedrich Schiller University, Jena, Germany
7 Research Group PiDOMICs, Leibniz Institute for Natural Product Research and Infection Biology – Hans Knöll Institute, Jena, Germany
8 Institute for Bacterial Infections and Zoonoses, Federal Research Institute for Animal Health – Friedrich Loeffler Institute, Jena, Germany

Current address
* PerkinElmer Inc., Rodgau, Germany

Correspondence
Theresia Conrad, Leibniz Institute for Natural Product Research and Infection Biology – Hans Knöll Institute, Jena, Germany. Tel. +49 3641 532 1521; E-mail: Theresia.Conrad@leibniz-hki.de

Contents
1 Single-seed and multi-seed ModuleDiscoverer approach 2
2 Module-detecting approach KeyPathwayMiner 4
3 Supplementary Figures 6
4 Supplementary Tables 8

1 Single-seed and multi-seed ModuleDiscoverer approach

ModuleDiscoverer (MD) provides two different techniques for identifying cliques within the protein-protein interaction network (PPIN): The single-seed approach and the multi-seed approach. The decision of identifying cliques based either on the single- or the multi-seed approach has to be made already in the first step of the MD algorithm in which the approximation of the PPIN's community structure takes place. The term 'single-seed' means that the algorithm starts from only one randomly selected seed node to identify minimal cliques of size three and followed by extending them to maximal cliques which represent the basis of the final regulatory module. As reported in Vlaic *et al.* [1], the single-seed approach favors the enumeration of large maximal cliques in dense regions of highly overlapping cliques. Hence, some proteins, which are only part of small cliques could be missed. Addressing this issue, the multi-seed approach uses two or even more seed nodes to identify cliques. This leads to a breakdown of large maximal cliques by using multiple seeds competing for nodes during the enumeration of cliques. On the one hand, this increases the probability of identifying proteins which are only part of small cliques. On the other hand, the resulting regulatory module contains a higher number of proteins which are not associated to DEGs. Vlaic *et al.* showed that the multi-seed approach produces very similar results to those received by the single-seed approach. In the end, it can be regarded as a comprehensive extension of the single-seed approach due to the additionally considered small-clique-proteins.

Here, we focused on the single-seed approach for two reasons: First, this approach is comparable with other well-established maximal clique enumeration problem-based algorithms (e.g., Barrenäs *et al.* [2] or Gustafsson *et al.* [3]). Second, Vlaic *et al.* showed that the multi-seed identified modules can be essentially considered as an extension of the single-seed modules. Nevertheless, to estimate the comprehensiveness of the single-seed-generated regulatory modules, we performed further analyses and applied the multi-seed approach to the experimental data. The estimation of the required number of seed nodes was based on the application of MD to rat data performed by Vlaic *et al.*. Since the high-confidence (score > 0.7) PPIN of *Aspergillus fumigatus* (4 121 proteins) roughly contains a third of the nodes of the *Rattus norvegicus* network (15 436 proteins) used in the study of Vlaic *et al.* we decided to use 10 seed nodes (roughly a third). Interestingly, Vlaic *et al.* also showed that the values around a chosen number of seed nodes does not significantly impact the overall structure of the resulting regulatory module.

When comparing the regulatory modules received by the single- and the multi-seed approach, we observed that multi-seed-generated modules comprised 100 % of all single-seed-generated modules and also contained an additional number of module components (Supplementary Table 1).

For the multi-seed overall regulatory module (ORM), we performed a generalized topological overlap measurement (as done for the single-seed ORM) to compare significantly enriched biological processes of the multi-seed- with those of the single-seed-based ORM. We found that multi-seed-based ORM clusters are significantly associated with biological processes that are also enriched for single-seed-based clusters. Such processes are, for instance, activation of kinase activity, actin-filament-based processes, response to oxidative stress, carbohydrate metabolic processes, amino acid metabolic processes, transport mechanisms and secondary and lipid metabolic process. The complete lists of significantly enriched biological processes can be found in Additional File 4. By analyzing key factors in the fungal response, we detected β-(1,3)-D-glucan synthase within the ORM, the main target of caspofungin. In addition, we identified the polyubiquitin UbiD among the top five ORM nodes ranked by both node degree and betweenness centrality. There is only a slight multi-/single-seed-difference observable for the UbiD node degree (single-seed: 111, multi-seed: 117) and betweenness centrality (single-seed: 0.396, multi-seed: 0.359). Filtering for transcription factors led to the same results for both ORM's, including the CBF/NF-Y family transcription factor.

In conclusion, the multi-seed MD approach allows for effectively integrating multilevel omics data. It contains the regulatory modules received by the single-seed approach and provides even higher numbers of regulatory module components. The ORM generated by the multi-seed approach confirms the already observed key players and significantly associated processes. Altogether, the multi-seed MD can be considered as an extension of the single-seed MD.

[1] Vlaic S, Conrad T, Tokarski-Schnelle C, Gustafsson M, Dahmen U, Guthke R, *et al. ModuleDiscoverer: Identification of regulatory modules in protein-protein interaction networks.* Sci Rep. 2018;8.

[2] Barrenäs F, Chavali S, Alves AC, Coin L, Jarvelin MR, Jörnsten R, *et al. Highly interconnected genes in disease-specific networks are enriched for disease-associated polymorphisms.* Genome Biol. 2012 Jun 15; 13(6):R46. doi: 10.1186/gb-2012-13-6-r46.

[3] Gustafsson M, Edström M, Gawel D, Nestor CE, Wang H, Zhang H, *et al. Integrated genomic and prospective clinical studies show the importance of modular pleiotropy for disease susceptibility, diagnosis and treatment.* Genome Med. 2014 Feb 26;6(2):17. doi: 10.1186/gm534. ECollection 2014.

2 Module-detecting approach KeyPathwayMiner

Background

The regulatory module-detecting KeyPathwayMiner (KPM) [4] approach was shown to be efficient for both single-level omics data analysis and the integration of multilevel omics data. It detects maximal connected sub-networks by combining differentially expressed genes (DEGs), differentially synthesized proteins (DSyPs) and differentially secreted proteins (DSePs) with non-DEG/DSyP/DSeP exception nodes acting as 'bridges'. KPM does not make assumptions about the protein-protein interaction network's (PPIN's) underlying community structures.

Methods

All analyses performed for the KPM-application are based on methods described in the 'Methods' part of the main manuscript. KPM was applied as described in section 'Application of module-detecting approaches'. Analyses regarding the overlap of molecular levels and time points were described in section 'Comparison of the simple approach and a module-detecting approach'. Details on the GO-term enrichment analysis for the KPM-based ORM were shown in 'Enrichment analysis (functional annotation of biological processes)'.

Results and Discussion

The following analyses are based on the KPM regulatory modules as presented in Table 5 in the main manuscript. Supplementary Figure 1 shows the analysis of the molecular level overlap resulting in highest values for proteome and secretome (9.5 %). We expected this result as proteome and secretome are immediately consecutive levels and are both measured by LC-MS/MS. The maximal overlap of transcriptome, proteome and secretome is 0.4 %. For estimating the best transcriptome-proteome time point match (Supplementary Figure 2), two approaches are tested: (A) comparison of module components, and (B) correlation of the module components' regulation. Comparing only the module components (A), the results show a highest overlap of transcriptome at 1 h and proteome at 4 h (4.3 %). The comparison with the proteome at 8 h results in the best match for the transcriptome at 8 h (6.8 %). Taking into account the module components' regulation (B),

the lowest distance can be observed for transcriptome at 1 h and proteome at 4 h. As this is in agreement with (A) regarding the proteome at 4 h, we could assume a time delay of 3 h in the fungal sub-responses. The much lower distance values regarding all transcriptome time points and the proteome at 4 h (dark green) compared to those with the proteome at 8 h (light green and yellow) point to a stronger involvement of the proteomic sub-response at 4 h than at 8 h. In contrast, the results regarding (A) have shown higher overlap values regarding proteome at 8 h compared to 4 h. Consequently, with the KPM approach it is hard to see a clear tendency regarding the best transcriptome-proteome time point match.

Based on a GO-term enrichment analysis regarding biological processes, we found that the KPM-generated ORM is significantly associated with biological processes which are also enriched for the MD-based ORM. Such processes are, for instance, (1→3)-alpha-glucan biosynthetic process, carbohydrate catabolic process, alpha-amino acid catabolic process, lipid and secondary metabolic processes or oxidation-reduction processes. In Supplementary Figure 3, we compared these exemplarily selected processes regarding their MD- and KPM-based -log10 p-values and observed highly similar values for both approaches. Complete lists of significantly enriched biological processes as well as all components of the KPM-based ORM can be found in Additional File 5.

[4] Alcaraz N, Pauling J, Batra R, Barbosa E, Junge A, Christensen AG, Azevedo V, Ditzel HJ, Baumbach J. *KeyPathwayMiner 4.0: condition-specific pathway analysis by combining multiple omics studies and networks with Cytoscape.* BMC Syst Biol. 2014 Aug 19;8:99. doi: 10.1186/s12918-014-0099-x.

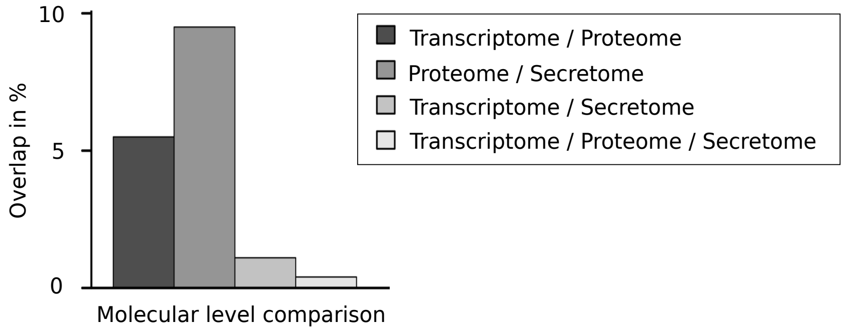

Supplementary Figure 1 | Overlap of molecular levels based on KeyPathwayMiner-received modules.

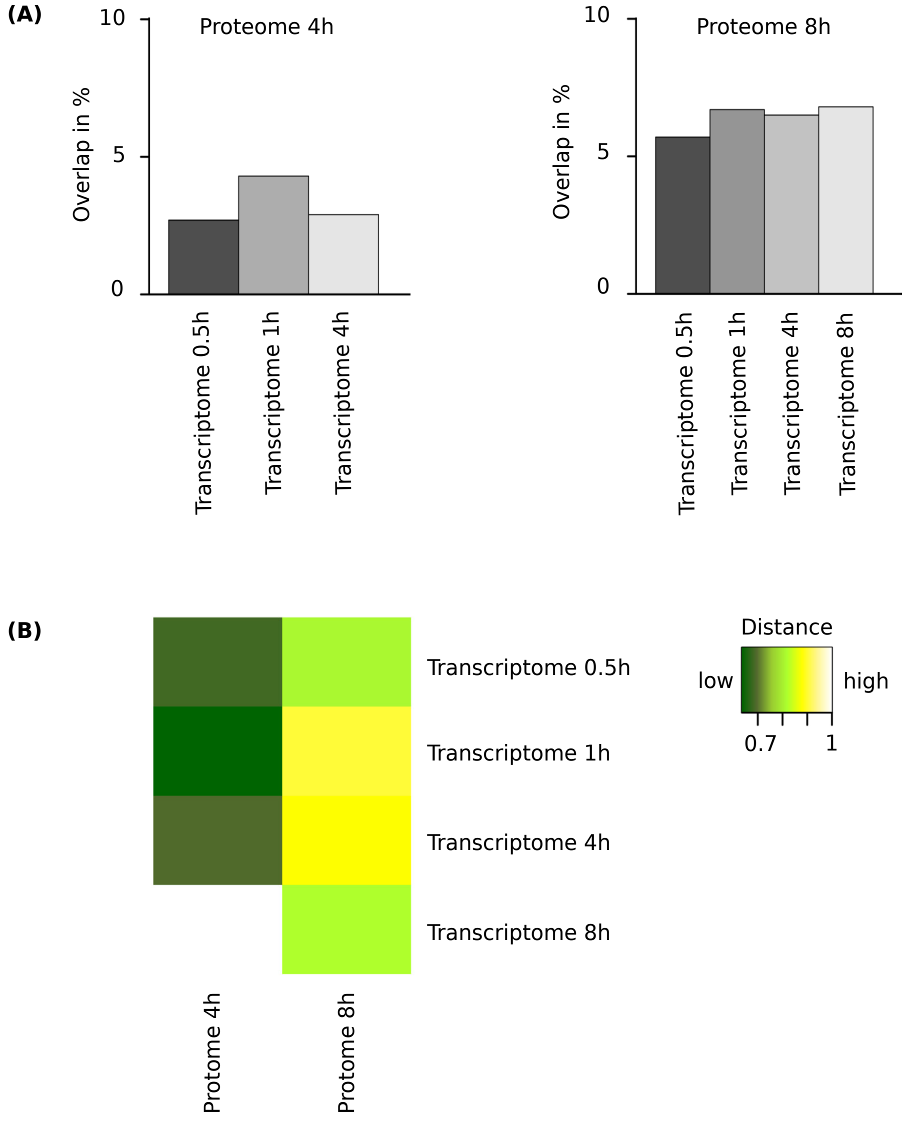

Supplementary Figure 2 | Estimation of the best transcriptome-proteome time point overlap based on **(A)** KeyPathwayMiner-received module components and **(B)** their regulation.

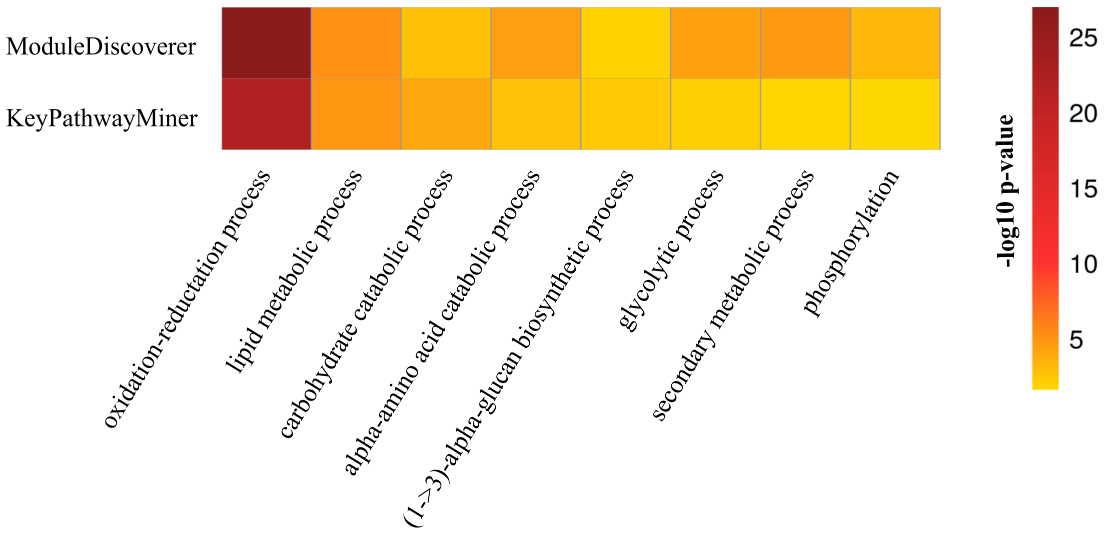

Supplementary Figure 3 | Comparison of ModuleDiscoverer- and KeyPathwayMiner-generated overall regulatory modules regarding exemplarily selected significantly enriched biological processes. Higher values equal lower p-values.

Supplementary Table 1 | Comparison of regulatory modules received by the single-seed (SS) and multi-seed (MS) ModuleDiscoverer approach. The overlap of module components is defined as fraction of the intersection of the respective datasets from the single-seed datasets.

**Underlying experimental dataset**	**Number of components of SS-based modules**	**Number of components of MS-based modules**	**Overlap of module components** (SS-based modules in % of the set of MS-based modules)
Transcriptome 0.5h	511	716	511 (100 %)
Transcriptome 1h	256	345	256 (100 %)
Transcriptome 4h	313	413	313 (100 %)
Transcriptome 8h	256	430	256 (100 %)
Proteome 4h	147	227	147 (100 %)
Proteome 8h	124	213	124 (100 %)
Secretome 8h	293	321	293 (100 %)
Overall regulatory module	894	1119	894 (100 %)

Supplementary Table 2 | All proteins of the ModuleDiscoverer-generated overall regulatory module and their sub-module and cluster membership (see Figure 5)

**CADRE-IDs**	**AspGD-IDs**	**UniProt-IDs**	**Protein names**	**Sub-module**	**Cluster**
CADAFUBP00007020	AFUB_072030	B0Y5B4	Protostadienol synthase A (EC 5.4.99.32)	1	1
CADAFUBP00006723	AFUB_069030	B0Y4G7	Terpene cyclase/mutase family member (EC 5.4.99.-)	1	1
CADAFUBP00005148	AFUB_052590	B0Y314	Terpene cyclase/mutase family member (EC 5.4.99.-)	1	1
CADAFUBP00006971	AFUB_071550	B0Y565	Terpene cyclase/mutase family member (EC 5.4.99.-)	1	1
CADAFUBP00008534	AFUB_087800	B0YBT9	Farnesyl-diphosphate farnesyltransferase, putative	1	1
CADAFUBP00008440	AFUB_086820	B0YBJ5	Terpene cyclase/mutase family member (EC 5.4.99.-)	1	1
CADAFUBP00006672	AFUB_068520	B0Y6Y0	3-ketosteroid reductase	1	1
CADAFUBP00003006	AFUB_030680	B0XU84	C-3 sterol dehydrogenase/C-4 decarboxylase	1	1
CADAFUBP00000428	AFUB_004350	B0XNB3	Cytochrome P450 sterol C-22 desaturase, putative	1	1
CADAFUBP00008412	AFUB_086340	B0YAZ3	O-methyltransferase, putative	1	1
CADAFUBP00005008	AFUB_051170	B0Y2E5	O-methyltransferase, putative	1	1
CADAFUBP00001320	AFUB_013420	B0XRP6	Leucine carboxyl methyltransferase superfamily	1	1
CADAFUBP00009800	AFUB_100930	B0YEY5	O-methyltransferase, putative	1	1
CADAFUBP00007900	NA	NA	NA	1	1
CADAFUBP00005644	AFUB_057690	B0Y0L0	3-demethylubiquinone-9 3-methyltransferase, putative	1	1
CADAFUBP00005401	AFUB_055170	B0Y3R7	UbiE/COQ5 methyltransferase, putative	1	1
CADAFUBP00004613	AFUB_047060	B0XWR6	Homocysteine S-methyltransferase, putative	1	1
CADAFUBP00004523	AFUB_046110	B0Y0G5	Aromatic-L-amino-acid decarboxylase	1	1
CADAFUBP00002939	AFUB_030000	B0XTU8	Prenyl cysteine carboxyl methyltransferase, putative	1	1
CADAFUBP00002439	AFUB_024960	B0XRV4	Arginine N-methyltransferase 2 (EC 2.1.1.-)	1	1
CADAFUBP00001671	AFUB_017040	B0XPL0	Histidinol dehydrogenase (HDH) (EC 1.1.1.23)	1	1
CADAFUBP00001382	AFUB_014100	B0XMT5	Histidine biosynthesis trifunctional protein [Includes: Phosphoribosyl-AMP cyclohydrolase (EC 3.5.4.19); Phosphoribosyl-ATP pyrophosphohydrolase (EC 3.6.1.31); Histidinol dehydrogenase (HDH) (EC 1.1.1.23)]	1	1
CADAFUBP00009696	AFUB_099760	B0YEN1	UbiE/COQ5 methyltransferase, putative	1	1
CADAFUBP00008914	AFUB_091670	B0YB60	UbiE/COQ5 methyltransferase, putative	1	1
CADAFUBP00009382	AFUB_096530	B0YDR7	Cytochrome P450, putative	1	1
CADAFUBP00008191	AFUB_084150	B0YAC2	C-4 methyl sterol oxidase, putative	1	1
CADAFUBP00008330	AFUB_085530	B0YAR1	Sterol desaturase, putative	1	1
CADAFUBP00006945	AFUB_071290	B0Y539	Cytochrome P450 monooxygenase, putative	1	1
CADAFUBP00003370	AFUB_034460	B0XZV0	Cytochrome P450 monooxygenase (Fum15), putative	1	1
CADAFUBP00002483	AFUB_025410	B0XRZ8	Cytochrome P450 family protein, putative	1	1
CADAFUBP00000757	AFUB_007690	B0XPV3	Cytochrome P450, putative	1	1
CADAFUBP00007026	AFUB_072090	B0Y5C0	Cytochrome P450 monooxygenase, putative	1	1
CADAFUBP00007897	AFUB_081090	B0Y9H8	Cytochrome P450 alkane hydroxylase	1	1
CADAFUBP00007021	AFUB_072040	B0Y5B5	Cytochrome P450 monooxygenase, putative	1	1
CADAFUBP00000737	AFUB_007490	B0XPT3	C-24(28) sterol reductase	1	1
CADAFUBP00009660	AFUB_099400	B0YEJ5	Sterol 24-C-methyltransferase (EC 2.1.1.41) (Delta(24)-sterol C-methyltransferase)	1	1
CADAFUBP00006453	AFUB_066290	B0Y6B1	S-adenosyl-methionine-sterol-C-methyltransferas	1	1
CADAFUBP00000500	AFUB_005060	B0XNQ0	C-8 sterol isomerase (Erg-1), putative	1	1
CADAFUBP00000383	AFUB_003900	B0XN01	Uncharacterized protein	1	1
CADAFUBP00004314	AFUB_043980	B0XZD0	Cytochrome P450 monooxygenase, putative	1	1
CADAFUBP00006063	AFUB_062080	B0Y2J9	C-24(28) sterol reductase	1	1
CADAFUBP00001779	AFUB_018240	B0XT71	C-4 methylsterol oxidase, putative	1	1
CADAFUBP00002944	AFUB_030050	B0XU22	Cytochrome p450, putative	1	1
CADAFUBP00000598	AFUB_006090	B0XP65	C-14 sterol reductase	1	1
CADAFUBP00006528	AFUB_067090	B0Y6I6	Toxin biosynthesis cytochrome P450 monooxygenase, putative	1	1
CADAFUBP00002084	AFUB_021350	B0XV19	Cytochrome P450 monooxygenase, putative	1	1
CADAFUBP00005639	AFUB_057640	B0Y0K5	Cytochrome P450 monooxygenase, putative	1	1

CADAFUBP00009642	AFUB_099220	B0YEH7	Cytochrome P450 alkane hydroxylase, putative	1	1
CADAFUBP00009538	AFUB_098170	B0YE73	C-4 methyl sterol oxidase (Erg25), putative (Fragment)	1	1
CADAFUBP00003242	AFUB_033070	B0XVN5	C-3 sterol dehydrogenase/C-4 decarboxylase family protein	1	1
CADAFUBP00002669	AFUB_027300	B0XSI4	Ergosterol biosynthesis protein Erg28, putative	1	1
CADAFUBP00001696	AFUB_017380	B0XSS0	Sterol delta 5,6-desaturase, putative	1	1
CADAFUBP00000349	AFUB_003560	B0XMW7	C-14 sterol reductase	1	1
CADAFUBP00007385	AFUB_075810	B0Y820	Cytochrome P450 monooxygenase, putative	1	1
CADAFUBP00006108	AFUB_062540	B0Y2V9	Cytochrome P450 oxygenase, putative	1	1
CADAFUBP00000204	AFUB_002090	B0XRD5	Cytochrome P450 alkane hydroxylase, putative	1	1
CADAFUBP00004549	AFUB_046390	B0Y0J1	Cytochrome P450 monooxygenase, putative	1	1
CADAFUBP00009051	AFUB_093140	B0YCT6	Sterol delta 5,6-desaturase ERG3	1	1
CADAFUBP00005006	AFUB_051150	B0Y2E3	Sporulation-specific N-formyltyrosine oxidase Dit2, putative	1	1
CADAFUBP00007249	AFUB_074420	B0Y7N4	Cytochrome P450 alkane hydroxylase, putative	1	1
CADAFUBP00004878	AFUB_049870	B0Y1M9	Cytochrome P450, putative	1	1
CADAFUBP00008678	AFUB_089270	B0YC83	14-alpha sterol demethylase Cyp51B	1	1
CADAFUBP00008175	AFUB_083980	B0YAA6	Cytochrome P450 monooxygenase, putative	1	1
CADAFUBP00006222	AFUB_063960	B0Y5N0	14-alpha sterol demethylase Cyp51A	1	1
CADAFUBP00004319	AFUB_044030	B0XZD5	Cytochrome P450 monooxygenase, putative	1	1
CADAFUBP00003829	AFUB_039100	B0XXZ5	Serine/threonine protein kinase, putative	1	2
CADAFUBP00000666	AFUB_006780	B0XPK3	cAMP-dependent protein kinase-like, putative	1	2
CADAFUBP00003246	AFUB_033110	B0XVN9	2-amino-3-carboxymuconate-6-semialdehyde decarboxylase, putative	1	2
CADAFUBP00005560	AFUB_056780	B0Y476	Superoxide dismutase [Cu-Zn] (EC 1.15.1.1)	1	2
CADAFUBP00007326	AFUB_075210	B0Y7W1	Protein kinase, putative	1	2
CADAFUBP00002580	AFUB_026400	B0XS95	Serine/threonine protein kinase (YPK1), putative	1	2
CADAFUBP00008068	AFUB_082850	B0Y9Z9	3-hydroxyanthranilate 3,4-dioxygenase 1 (EC 1.13.11.6) (3-hydroxyanthranilate oxygenase 1) (3-HAO-1) (3-hydroxyanthranilic acid dioxygenase 1) (HAD-1) (Biosynthesis of nicotinic acid protein 1-1)	1	2
CADAFUBP00006514	AFUB_066950	B0Y6H2	Kynureninase 2 (EC 3.7.1.3) (Biosynthesis of nicotinic acid protein 5-2) (L-kynurenine hydrolase 2)	1	2
CADAFUBP00003247	AFUB_033120	B0XVP0	3-hydroxyanthranilate 3,4-dioxygenase 2 (EC 1.13.11.6) (3-hydroxyanthranilate oxygenase 2) (3-HAO-2) (3-hydroxyanthranilic acid dioxygenase 2) (HAD-2) (Biosynthesis of nicotinic acid protein 1-2)	1	2
CADAFUBP00002557	AFUB_026170	B0XS72	Kynureninase 1 (EC 3.7.1.3) (Biosynthesis of nicotinic acid protein 5-1) (L-kynurenine hydrolase 1)	1	2
CADAFUBP00007122	AFUB_073150	B0Y7A7	Fe superoxide dismutase, putative	1	2
CADAFUBP00006681	AFUB_068610	B0Y6Y9	Superoxide dismutase (EC 1.15.1.1)	1	2
CADAFUBP00001380	AFUB_014080	B0XMT3	Superoxide dismutase (EC 1.15.1.1)	1	2
CADAFUBP00009176	AFUB_094400	B0YD61	Catalase (EC 1.11.1.6)	1	2
CADAFUBP00004518	AFUB_046060	B0Y0G0	Catalase (EC 1.11.1.6)	1	2
CADAFUBP00003305	AFUB_033720	B0XW13	Catalase (EC 1.11.1.6)	1	2
CADAFUBP00001686	AFUB_017280	B0XSR0	Catalase, putative	1	2
CADAFUBP00007658	AFUB_078570	B0Y8U3	EH domain binding protein epsin 2	1	3
CADAFUBP00004209	AFUB_042910	B0XZ25	Cytoskeleton assembly control protein Sla2, putative	1	3
CADAFUBP00002067	AFUB_021180	B0XUT3	ENTH domain protein	1	3
CADAFUBP00008690	AFUB_089400	B0YC95	Actin cytoskeleton-regulatory complex protein pan1	1	3
CADAFUBP00008080	AFUB_082970	B0YA11	Cytoskeleton assembly control protein Sla1, putative	1	3
CADAFUBP00006305	AFUB_064800	B0Y5W3	Clathrin heavy chain	1	3
CADAFUBP00001229	AFUB_012500	B0XR88	Actin cytoskeleton-regulatory complex protein end3 (Cytoskeletal adapter protein sagA) (Endocytosis protein 3)	1	3
CADAFUBP00000589	AFUB_006010	B0XP56	Protein phosphatase	1	3
CADAFUBP00005796	AFUB_059230	B0Y112	Protein-vacuolar targeting protein Atg18, putative	1	3
CADAFUBP00007525	AFUB_077210	B0Y8G0	Protein kinase (VPS15), putative	1	3
CADAFUBP00003168	AFUB_032320	B0XV94	SacI domain protein	1	3
CADAFUBP00008672	AFUB_089210	B0YC77	SacI domain and	1	3

			endonuclease/exonuclease/phosphatase family protein		
CADAFUBP00006340	AFUB_065150	B0Y5Z8	Phosphoinositide phosphatase (Sac1), putative	1	3
CADAFUBP00006933	AFUB_071170	B0Y527	Hsp70 family protein	1	3
CADAFUBP00001187	AFUB_012080	B0XR33	Hsp70 chaperone Hsp88	1	3
CADAFUBP00007149	AFUB_073420	B0Y7D4	1-phosphatidylinositol-3-phosphate 5-kinase (Fab1), putative	1	3
CADAFUBP00001512	AFUB_015420	B0XNK0	SacI domain protein	1	3
CADAFUBP00000180	AFUB_001850	B0XM08	Vacuole-associated enzyme activator complex component (Vac14), putative	1	3
CADAFUBP00007937	AFUB_081510	B0Y9L8	FMN dependent dehydrogenase, putative (EC 1.13.12.-)	1	4
CADAFUBP00009720	AFUB_100010	B0YEQ5	Mitochondrial cytochrome b2, putative	1	4
CADAFUBP00004211	AFUB_042930	B0XZ27	Hexaprenyl pyrophosphate synthetase Coq1, putative	1	4
CADAFUBP00009523	AFUB_097930	B0YE58	FMN dependent dehydrogenase, putative	1	4
CADAFUBP00008195	NA	NA	NA	1	4
CADAFUBP00006247	AFUB_064220	B0Y5Q5	Diphosphomevalonate decarboxylase (EC 4.1.1.33) (Mevalonate pyrophosphate decarboxylase)	1	4
CADAFUBP00001243	AFUB_012640	B0XRA2	Geranylgeranyl diphosphate synthase	1	4
CADAFUBP00004904	AFUB_050130	B0Y1X1	Geranylgeranyl pyrophosphate synthase, putative	1	4
CADAFUBP00007386	AFUB_075820	B0Y821	Geranylgeranyl diphosphate synthase, putative	1	4
CADAFUBP00004989	AFUB_050980	B0Y2C6	Farnesyl-pyrophosphate synthetase	1	4
CADAFUBP00004738	AFUB_048360	B0XXH6	Short chain alpha-hydroxy acid oxidase, putative (EC 1.1.3.15)	1	4
CADAFUBP00005003	AFUB_051120	B0Y2E0	20S cyclosome subunit (Cut9/Cdc16), putative	1	5
CADAFUBP00000607	AFUB_006190	B0XPE4	MAP kinase kinase (Mkk2), putative	1	5
CADAFUBP00009550	AFUB_098290	B0YE85	Protein tyrosine phosphatase (Pyp1), putative	1	5
CADAFUBP00004231	AFUB_043130	B0XZ47	MAP kinase kinase Ste7	1	5
CADAFUBP00001017	AFUB_010360	B0XQL3	MAP kinase kinase kinase SskB, putative	1	5
CADAFUBP00003782	AFUB_038630	B0XXU8	Protein serine/threonine kinase (Ran1), putative	1	5
CADAFUBP00001221	AFUB_012420	B0XR80	Mitogen-activated protein kinase (EC 2.7.11.24)	1	5
CADAFUBP00007653	AFUB_078520	B0Y8T8	Stress response regulator/HFS transcription factor, putative	1	5
CADAFUBP00008762	AFUB_090130	B0YCG7	Telomerase reverse transcriptase, putative	1	5
CADAFUBP00005826	AFUB_059540	B0Y142	Protein kinase C (EC 2.7.11.13)	1	5
CADAFUBP00002548	AFUB_026080	B0XS63	TOR pathway phosphatidylinositol 3-kinase TorA, putative	1	5
CADAFUBP00005134	AFUB_052450	B0Y300	Protein kinase, putative	1	5
CADAFUBP00001347	AFUB_013740	B0XMQ0	Mitochondrial processing peptidase beta subunit, putative	1	6
CADAFUBP00003998	AFUB_040830	B0XYG4	Pyruvate dehydrogenase complex component Pdx1, putative	1	6
CADAFUBP00001483	AFUB_015130	B0XNH1	Succinate dehydrogenase subunit CybS, putative	1	6
CADAFUBP00001519	AFUB_015490	B0XNK7	Glutathione reductase	1	6
CADAFUBP00004270	AFUB_043520	B0XZ86	Cystathionine gamma-synthase, putative	1	6
CADAFUBP00004629	AFUB_047230	B0XX00	Cystathionine beta-lyase	1	6
CADAFUBP00002565	AFUB_026250	B0XS80	Prephenate dehydrogenase	1	6
CADAFUBP00005182	AFUB_052930	B0Y348	NADH-ubiquinone oxidoreductase, subunit G, putative	1	6
CADAFUBP00005162	AFUB_052730	B0Y328	Ubiquinol-cytochrome C reductase complex core protein 2, putative	1	6
CADAFUBP00002199	AFUB_022520	B0XVK0	NADH-ubiquinone oxidoreductase 18 kDa subunit, putative	1	6
CADAFUBP00000240	AFUB_002450	B0XRH1	Cytochrome C1/Cyt1, putative	1	6
CADAFUBP00005212	AFUB_053230	B0Y378	Chorismate mutase/prephenate dehydratase	1	6
CADAFUBP00007286	AFUB_074790	B0Y7S1	2-oxoisovalerate dehydrogenase complex alpha subunit, putative	1	6
CADAFUBP00008548	AFUB_087940	B0YBV3	Thiosulfate sulfurtransferase, putative	1	6
CADAFUBP00004640	AFUB_047340	B0XX11	Tyrosinase, putative	1	6
CADAFUBP00001648	AFUB_016800	B0XPC0	Tyrosinase	1	6
CADAFUBP00004100	AFUB_041830	B0XYR6	Phosphopantothenate-cysteine ligase, putative	1	6
CADAFUBP00008255	AFUB_084800	B0YAI6	Thiosulfate sulfurtransferase, putative	1	6
CADAFUBP00009626	AFUB_099060	B0YEG1	Cystathionine beta-lyase MetG	1	6

CADAFUBP00008571	AFUB_088170	B0YBX6	Cystathionine gamma-synthase	1	6
CADAFUBP00008098	AFUB_083200	B0YA29	Cystathionine gamma-lyase	1	6
CADAFUBP00006068	AFUB_062130	B0Y2K4	Cysteine dioxygenase, putative	1	6
CADAFUBP00004273	AFUB_043550	B0XZ89	Glutamate carboxypeptidase, putative	1	6
CADAFUBP00000581	AFUB_005910	B0XP48	Cysteine dioxygenase	1	6
CADAFUBP00007180	AFUB_073730	B0Y7G5	Alanine aminotransferase, putative	1	6
CADAFUBP00005203	AFUB_053140	B0Y369	Aspartokinase (EC 2.7.2.4)	1	6
CADAFUBP00004387	AFUB_044720	B0XZP0	4-hydroxyphenylpyruvate dioxygenase, putative	1	6
CADAFUBP00003365	AFUB_034410	B0XZU5	4-hydroxyphenylpyruvate dioxygenase, putative	1	6
CADAFUBP00002868	AFUB_029280	B0XTG0	Aromatic aminotransferase Aro8, putative	1	6
CADAFUBP00001336	AFUB_013630	B0XMN9	Histidinol-phosphate aminotransferase	1	6
CADAFUBP00001080	AFUB_011000	B0XQS6	4-hydroxyphenylpyruvate dioxygenase, putative	1	6
CADAFUBP00009629	AFUB_099090	B0YEG4	Cysteine synthase B, putative	1	6
CADAFUBP00008016	AFUB_082320	B0Y9U7	Cysteine synthase, putative	1	6
CADAFUBP00006270	AFUB_064450	B0Y5S8	5-methyltetrahydropteroyltriglutamate--homocysteine S-methyltransferase	1	6
CADAFUBP00006012	AFUB_061550	B0Y277	Transulfuration enzyme family protein, putative	1	6
CADAFUBP00004962	AFUB_050710	B0Y229	Cysteine synthase (O-acetylserine (Thiol)-lyase) (Csase)	1	6
CADAFUBP00006575	AFUB_067560	B0Y6N3	Homocitrate synthase	1	6
CADAFUBP00000385	AFUB_003920	B0XN03	ATP synthase subunit gamma	1	6
CADAFUBP00007250	AFUB_074430	B0Y7N5	Glycerol kinase, putative	1	6
CADAFUBP00006676	AFUB_068560	B0Y6Y4	Glycerol kinase, putative	1	6
CADAFUBP00002543	AFUB_026020	B0XS58	Glycerol dehydrogenase, putative	1	6
CADAFUBP00000980	AFUB_009990	B0XQH6	Glycerol-3-phosphate phosphatase (GppA), putative	1	6
CADAFUBP00005166	AFUB_052770	B0Y332	Homocysteine synthase CysD	1	6
CADAFUBP00003634	AFUB_037120	B0XX79	Methionine synthase, vitamin-B12 independent, putative	1	6
CADAFUBP00000936	AFUB_009540	B0XQD2	Adenosylhomocysteinase (EC 3.3.1.1)	1	6
CADAFUBP00001277	AFUB_012980	B0XRK3	Spermidine synthase	1	6
CADAFUBP00005528	AFUB_056460	B0Y444	Isovaleryl-CoA dehydrogenase IvdA, putative	1	6
CADAFUBP00007495	AFUB_076910	B0Y8D0	Acyl-CoA dehydrogenase family protein	1	6
CADAFUBP00004558	AFUB_046490	B0XWL1	Acyl-CoA dehydrogenase, putative	1	6
CADAFUBP00009789	AFUB_100820	B0YEX4	Succinyl-CoA synthetase beta subunit, putative	1	6
CADAFUBP00005256	AFUB_053690	B0Y3C2	Succinyl-CoA synthetase alpha subunit, putative	1	6
CADAFUBP00006100	AFUB_062460	B0Y2N6	Phosphoenolpyruvate synthase, putative	1	6
CADAFUBP00007568	AFUB_077640	B0Y8K3	Formyltetrahydrofolate deformylase, putative	1	6
CADAFUBP00008822	AFUB_090740	B0YCM7	Fumarylacetoacetate hydrolase family protein	1	6
CADAFUBP00007489	AFUB_076850	B0Y8C4	Homogentisate 1,2-dioxygenase, putative	1	6
CADAFUBP00002079	AFUB_021300	B0XV14	Fumarylacetoacetate hydrolase FahA	1	6
CADAFUBP00002078	AFUB_021290	B0XV13	Homogentisate 1,2-dioxygenase (HmgA), putative	1	6
CADAFUBP00008269	AFUB_084930	B0YAK0	Catalase-peroxidase (CP) (EC 1.11.1.21) (Peroxidase/catalase)	1	6
CADAFUBP00007981	AFUB_081980	B0Y9R2	Acetyl-coA hydrolase Ach1, putative	1	6
CADAFUBP00003760	AFUB_038380	B0XXS6	Probable acetate kinase (EC 2.7.2.1) (Acetokinase)	1	6
CADAFUBP00002867	AFUB_029270	B0XTF9	Thiamine pyrophosphate enzyme, putative	1	6
CADAFUBP00004139	AFUB_042220	B0XYV5	Aspartate-semialdehyde dehydrogenase	1	6
CADAFUBP00006239	AFUB_064140	B0Y5P7	L-lactate dehydrogenase	1	6
CADAFUBP00005733	AFUB_058610	B0Y0U9	Pantoate--beta-alanine ligase	1	6
CADAFUBP00007571	AFUB_077670	B0Y8K6	Thiamine pyrophosphate enzyme, putative	1	6
CADAFUBP00009633	AFUB_099130	B0YEG8	Uncharacterized protein	1	6
CADAFUBP00008612	AFUB_088580	B0YC17	Indoleamine 2,3-dioxygenase family protein	1	6
CADAFUBP00009450	AFUB_097230	B0YDY5	Aminotransferase, putative	1	6
CADAFUBP00006590	AFUB_067710	B0Y6P8	4-hydroxyphenylpyruvate dioxygenase	1	6
CADAFUBP00002076	AFUB_021270	B0XV11	4-hydroxyphenylpyruvate dioxygenase	1	6
CADAFUBP00005265	AFUB_053780	B0Y3D1	Alcohol dehydrogenase, putative	1	6
CADAFUBP00001767	AFUB_018120	B0XT59	S-(hydroxymethyl)glutathione dehydrogenase (EC 1.1.1.284)	1	6

CADAFUBP00004736	AFUB_048340	B0XXH4	Amine oxidase (EC 1.4.3.-)	1	6
CADAFUBP00008512	AFUB_087590	B0YBR7	Alcohol dehydrogenase, putative	1	6
CADAFUBP00008013	AFUB_082290	B0Y9U4	2-hydroxyphytanoyl-CoA lyase, putative	1	6
CADAFUBP00003134	AFUB_031970	B0XUZ5	Dihydroxy acid dehydratase, putative	1	6
CADAFUBP00000756	AFUB_007680	B0XPV2	Dihydroxy-acid dehydratase, putative	1	6
CADAFUBP00000389	AFUB_003960	B0XN07	Mitochondrial dihydroxy acid dehydratase, putative	1	6
CADAFUBP00006349	AFUB_065240	B0Y607	Threonine dehydratase (EC 4.3.1.19) (Threonine deaminase)	1	6
CADAFUBP00006316	AFUB_064910	B0Y5X4	L-serine dehydratase, putative	1	6
CADAFUBP00006255	AFUB_064300	B0Y5R3	Mitochondrial acetolactate synthase small subunit, putative	1	6
CADAFUBP00003803	AFUB_038840	B0XXW9	Acetolactate synthase (EC 2.2.1.6)	1	6
CADAFUBP00003399	AFUB_034750	B0XZX9	Pyridoxal-phosphate dependent enzyme, putative	1	6
CADAFUBP00003398	AFUB_034740	B0XZX8	Ketol-acid reductoisomerase	1	6
CADAFUBP00000641	AFUB_006530	B0XPH8	L-serine dehydratase, putative	1	6
CADAFUBP00005292	AFUB_054050	B0Y3F8	Acyl-CoA dehydrogenase family protein	1	6
CADAFUBP00007206	AFUB_073990	B0Y7J1	General amidase GmdA, putative	1	6
CADAFUBP00005551	AFUB_056690	B0Y467	Amidase, putative	1	6
CADAFUBP00004784	AFUB_048920	B0Y173	General amidase GmdB	1	6
CADAFUBP00001413	AFUB_014420	B0XN33	N-acylethanolamine amidohydrolase, putative	1	6
CADAFUBP00009212	AFUB_094780	B0YD97	Flavin containing polyamine oxidase, putative	1	6
CADAFUBP00008722	AFUB_089720	B0YCC7	Amine oxidase (EC 1.4.3.-)	1	6
CADAFUBP00005609	AFUB_057270	B0Y4C5	Flavin containing polyamine oxidase, putative	1	6
CADAFUBP00005376	AFUB_054910	B0Y3P2	Amine oxidase (EC 1.4.3.-)	1	6
CADAFUBP00004892	AFUB_050010	B0Y1P3	Amine oxidase (EC 1.4.3.-)	1	6
CADAFUBP00004679	AFUB_047750	B0XX50	Amine oxidase (EC 1.4.3.-)	1	6
CADAFUBP00003388	AFUB_034640	B0XZW8	Amine oxidase (EC 1.4.3.-)	1	6
CADAFUBP00001272	AFUB_012930	B0XRJ8	Amine oxidase (EC 1.4.3.-)	1	6
CADAFUBP00009400	AFUB_096720	B0YDT5	Pyruvate decarboxylase, putative	1	6
CADAFUBP00008205	AFUB_084300	B0YAD6	Aldehyde dehydrogenase ALDH	1	6
CADAFUBP00006394	AFUB_065690	B0Y652	Aldehyde dehydrogenase Ald3, putative	1	6
CADAFUBP00006102	AFUB_062480	B0Y2N8	Pyruvate decarboxylase, putative	1	6
CADAFUBP00003729	AFUB_038070	B0XXN9	Pyruvate decarboxylase PdcA, putative	1	6
CADAFUBP00003650	AFUB_037280	B0XX95	Oxidoreductase, zinc-binding	1	6
CADAFUBP00000900	AFUB_009190	B0XQ96	Aldehyde reductase (AKR1), putative	1	6
CADAFUBP00008511	AFUB_087580	B0YBR6	Aldehyde dehydrogenase, putative	1	6
CADAFUBP00007548	AFUB_077440	B0Y8I3	Aldehyde dehydrogenase AldA, putative	1	6
CADAFUBP00007509	AFUB_077050	B0Y8E4	3-hydroxyisobutyrate dehydrogenase	1	6
CADAFUBP00006797	AFUB_069770	B0Y4P1	Methylmalonate-semialdehyde dehydrogenase, putative	1	6
CADAFUBP00005661	AFUB_057860	B0Y0M7	Oxidoreductase, acting on the CH-OH group of donors, NAD or NADP as acceptor	1	6
CADAFUBP00005311	AFUB_054240	B0Y3H7	4-aminobutyrate transaminase GatA	1	6
CADAFUBP00004867	AFUB_049750	B0Y1L8	Oxidoreductase, putative	1	6
CADAFUBP00001736	AFUB_017800	B0XT28	Aldehyde dehydrogenase, putative	1	6
CADAFUBP00002389	AFUB_024460	B0XWD8	50S ribosomal protein L14	1	6
CADAFUBP00000806	AFUB_008170	B0XQ02	Glycerol-3-phosphate dehydrogenase (EC 1.1.5.3)	1	6
CADAFUBP00002922	AFUB_029830	B0XTT1	Mitochondrial dihydroxy acid dehydratase, putative	1	6
CADAFUBP00002827	AFUB_028880	B0XTB9	Tryptophan synthase (EC 4.2.1.20)	1	6
CADAFUBP00000120	AFUB_001220	B0XM82	Tryptophan synthase	1	6
CADAFUBP00008004	AFUB_082200	B0Y9T5	Methylenetetrahydrofolate dehydrogenase	1	6
CADAFUBP00001427	AFUB_014560	B0XN47	2-isopropylmalate synthase	1	6
CADAFUBP00004308	AFUB_043920	B0XZC4	Coenzyme A transferase, putative	1	6
CADAFUBP00005942	AFUB_060830	B0Y1U0	Protein BCP1	1	6
CADAFUBP00001998	AFUB_020450	B0XUE5	Alkaline serine protease	1	6
CADAFUBP00006720	AFUB_069000	B0Y4G4	2-oxo acid dehydrogenases acyltransferase, putative	1	6
CADAFUBP00002529	AFUB_025880	B0XS44	Carbamoyl-phosphate synthase, large subunit	1	6
CADAFUBP00008385	AFUB_086070	B0YAW6	Acetate-CoA ligase, putative	1	6

CADAFUBP00002325	AFUB_023820	B0XW74	Acyl-CoA synthetase, putative	1	6
CADAFUBP00005695	AFUB_058230	B0Y0R1	Ubiquinol-cytochrome c reductase iron-sulfur subunit	1	6
CADAFUBP00004281	AFUB_043630	B0XZ97	Dihydrolipoamide succinyltransferase, putative	1	6
CADAFUBP00005930	AFUB_060710	B0Y1S8	Snf1 protein kinase complex subunit Snf4, putative	1	6
CADAFUBP00008812	AFUB_090640	B0YCL7	FAD dependent oxidoreductase, putative	1	6
CADAFUBP00005321	AFUB_054340	B0Y3I7	Carbamoyl-phosphate synthase, small subunit	1	6
CADAFUBP00000015	AFUB_000150	B0XMG3	4-aminobutyrate aminotransferase, putative (EC 2.6.1.19)	1	6
CADAFUBP00007986	AFUB_082030	B0Y9R7	Aldehyde dehydrogenase family protein	1	6
CADAFUBP00003332	AFUB_034080	B0XZJ4	Isocitrate dehydrogenase family protein	1	6
CADAFUBP00007037	AFUB_072290	B0Y722	Isocitrate dehydrogenase [NAD] subunit, mitochondrial (EC 1.1.1.41)	1	6
CADAFUBP00002641	AFUB_027020	B0XSF6	3-isopropylmalate dehydratase (EC 4.2.1.33) (Alpha-IPM isomerase) (Isopropylmalate isomerase)	1	6
CADAFUBP00007855	AFUB_080660	B0Y9D6	Aconitase family protein	1	6
CADAFUBP00006687	AFUB_068670	B0Y6Z5	Alpha-ketoglutarate dehydrogenase complex subunit Kgd1, putative	1	6
CADAFUBP00007028	AFUB_072110	B0Y5C2	Extracellular 3-ketosteroid 1-dehydrogenase, putative	1	6
CADAFUBP00001501	AFUB_015310	B0XNI9	3-isopropylmalate dehydrogenase (EC 1.1.1.85)	1	6
CADAFUBP00000113	AFUB_001150	B0XM71	3-isopropylmalate dehydrogenase (EC 1.1.1.85)	1	6
CADAFUBP00003961	AFUB_040440	B0XYC7	C1 tetrahydrofolate synthase, putative	1	6
CADAFUBP00000176	AFUB_001810	B0XM04	Aconitate hydratase, mitochondrial (Aconitase) (EC 4.2.1.-)	1	6
CADAFUBP00006632	AFUB_068120	B0Y6U0	Acetyl-coenzyme A synthetase (EC 6.2.1.1)	1	6
CADAFUBP00004298	AFUB_043800	B0XZB4	Pyruvate dehydrogenase E1 beta subunit PdbA, putative	1	6
CADAFUBP00004602	AFUB_046950	B0XWQ5	3-methyl-2-oxobutanoate dehydrogenase, putative	1	6
CADAFUBP00004108	AFUB_041910	B0XYS4	Extracellular developmental signal biosynthesis protein FluG	1	6
CADAFUBP00007143	AFUB_073360	B0Y7C8	Isocitrate dehydrogenase LysB	1	6
CADAFUBP00000704	AFUB_007160	B0XPQ0	CBS and PB1 domain protein	1	6
CADAFUBP00000444	AFUB_004500	B0XNC9	Tartrate dehydrogenase, putative	1	6
CADAFUBP00006101	AFUB_062470	B0Y2N7	Lactate dehydrogenase	1	6
CADAFUBP00002766	AFUB_028270	B0XSY9	NADPH:adrenodoxin oxidoreductase, mitochondrial (EC 1.18.1.6)	1	6
CADAFUBP00005397	AFUB_055120	B0Y3R3	Pyruvate carboxylase, putative	1	6
CADAFUBP00004018	AFUB_041030	B0XYI4	Aconitate hydratase, putative	1	6
CADAFUBP00008220	AFUB_084450	B0YAF1	Aldehyde dehydrogenase family protein	1	6
CADAFUBP00006352	AFUB_065270	B0Y610	Succinate-semialdehyde dehydrogenase Uga2, putative	1	6
CADAFUBP00004107	AFUB_041900	B0XYS3	Succinate-semialdehyde dehydrogenase, putative	1	6
CADAFUBP00009316	AFUB_095870	B0YDK1	Fumarate hydratase, putative	1	6
CADAFUBP00008650	AFUB_088980	B0YC55	Malic enzyme	1	6
CADAFUBP00002368	AFUB_024247	B0XWB7	Malic enzyme	1	6
CADAFUBP00009192	AFUB_094570	B0YD77	2-methylcitrate dehydratase, putative	1	6
CADAFUBP00002865	AFUB_029250	B0XTF7	Uncharacterized protein	1	6
CADAFUBP00003960	AFUB_040430	B0XYC6	Isocitrate dehydrogenase [NADP] (EC 1.1.1.42)	1	6
CADAFUBP00003062	AFUB_031240	B0XUK8	Sulfite reductase, putative	1	6
CADAFUBP00001206	AFUB_012270	B0XR65	Isocitrate dehydrogenase [NAD] subunit, mitochondrial (EC 1.1.1.41)	1	6
CADAFUBP00001863	AFUB_019100	B0XTM3	Formamidase FmdS	1	6
CADAFUBP00000010	AFUB_000100	B0XMF8	Tartrate dehydrogenase, putative (EC 1.1.1.93)	1	6
CADAFUBP00003422	AFUB_034980	B0Y070	Indoleamine 2,3-dioxygenase subfamily	1	6
CADAFUBP00006513	AFUB_066940	B0Y6H1	Indoleamine 2,3-dioxygenase pyrrole 2,3-dioxygenase	1	6
CADAFUBP00003909	AFUB_039920	B0XY75	Bifunctional cytochrome P450/NADPH--P450 reductase [Includes: Cytochrome P450 (EC 1.14.14.1); NADPH--cytochrome P450 reductase (EC 1.6.2.4)]	1	6
CADAFUBP00000467	AFUB_004730	B0XNF2	Cytochrome P450 monooxygenase, putative	1	6
CADAFUBP00008321	AFUB_085440	B0YAQ2	Tartrate dehydrogenase, putative	1	6

CADAFUBP00005524	AFUB_056420	B0Y440	Homoaconitase, mitochondrial (EC 4.2.1.36) (Homoaconitate hydratase)	1	6
CADAFUBP00000707	AFUB_007190	B0XPQ3	Aconitate hydratase, mitochondrial (Aconitase) (EC 4.2.1.-)	1	6
CADAFUBP00000127	AFUB_001300	B0XM85	Nitrilase	1	6
CADAFUBP00009651	AFUB_099310	B0YEI6	Uncharacterized protein	1	6
CADAFUBP00009210	AFUB_094760	B0YD95	Glutamine synthetase	1	6
CADAFUBP00008779	AFUB_090300	B0YCI4	Gamma-glutamyltranspeptidase	1	6
CADAFUBP00007935	AFUB_081480	B0Y9L6	Glutamate decarboxylase (EC 4.1.1.15)	1	6
CADAFUBP00006867	AFUB_070500	B0Y4W1	Gamma-glutamyltranspeptidase	1	6
CADAFUBP00006821	AFUB_070010	B0Y4R5	Glutamine synthetase (EC 6.3.1.2)	1	6
CADAFUBP00003724	AFUB_038020	B0XXN4	Glutamate decarboxylase, putative	1	6
CADAFUBP00003455	AFUB_035300	B0Y0A3	Glutamate-cysteine ligase Gcs1, putative	1	6
CADAFUBP00003419	AFUB_034950	B0Y067	Gamma-cysteine synthetase regulatory subunit, putative	1	6
CADAFUBP00003043	AFUB_031050	B0XUI9	FluG family protein	1	6
CADAFUBP00000761	AFUB_007730	B0XPV7	Glutamate synthase Glt1, putative	1	6
CADAFUBP00000123	AFUB_001260	B0XM77	Glutamate decarboxylase (EC 4.1.1.15)	1	6
CADAFUBP00000042	AFUB_000420	B0XMB3	5-oxo-L-prolinase, putative	1	6
CADAFUBP00009314	AFUB_095850	B0YDJ9	Aspartate aminotransferase (EC 2.6.1.1)	1	6
CADAFUBP00007278	AFUB_074710	B0Y7R3	Delta-1-pyrroline-5-carboxylate dehydrogenase PrnC	1	6
CADAFUBP00006570	AFUB_067510	B0Y6M8	Aspartate aminotransferase, putative	1	6
CADAFUBP00006223	AFUB_063970	B0Y5N1	Asparagine synthetase Asn2, putative	1	6
CADAFUBP00006196	AFUB_063700	B0Y5K4	Glutamate dehydrogenase	1	6
CADAFUBP00005448	AFUB_055650	B0Y3W4	Arginine biosynthesis bifunctional protein ArgJ, mitochondrial [Cleaved into: Arginine biosynthesis bifunctional protein ArgJ alpha chain; Arginine biosynthesis bifunctional protein ArgJ beta chain] [Includes: Glutamate N-acetyltransferase (GAT) (EC 2.3.1.35) (Ornithine acetyltransferase) (OATase) (Ornithine transacetylase); Amino-acid acetyltransferase (EC 2.3.1.1) (N-acetylglutamate synthase) (AGS)]	1	6
CADAFUBP00002663	AFUB_027240	B0XSH8	Amino-acid acetyltransferase, mitochondrial (EC 2.3.1.1) (Arginine-requiring protein 2) (Glutamate N-acetyltransferase) (N-acetylglutamate synthase) (AGS) (NAGS)	1	6
CADAFUBP00002493	AFUB_025510	B0XS08	Aspartate transaminase, putative	1	6
CADAFUBP00002305	AFUB_023620	B0XW54	Glutamate 5-kinase, putative	1	6
CADAFUBP00002251	AFUB_023080	B0XVW9	NAD-specific glutamate dehydrogenase (EC 1.4.1.2)	1	6
CADAFUBP00002086	AFUB_021370	B0XV21	Argininosuccinate synthase	1	6
CADAFUBP00002083	AFUB_021340	B0XV18	Asparaginase, putative	1	6
CADAFUBP00001470	AFUB_015000	B0XNF8	Adenylosuccinate synthetase (AMPSase) (AdSS) (EC 6.3.4.4) (IMP--aspartate ligase)	1	6
CADAFUBP00000445	AFUB_004510	B0XND0	Aspartate aminotransferase, putative	1	6
CADAFUBP00000310	AFUB_003170	B0XML5	L-asparaginase	1	6
CADAFUBP00008584	AFUB_088300	B0YBY9	3-hydroxymethyl-3-methylglutaryl-Coenzyme A lyase	1	6
CADAFUBP00007628	AFUB_078260	B0Y8R3	Succinyl-CoA:3-ketoacid-coenzyme A transferase (EC 2.8.3.5)	1	6
CADAFUBP00002750	AFUB_028110	B0XSX3	Hydroxymethylglutaryl-CoA lyase	1	6
CADAFUBP00000441	AFUB_004480	B0XNC6	Succinyl-CoA:3-ketoacid-coenzyme A transferase (EC 2.8.3.5)	1	6
CADAFUBP00008876	AFUB_091290	B0YB22	Acetyltransferase component of pyruvate dehydrogenase complex (EC 2.3.1.12)	1	6
CADAFUBP00005670	AFUB_057960	B0Y0N6	Succinate dehydrogenase [ubiquinone] iron-sulfur subunit, mitochondrial (EC 1.3.5.1)	1	6
CADAFUBP00005605	AFUB_057230	B0Y4C1	Succinate dehydrogenase cytochrome b560 subunit	1	6
CADAFUBP00004046	AFUB_041300	B0XYL2	Succinate dehydrogenase [ubiquinone] flavoprotein subunit, mitochondrial (EC 1.3.5.1)	1	6
CADAFUBP00001871	AFUB_019180	B0XTV0	Dihydrolipoyl dehydrogenase (EC 1.8.1.4)	1	6
CADAFUBP00001006	AFUB_010250	B0XQK2	Succinyl-CoA synthetase, alpha subunit, putative	1	6
CADAFUBP00007537	AFUB_077330	B0Y8H2	Bifunctional pyrimidine biosynthesis protein	1	6

			(PyrABCN), putative		
CADAFUBP00000693	AFUB_007050	B0XPN9	CBS domain protein	1	6
CADAFUBP00009204	AFUB_094700	B0YD89	2-methylcitrate synthase, mitochondrial (Methylcitrate synthase) (EC 2.3.3.5) ((2S,3S)-2-methylcitrate synthase) (Citrate synthase 2) (EC 2.3.3.16)	1	6
CADAFUBP00009043	AFUB_093060	B0YCS8	Malate dehydrogenase, NAD-dependent	1	6
CADAFUBP00008879	AFUB_091320	B0YB25	Malate dehydrogenase (EC 1.1.1.37)	1	6
CADAFUBP00007175	AFUB_073680	B0Y7G0	Phosphoenolpyruvate carboxykinase AcuF	1	6
CADAFUBP00006306	AFUB_064810	B0Y5W4	Pyruvate carboxylase (EC 6.4.1.1)	1	6
CADAFUBP00005164	AFUB_052750	B0Y330	Citrate synthase	1	6
CADAFUBP00004048	AFUB_041320	B0XYL4	Argininosuccinate lyase	1	6
CADAFUBP00003035	AFUB_030970	B0XUI1	Citrate synthase	1	6
CADAFUBP00002705	AFUB_027670	B0XSM0	Adenylosuccinate lyase (ASL) (EC 4.3.2.2) (Adenylosuccinase)	1	6
CADAFUBP00006327	AFUB_065020	B0Y5Y5	Pitrilysin family metalloprotease (Cym1), putative	1	7
CADAFUBP00005195	AFUB_053060	B0Y361	D-3-phosphoglycerate dehydrogenase	1	7
CADAFUBP00002101	AFUB_021520	B0XV36	D-3-phosphoglycerate dehydrogenase (EC 1.1.1.95)	1	7
CADAFUBP00009668	AFUB_099480	B0YEK3	Uncharacterized protein	1	7
CADAFUBP00007006	AFUB_071890	B0Y5A0	Ribokinase (RK) (EC 2.7.1.15)	1	7
CADAFUBP00004997	AFUB_051060	B0Y2D4	Ribokinase	1	7
CADAFUBP00009301	AFUB_095710	B0YDI6	Phosphoglycerate mutase family protein	1	7
CADAFUBP00007253	AFUB_074460	B0Y7N8	Phosphoglycerate mutase family protein	1	7
CADAFUBP00000958	AFUB_009760	B0XQF4	Phosphoglycerate kinase (EC 2.7.2.3)	1	7
CADAFUBP00005974	AFUB_061150	B0Y239	Triosephosphate isomerase (EC 5.3.1.1)	1	7
CADAFUBP00003902	AFUB_039830	B0XY68	Phosphoglycerate mutase, 2,3-bisphosphoglycerate-independent	1	7
CADAFUBP00002619	AFUB_026790	B0XSD4	Triosephosphate isomerase (EC 5.3.1.1)	1	7
CADAFUBP00008952	AFUB_092050	B0YB98	Deoxyribose-phosphate aldolase	1	7
CADAFUBP00004163	AFUB_042460	B0XYX9	Deoxyribose-phosphate aldolase	1	7
CADAFUBP00006423	AFUB_065980	B0Y681	Possible apospory-associated protein c	1	7
CADAFUBP00004655	AFUB_047490	B0XX26	Alpha-1,3-glucan synthase, putative	1	7
CADAFUBP00003657	AFUB_037350	B0XXA2	Phosphoglucomutase PgmA	1	7
CADAFUBP00002642	AFUB_027030	B0XSF7	Alpha-1,3-glucan synthase, putative	1	7
CADAFUBP00001469	AFUB_014990	B0XNF7	Alpha-1,3-glucan synthase, putative	1	7
CADAFUBP00009713	AFUB_099940	B0YEP8	Alpha,alpha-trehalose-phosphate synthase subunit, putative	1	7
CADAFUBP00008697	AFUB_089470	B0YCA2	Alpha,alpha-trehalose phosphate synthase subunit TPS3, putative	1	7
CADAFUBP00006056	AFUB_062010	B0Y2J2	Alpha,alpha-trehalose-phosphate synthase subunit, putative	1	7
CADAFUBP00004253	AFUB_043350	B0XZ69	Alpha,alpha-trehalose-phosphate synthase subunit TPS2, putative	1	7
CADAFUBP00002058	AFUB_021090	B0XUS4	Alpha,alpha-trehalose phosphate synthase subunit, putative	1	7
CADAFUBP00002057	AFUB_021080	B0XUS3	Trehalose-6-phosphate synthase (EC 2.4.1.15) (UDP-glucose-glucosephosphate glucosyltransferase)	1	7
CADAFUBP00000174	AFUB_001790	B0XM02	Trehalose-6-phosphate synthase (EC 2.4.1.15) (UDP-glucose-glucosephosphate glucosyltransferase)	1	7
CADAFUBP00007593	AFUB_077900	B0Y8M8	Probable beta-glucosidase J (EC 3.2.1.21) (Beta-D-glucoside glucohydrolase J) (Cellobiase J) (Gentiobiase J)	1	7
CADAFUBP00009206	AFUB_094720	B0YD91	Probable beta-glucosidase E (EC 3.2.1.21) (Beta-D-glucoside glucohydrolase E) (Cellobiase E) (Gentiobiase E)	1	7
CADAFUBP00004723	AFUB_048210	B0XXG1	Beta-glucosidase (EC 3.2.1.21)	1	7
CADAFUBP00009853	AFUB_101460	B0YF38	ATP-dependent 6-phosphofructokinase (ATP-PFK) (Phosphofructokinase) (EC 2.7.1.11) (Phosphohexokinase)	1	7
CADAFUBP00009799	AFUB_100920	B0YEY4	Glycosyl hydrolase, putative	1	7
CADAFUBP00001873	AFUB_019200	B0XTV2	Phosphoglucomutase, putative	1	7
CADAFUBP00007273	AFUB_074660	B0Y7Q8	Probable beta-glucosidase F (EC 3.2.1.21) (Beta-D-glucoside glucohydrolase F) (Cellobiase F)	1	7

			(Gentiobiase F)		
CADAFUBP00007059	AFUB_072510	B0Y744	Phosphomannomutase (EC 5.4.2.8)	1	7
CADAFUBP00006377	AFUB_065520	B0Y635	Mannose-6-phosphate isomerase, class I	1	7
CADAFUBP00004435	AFUB_045220	B0Y009	Phosphoglucomutase, putative	1	7
CADAFUBP00001256	AFUB_012770	B0XRB5	Mannose-6-phosphate isomerase (EC 5.3.1.8)	1	7
CADAFUBP00001543	AFUB_015740	B0XNU9	Beta-glucosidase, putative	1	7
CADAFUBP00001646	AFUB_016780	B0XPB8	Probable beta-glucosidase M (EC 3.2.1.21) (Beta-D-glucoside glucohydrolase M) (Cellobiase M) (Gentiobiase M)	1	7
CADAFUBP00005360	AFUB_054750	B0Y3M6	Probable beta-glucosidase I (EC 3.2.1.21) (Beta-D-glucoside glucohydrolase I) (Cellobiase I) (Gentiobiase I)	1	7
CADAFUBP00001397	AFUB_014260	B0XMV0	Beta-glucosidase, putative	1	7
CADAFUBP00005349	AFUB_054640	B0Y3L5	Beta-glucosidase, putative	1	7
CADAFUBP00000017	AFUB_000170	B0XMG5	Beta-glucosidase, putative	1	7
CADAFUBP00007602	AFUB_077990	B0Y8N7	Glycosyl hydrolase family protein	1	7
CADAFUBP00008126	AFUB_083490	B0YA57	Glucosamine-6-phosphate isomerase (EC 3.5.99.6) (Glucosamine-6-phosphate deaminase)	1	7
CADAFUBP00007078	AFUB_072700	B0Y763	Enolase/allergen Asp F 22	1	7
CADAFUBP00007148	AFUB_073410	B0Y7D3	Pyruvate kinase (EC 2.7.1.40)	1	7
CADAFUBP00006654	AFUB_068340	B0Y6W2	Fructose-1,6-bisphosphatase	1	7
CADAFUBP00007319	AFUB_075140	B0Y7V4	Ubiquitin-like protein ATG12	1	7
CADAFUBP00001983	AFUB_020300	B0XUD0	Alpha-amylase AmyA	1	7
CADAFUBP00001371	AFUB_013990	B0XMS4	Exo-beta-1,3-glucanase Exg0	1	7
CADAFUBP00000145	AFUB_001500	B0XLY8	Exo-beta-1,3-glucanase, putative	1	7
CADAFUBP00007327	AFUB_075220	B0Y7W2	Probable glucan 1,3-beta-glucosidase D (EC 3.2.1.58) (Exo-1,3-beta-glucanase D)	1	7
CADAFUBP00005898	AFUB_060350	B0Y1H5	Dihydroxyacetone kinase (DakA), putative	1	7
CADAFUBP00003024	AFUB_030860	B0XUH0	Ribulose-phosphate 3-epimerase	1	7
CADAFUBP00007033	AFUB_072250	B0Y718	Glucosamine-fructose-6-phosphate aminotransferase	1	7
CADAFUBP00001706	AFUB_017490	B0XST0	Exo-beta-1,3-glucanase, putative	1	7
CADAFUBP00002137	AFUB_021900	B0XVD8	Pyruvate dehydrogenase E1 component alpha subunit, putative	1	7
CADAFUBP00000722	AFUB_007340	B0XPR8	Pyruvate dehydrogenase E1 component subunit alpha (EC 1.2.4.1)	1	7
CADAFUBP00000394	AFUB_004010	B0XN12	Probable glucan 1,3-beta-glucosidase A (EC 3.2.1.58) (Exo-1,3-beta-glucanase 1) (Exo-1,3-beta-glucanase A)	1	7
CADAFUBP00004076	AFUB_041600	B0XYP2	Exo-beta-1,3-glucanase, putative	1	7
CADAFUBP00008941	AFUB_091940	B0YB87	Maltase	1	7
CADAFUBP00005688	AFUB_058160	B0Y0Q4	1,4-alpha-glucan branching enzyme	1	7
CADAFUBP00001219	AFUB_012400	B0XR78	Alpha-1,4 glucan phosphorylase (EC 2.4.1.1)	1	7
CADAFUBP00008179	AFUB_084020	B0YAB0	Glyceraldehyde-3-phosphate dehydrogenase, putative	1	7
CADAFUBP00005662	AFUB_057870	B0Y0M8	Fructose-bisphosphate aldolase, putative	1	7
CADAFUBP00005559	AFUB_056770	B0Y475	Transaldolase (EC 2.2.1.2)	1	7
CADAFUBP00004940	AFUB_050490	B0Y207	Glyceraldehyde-3-phosphate dehydrogenase (EC 1.2.1.12)	1	7
CADAFUBP00004842	AFUB_049500	B0Y1J3	Glyceraldehyde-3-phosphate dehydrogenase (EC 1.2.1.12)	1	7
CADAFUBP00003672	AFUB_037500	B0XXB7	Fructose-bisphosphate aldolase, class II	1	7
CADAFUBP00002505	AFUB_025630	B0XS20	Glucose-6-phosphate isomerase (EC 5.3.1.9)	1	7
CADAFUBP00004447	AFUB_045340	B0Y021	Dihydroxy-acetone synthase, putative	1	7
CADAFUBP00003978	AFUB_040620	B0XYE4	Glucose-6-phosphate 1-dehydrogenase (EC 1.1.1.49)	1	7
CADAFUBP00001278	AFUB_012990	B0XRK4	Transketolase (EC 2.2.1.1)	1	7
CADAFUBP00000329	AFUB_003360	B0XMN4	6-phosphogluconolactonase, putative	1	7
CADAFUBP00006544	AFUB_067250	B0Y6K2	Alpha-amylase (EC 3.2.1.1)	1	7
CADAFUBP00002850	AFUB_029100	B0XTE2	Alpha-amylase, putative	1	7
CADAFUBP00001735	AFUB_017790	B0XT27	Alpha-amylase, putative	1	7
CADAFUBP00001441	AFUB_014700	B0XN61	Alpha-amylase, putative	1	7
CADAFUBP00009317	AFUB_095880	B0YDK2	Glucosamine 6-phosphate acetyltransferase, putative	1	7
CADAFUBP00008123	AFUB_083460	B0YA54	N-acetylglucosamine-6-phosphate deacetylase (EC	1	7

			3.5.1.25)		
CADAFUBP00001653	AFUB_016850	B0XPC5	Glucokinase regulator family protein, putative	1	7
CADAFUBP00000647	AFUB_006590	B0XPI4	N-acetylglucosamine-phosphate mutase	1	7
CADAFUBP00004686	AFUB_047820	B0XX57	Glucoamylase (EC 3.2.1.3) (1,4-alpha-D-glucan glucohydrolase) (Glucan 1,4-alpha-glucosidase)	1	7
CADAFUBP00001733	AFUB_017770	B0XSV7	Glucoamylase (EC 3.2.1.3) (1,4-alpha-D-glucan glucohydrolase) (Glucan 1,4-alpha-glucosidase)	1	7
CADAFUBP00000247	AFUB_002520	B0XRH8	Glycogen debranching enzyme Gdb1, putative	1	7
CADAFUBP00008919	AFUB_091720	B0YB65	Probable beta-glucosidase L (EC 3.2.1.21) (Beta-D-glucoside glucohydrolase L) (Cellobiase L) (Gentiobiase L)	1	7
CADAFUBP00001069	AFUB_010890	B0XQR5	1,3-beta-glucanosyltransferase Bgt1	1	7
CADAFUBP00009066	AFUB_093290	B0YCV1	Glycosyl hydrolases family 32 superfamily	1	7
CADAFUBP00007862	AFUB_080730	B0Y9E3	Maltase MalT	1	7
CADAFUBP00007599	AFUB_077960	B0Y8N4	Exo-beta-1,3-glucanase, putative	1	7
CADAFUBP00006546	AFUB_067270	B0Y6K4	Alpha-glucosidase AgdA, putative	1	7
CADAFUBP00004785	AFUB_048930	B0Y174	Exoinulinase InuD	1	7
CADAFUBP00001787	AFUB_018320	B0XT79	Beta-fructofuranosidase, putative	1	7
CADAFUBP00001528	AFUB_015590	B0XNL6	Probable alpha/beta-glucosidase agdC (EC 3.2.1.20) (EC 3.2.1.21)	1	7
CADAFUBP00000604	AFUB_006160	B0XPE1	Probable beta-glucosidase A (EC 3.2.1.21) (Beta-D-glucoside glucohydrolase A) (Cellobiase A) (Gentiobiase A)	1	7
CADAFUBP00009865	AFUB_101570	B0YF50	Acid phosphatase, putative	1	7
CADAFUBP00009338	AFUB_096090	B0YDM3	Phosphotransferase (EC 2.7.1.-)	1	7
CADAFUBP00009167	AFUB_094300	B0YD52	Phosphotransferase (EC 2.7.1.-)	1	7
CADAFUBP00008707	AFUB_089570	B0YCB2	Phosphotransferase (EC 2.7.1.-)	1	7
CADAFUBP00008236	AFUB_084610	B0YAG7	Sorbitol/xylitol dehydrogenase, putative	1	7
CADAFUBP00006987	AFUB_071700	B0Y581	Mannitol 2-dehydrogenase (M2DH) (MDH) (EC 1.1.1.67)	1	7
CADAFUBP00005831	AFUB_059590	B0Y147	Serine/threonine-protein phosphatase (EC 3.1.3.16)	1	7
CADAFUBP00004318	AFUB_044020	B0XZD4	2,3-diketo-5-methylthio-1-phosphopentane phosphatase, putative	1	7
CADAFUBP00003813	AFUB_038940	B0XXX9	Acid phosphatase, putative	1	7
CADAFUBP00003137	AFUB_032000	B0XUZ8	Phosphotransferase (EC 2.7.1.-)	1	7
CADAFUBP00002241	AFUB_022950	B0XVV9	Phosphotransferase (EC 2.7.1.-)	1	7
CADAFUBP00001708	AFUB_017510	B0XST2	Phosphotransferase (EC 2.7.1.-)	1	7
CADAFUBP00001530	AFUB_015610	B0XNT6	Xylitol dehydrogenase XdhB, putative	1	7
CADAFUBP00001365	AFUB_013930	B0XMR8	Zinc-dependent alcohol dehydrogenase, putative (EC 1.1.1.-) (Fragment)	1	7
CADAFUBP00001026	AFUB_010450	B0XQM2	Xylitol dehydrogenase	1	7
CADAFUBP00001025	AFUB_010440	B0XQM1	L-arabinitol 4-dehydrogenase	1	7
CADAFUBP00000400	AFUB_004070	B0XN85	Acid phosphatase, putative	1	7
CADAFUBP00003632	AFUB_037100	B0XX77	ADP-ribosylation factor 6, putative	1	8
CADAFUBP00008863	AFUB_091160	B0YB09	Phospholipase PldA, putative	1	8
CADAFUBP00004255	AFUB_043370	B0XZ71	Phospholipase D1 (PLD1), putative	1	8
CADAFUBP00003156	AFUB_032200	B0XV82	Phospholipase D (PLD), putative	1	8
CADAFUBP00006439	AFUB_066150	B0Y697	Serine/threonine protein kinase (Pdd7p), putative	1	8
CADAFUBP00001914	AFUB_019610	B0XTZ3	Meiotic regulator-interacting protein, putative	1	8
CADAFUBP00009074	AFUB_093370	B0YCV9	Formate dehydrogenase (FDH) (EC 1.2.1.2) (NAD-dependent formate dehydrogenase)	1	9
CADAFUBP00007420	AFUB_076160	B0Y855	D-isomer specific 2-hydroxyacid dehydrogenase family protein	1	9
CADAFUBP00006860	AFUB_070430	B0Y4V4	Isocitrate lyase	1	9
CADAFUBP00001291	AFUB_013120	B0XRL7	Glycerate dehydrogenase	1	9
CADAFUBP00009875	AFUB_101660	B0YF60	N,N-dimethylglycine oxidase	1	9
CADAFUBP00009646	AFUB_099260	B0YEI1	Glycine dehydrogenase	1	9
CADAFUBP00008959	AFUB_092120	B0YBA5	Threonine aldolase, putative	1	9
CADAFUBP00007433	AFUB_076290	B0Y868	FAD dependent oxidoreductase superfamily	1	9
CADAFUBP00006021	AFUB_061650	B0Y286	FAD dependent oxidoreductase superfamily	1	9
CADAFUBP00005758	AFUB_058860	B0Y0X4	D-amino acid oxidase	1	9

CADAFUBP00005267	AFUB_053800	B0Y3D3	5-aminolevulinate synthase (EC 2.3.1.37) (5-aminolevulinic acid synthase) (Delta-ALA synthase) (Delta-aminolevulinate synthase)	1	9
CADAFUBP00004628	AFUB_047220	B0XWZ9	Sarcosine oxidase, putative	1	9
CADAFUBP00003900	AFUB_039810	B0XY66	Serine hydroxymethyltransferase (EC 2.1.2.1)	1	9
CADAFUBP00003165	AFUB_032290	B0XV91	N,N-dimethylglycine oxidase	1	9
CADAFUBP00002996	AFUB_030580	B0XU74	Alanine racemase	1	9
CADAFUBP00002327	AFUB_023840	B0XW76	Serine hydroxymethyltransferase (EC 2.1.2.1)	1	9
CADAFUBP00000873	AFUB_008920	B0XQ69	Aminotransferase, class V, putative	1	9
CADAFUBP00008206	NA	NA	NA	1	9
CADAFUBP00009209	AFUB_094750	B0YD94	Malate synthase (EC 2.3.3.9)	1	9
CADAFUBP00007678	AFUB_078770	B0Y8W3	Proteasome regulatory particle subunit (RpnI), putative	1	10
CADAFUBP00007079	AFUB_072710	B0Y764	Proteasome regulatory particle subunit Rpt4, putative	1	10
CADAFUBP00004045	AFUB_041290	B0XYL1	Proteasome regulatory particle subunit Rpt1, putative	1	10
CADAFUBP00000643	AFUB_006550	B0XPI0	Proteasome regulatory particle subunit Rpt5, putative	1	10
CADAFUBP00001446	AFUB_014750	B0XN66	Hsp70 family protein	1	10
CADAFUBP00007060	AFUB_072520	B0Y745	DnaJ chaperone (Caj1), putative (Fragment)	1	10
CADAFUBP00001894	AFUB_019410	B0XTX3	Hsp70 chaperone (BiP), putative	1	10
CADAFUBP00002370	AFUB_024270	B0XWB9	DnaJ domain protein, putative	1	10
CADAFUBP00001041	AFUB_010610	B0XQN7	Heat shock protein/chaperonin HSP78, putative	1	10
CADAFUBP00009657	AFUB_099370	B0YEJ2	Ribosome associated DnaJ chaperone Zuotin, putative	1	10
CADAFUBP00004198	AFUB_042810	B0XZ14	Translation initiation factor SUI1, putative	1	10
CADAFUBP00009311	AFUB_095820	B0YDJ6	Eukaryotic translation initiation factor eIF-1A subunit, putative	1	10
CADAFUBP00000954	AFUB_009720	B0XQF0	RNase L inhibitor of the ABC superfamily, putative	1	10
CADAFUBP00000430	AFUB_004370	B0XNB5	Mitochondrial translation initiation factor IF-2, putative	1	10
CADAFUBP00008835	AFUB_090870	B0YCP0	40S ribosomal protein S13	1	10
CADAFUBP00002069	AFUB_021200	B0XUT5	40S ribosomal protein S11	1	10
CADAFUBP00000460	AFUB_004660	B0XNE5	40S ribosomal protein S8	1	10
CADAFUBP00006267	AFUB_064420	B0Y5S5	ER associated DnaJ chaperone (Hlj1), putative	1	10
CADAFUBP00005600	AFUB_057180	B0Y4B6	UBA/TS-N domain protein	1	10
CADAFUBP00000904	AFUB_009220	B0XQA0	La domain family	1	10
CADAFUBP00001453	AFUB_014820	B0XN73	Heat shock protein Hsp98/Hsp104/ClpA, putative	1	10
CADAFUBP00008163	AFUB_083860	B0YA94	Translation machinery-associated protein 22	1	10
CADAFUBP00002431	AFUB_024880	B0XRU6	Eukaryotic translation initiation factor 5, putative	1	10
CADAFUBP00006292	AFUB_064670	B0Y5V0	Translation initiation factor EF-2 gamma subunit, putative	1	10
CADAFUBP00003966	AFUB_040500	B0XYD2	Translational initiation factor 2 beta	1	10
CADAFUBP00003495	AFUB_035720	B0XWF4	Translation initiation factor 2 alpha subunit, putative	1	10
CADAFUBP00002116	AFUB_021670	B0XV51	ER Hsp70 chaperone BiP, putative	1	10
CADAFUBP00000617	AFUB_006290	B0XPF4	DnaJ and TPR domain protein	1	10
CADAFUBP00004226	AFUB_043080	B0XZ42	Eukaryotic translation initiation factor subunit eIF2A, putative	1	10
CADAFUBP00006656	AFUB_068360	B0Y6W4	Aha1 domain family	1	10
CADAFUBP00006019	AFUB_061630	B0Y284	Hsp90 binding co-chaperone (Sba1), putative	1	10
CADAFUBP00001866	AFUB_019130	B0XTM6	Peptidyl-prolyl cis-trans isomerase Cpr7, putative	1	10
CADAFUBP00006531	AFUB_067120	B0Y6I9	Hsp90 co-chaperone Cdc37	1	10
CADAFUBP00003755	AFUB_038330	B0XXS1	Clustered mitochondria protein homolog (Protein TIF31 homolog)	1	10
CADAFUBP00009636	AFUB_099160	B0YEH1	Eukaryotic translation initiation factor 3 subunit C (eIF3c) (Eukaryotic translation initiation factor 3 93 kDa subunit homolog) (eIF3 p93) (Translation initiation factor eIF3, p93 subunit homolog)	1	10
CADAFUBP00008701	AFUB_089510	B0YCA6	Eukaryotic translation initiation factor 3 subunit M (eIF3m)	1	10
CADAFUBP00003962	AFUB_040450	B0XYC8	Eukaryotic translation initiation factor 3 subunit I (eIF3i) (Eukaryotic translation initiation factor 3 39 kDa subunit homolog) (eIF-3 39 kDa subunit homolog)	1	10

CADAFUBP00003903	AFUB_039840	B0XY69	Eukaryotic translation initiation factor 3 subunit K (eIF3k) (eIF-3 p25)	1	10
CADAFUBP00003703	AFUB_037810	B0XXL3	Eukaryotic translation initiation factor 3 subunit E (eIF3e)	1	10
CADAFUBP00002969	AFUB_030310	B0XU47	Eukaryotic translation initiation factor 3 subunit D (eIF3d)	1	10
CADAFUBP00002559	AFUB_026190	B0XS74	Eukaryotic translation initiation factor 3 subunit L (eIF3l)	1	10
CADAFUBP00002513	AFUB_025710	B0XS28	Eukaryotic translation initiation factor 3 subunit G (eIF3g) (Eukaryotic translation initiation factor 3 RNA-binding subunit) (eIF-3 RNA-binding subunit) (Translation initiation factor eIF3 p33 subunit homolog) (eIF3 p33 homolog)	1	10
CADAFUBP00000923	AFUB_009410	B0XQB9	Eukaryotic translation initiation factor 3 subunit H (eIF3h)	1	10
CADAFUBP00000236	AFUB_002410	B0XRG7	Eukaryotic translation initiation factor 3 subunit B (eIF3b) (Eukaryotic translation initiation factor 3 90 kDa subunit homolog) (eIF3 p90) (Translation initiation factor eIF3 p90 subunit homolog)	1	10
CADAFUBP00008559	AFUB_088050	B0YBW4	Cap binding protein	1	10
CADAFUBP00004010	AFUB_040950	B0XYH6	Eukaryotic translation initiation factor 4, putative	1	10
CADAFUBP00002478	AFUB_025360	B0XRZ3	Eukaryotic translation initiation factor subunit eIF-4F, putative	1	10
CADAFUBP00000546	AFUB_005560	B0XP13	Eukaryotic translation initiation factor 3 subunit A (eIF3a) (Eukaryotic translation initiation factor 3 110 kDa subunit homolog) (eIF3 p110) (Translation initiation factor eIF3, p110 subunit homolog)	1	10
CADAFUBP00000447	AFUB_004530	B0XND2	Polyadenylate-binding protein (PABP)	1	10
CADAFUBP00008597	AFUB_088430	B0YC02	Heat shock protein (Sti1), putative	1	10
CADAFUBP00008141	AFUB_083640	B0YA72	Hsp70 chaperone (HscA), putative	1	10
CADAFUBP00003133	AFUB_031960	B0XUZ4	DnaJ domain protein (Mas5), putative	1	10
CADAFUBP00002521	AFUB_025800	B0XS36	Mitochondrial Hsp70 chaperone (Ssc70), putative	1	10
CADAFUBP00000765	AFUB_007770	B0XPW1	Molecular chaperone Hsp70	1	10
CADAFUBP00000531	AFUB_005400	B0XNT1	Protein mitochondrial targeting protein (Mas1), putative	1	10
CADAFUBP00000588	AFUB_006000	B0XP55	40S ribosomal protein S3, putative	1	10
CADAFUBP00001318	AFUB_013400	B0XRP4	TFIIH complex helicase Rad3, putative	1	10
CADAFUBP00009093	AFUB_093560	B0YCX8	Bifunctional purine biosynthetic protein Ade1, putative	1	10
CADAFUBP00001223	AFUB_012440	B0XR82	SCF ubiquitin ligase complex subunit CulA, putative	1	10
CADAFUBP00002000	AFUB_020470	B0XUE7	Proteasome regulatory particle subunit (RpnK), putative	1	10
CADAFUBP00007225	AFUB_074180	B0Y7L0	SCF ubiquitin ligase subunit CulC, putative	1	10
CADAFUBP00004636	AFUB_047300	B0XX07	GMP synthase	1	10
CADAFUBP00002193	AFUB_022460	B0XVJ4	Uridine kinase (EC 2.7.1.48)	1	10
CADAFUBP00004857	AFUB_049650	B0Y1K8	RAN small monomeric GTPase (Ran), putative	1	10
CADAFUBP00000142	AFUB_001470	B0XME8	GTP-binding nuclear protein	1	10
CADAFUBP00009060	AFUB_093230	B0YCU5	DUF410 domain protein	1	10
CADAFUBP00003705	AFUB_037830	B0XXL5	ATPase get3 (EC 3.6.-.-) (Arsenical pump-driving ATPase) (Arsenite-stimulated ATPase) (Golgi to ER traffic protein 3) (Guided entry of tail-anchored proteins 3)	1	10
CADAFUBP00002848	AFUB_029080	B0XTE0	Chitin synthase ChsE	1	10
CADAFUBP00008910	AFUB_091630	B0YB56	DNA damage-inducible v-SNARE binding protein Ddi1, putative	1	10
CADAFUBP00005802	AFUB_059290	B0Y118	26S proteasome regulatory subunit Mts4, putative	1	10
CADAFUBP00006216	AFUB_063900	B0Y5M4	SUMO-conjugating enzyme (EC 2.3.2.-)	1	10
CADAFUBP00005849	AFUB_059770	B0Y1C6	Ran-specific GTPase-activating protein 1, putative	1	10
CADAFUBP00004058	AFUB_041420	B0XYM4	Ran GTPase activating protein 1 (RNA1 protein)	1	10
CADAFUBP00005681	AFUB_058070	B0Y0P7	Adenylyltransferase and sulfurtransferase uba4 (Common component for nitrate reductase and xanthine dehydrogenase protein F) (Ubiquitin-like protein activator 4) [Includes: Molybdopterin-synthase adenylyltransferase (EC 2.7.7.80) (Adenylyltransferase uba4) (Sulfur carrier protein	1	10

			MOCS2A adenylyltransferase); Molybdopterin-synthase sulfurtransferase (EC 2.8.1.11) (Sulfur carrier protein MOCS2A sulfurtransferase) (Sulfurtransferase uba4)]		
CADAFUBP00001008	AFUB_010270	B0XQK4	Ubiquitin-like modifier SUMO, putative	1	10
CADAFUBP00005016	AFUB_051250	B0Y2F3	Phosphoribosylformylglycinamidine synthase	1	10
CADAFUBP00002805	AFUB_028660	B0XT97	Phenylalanyl-tRNA synthetase alpha subunit (PodG), putative	1	10
CADAFUBP00008549	AFUB_087950	B0YBV4	Anaphase promoting complex subunit Apc11, putative	1	10
CADAFUBP00005556	AFUB_056740	B0Y472	Ubiquitin conjugating enzyme (UbcC), putative	1	10
CADAFUBP00005249	AFUB_053620	B0Y3B5	E3 ubiquitin ligase complex SCF subunit sconC (Sulfur controller C) (Sulfur metabolite repression control protein C)	1	10
CADAFUBP00004204	AFUB_042860	B0XZ20	Anaphase-promoting complex subunit Apc5, putative	1	10
CADAFUBP00008897	AFUB_091500	B0YB43	Acyl-CoA desaturase (EC 1.14.19.1)	1	10
CADAFUBP00006268	AFUB_064430	B0Y5S6	Ubiquitin fusion degradation protein UfdB, putative	1	10
CADAFUBP00003010	AFUB_030720	B0XUF6	26S proteasome regulatory subunit S5A	1	10
CADAFUBP00000910	AFUB_009280	B0XQA6	Hsc70 cochaperone (SGT), putative	1	10
CADAFUBP00003880	AFUB_039610	B0XY46	DNA polymerase epsilon, catalytic subunit A/POL2, putative	1	10
CADAFUBP00002270	AFUB_023270	B0XW19	DNA polymerase epsilon subunit B, putative	1	10
CADAFUBP00000978	AFUB_009970	B0XQH4	CBF/NF-Y family transcription factor, putative	1	10
CADAFUBP00007255	AFUB_074480	B0Y7P0	Adenylate cyclase AcyA	1	10
CADAFUBP00001236	AFUB_012570	B0XR95	Multifunctional tryptophan biosynthesis protein	1	10
CADAFUBP00002309	AFUB_023660	B0XW58	Cystathionine beta-synthase (EC 4.2.1.22)	1	10
CADAFUBP00005158	AFUB_052690	B0Y324	Molecular chaperone Mod-E/Hsp90	1	10
CADAFUBP00000859	AFUB_008750	B0XQ55	Eukaryotic translation initiation factor 3 subunit F (eIF3f)	1	10
CADAFUBP00002018	AFUB_020690	B0XUN4	Inosine-5'-monophosphate dehydrogenase (IMP dehydrogenase) (IMPD) (IMPDH) (EC 1.1.1.205)	1	10
CADAFUBP00004992	AFUB_051010	B0Y2C9	Glycogen [starch] synthase (EC 2.4.1.11)	1	10
CADAFUBP00006607	AFUB_067880	B0Y6R5	40S ribosomal protein S6	1	10
CADAFUBP00001004	AFUB_010230	B0XQK0	V-type proton ATPase subunit C	1	10
CADAFUBP00004660	NA	NA	NA	1	10
CADAFUBP00003713	AFUB_037910	B0XXM3	Ubiquitin (UbiC), putative	1	10
CADAFUBP00002806	AFUB_028670	B0XT98	GrpE protein homolog	1	10
CADAFUBP00005247	AFUB_053580	B0Y3B3	UV excision repair protein (RadW), putative	1	10
CADAFUBP00000130	AFUB_001330	B0XMD6	Ubiquitin-like protein DskB, putative	1	10
CADAFUBP00007985	AFUB_082020	B0Y9R6	Fumarate reductase Osm1, putative	1	10
CADAFUBP00002644	AFUB_027050	B0XSF9	Orotate phosphoribosyltransferase	1	10
CADAFUBP00002618	AFUB_026780	B0XSD3	Dihydroorotate reductase PyrE, putative	1	10
CADAFUBP00002374	AFUB_024310	B0XWC3	Orotidine 5'-phosphate decarboxylase (EC 4.1.1.23)	1	10
CADAFUBP00001886	AFUB_019330	B0XTW5	Allantoinase Dal1, putative	1	10
CADAFUBP00008688	AFUB_089380	B0YC93	Oxidoreductase, 2-nitropropane dioxygenase family, putative	1	10
CADAFUBP00005675	AFUB_058010	B0Y0P1	Nitrate reductase, putative	1	10
CADAFUBP00003245	AFUB_033100	B0XVN8	Oxidoreductase 2-nitropropane dioxygenase family, putative	1	10
CADAFUBP00001651	AFUB_016830	B0XPC3	High affinity nitrate transporter NrtB	1	10
CADAFUBP00001211	AFUB_012320	B0XR70	Nitrate transporter CrnA	1	10
CADAFUBP00001210	AFUB_012310	B0XR69	Nitrite reductase NiiA	1	10
CADAFUBP00001209	AFUB_012300	B0XR68	Nitrate reductase	1	10
CADAFUBP00006309	AFUB_064840	B0Y5W7	Small nuclear ribonucleoprotein SmB, putative	1	11
CADAFUBP00006844	AFUB_070270	B0Y4T8	U1 small nuclear ribonucleoprotein C (U1 snRNP C) (U1-C) (U1C)	1	11
CADAFUBP00000875	AFUB_008940	B0XQ71	RNP domain protein	1	11
CADAFUBP00009716	AFUB_099970	B0YEQ1	Cell cycle control protein (Cwf26), putative	1	11
CADAFUBP00005715	AFUB_058430	B0Y0T1	RNA helicase-like splicing factor (HRH1), putative	1	11
CADAFUBP00006708	AFUB_068880	B0Y4F2	Splicing factor 3a subunit 2, putative	1	11
CADAFUBP00007632	AFUB_078300	B0Y8R7	RNP domain protein	1	11

CADAFUBP00003144	AFUB_032070	B0XV05	Translation initiation factor 4B	1	11
CADAFUBP00002832	AFUB_028930	B0XTC4	Uncharacterized protein	1	11
CADAFUBP00007300	AFUB_074930	B0Y7T5	FF domain protein	1	11
CADAFUBP00005980	AFUB_061210	B0Y245	Cell cycle control protein (Cwf8), putative	1	11
CADAFUBP00005187	AFUB_052980	B0Y353	Splicing factor 3b, subunit 2, 145kD	1	11
CADAFUBP00004986	AFUB_050950	B0Y2C3	Splicing factor 3a subunit 3, putative	1	11
CADAFUBP00002881	AFUB_029410	B0XTP0	Splicing factor 3B subunit 1, putative	1	11
CADAFUBP00002318	AFUB_023750	B0XW67	mRNA splicing factor RNA helicase (Cdc28), putative	1	11
CADAFUBP00002203	AFUB_022560	B0XVK4	Cell division control protein (Cdc5), putative	1	11
CADAFUBP00000994	AFUB_010130	B0XQJ0	mRNA splicing protein (Prp5), putative	1	11
CADAFUBP00000131	AFUB_001340	B0XMD7	Nuclear mRNA splicing factor, putative	1	11
CADAFUBP00003751	AFUB_038290	B0XXR1	Zinc knuckle transcription factor/splicing factor MSL5/ZFM1, putative	1	11
CADAFUBP00008837	AFUB_090890	B0YCP2	Splicing factor u2af large subunit	1	11
CADAFUBP00005977	AFUB_061180	B0Y242	U1 small nuclear ribonucleoprotein 70 kDa	1	11
CADAFUBP00005822	AFUB_059490	B0Y138	U1 small nuclear ribonucleoprotein, putative	1	11
CADAFUBP00005729	AFUB_058570	B0Y0U5	Small nuclear ribonucleoprotein U2, A	1	11
CADAFUBP00004508	AFUB_045950	B0Y0F0	U2 auxiliary factor small subunit, putative	1	11
CADAFUBP00001139	AFUB_011600	B0XQY5	Translation initiation factor eIF4E, putative	1	11
CADAFUBP00009052	AFUB_093150	B0YCT7	snRNA cap binding complex subunit (Gcr3), putative	1	11
CADAFUBP00002393	AFUB_024500	B0XWE2	Small subunit of nuclear cap-binding protein complex	1	11
CADAFUBP00005060	AFUB_051690	B0Y2R5	Translation initiation factor eIF4E3, putative	1	11
CADAFUBP00003826	AFUB_039070	B0XXZ2	Pre-RNA splicing factor Srp2, putative	1	11
CADAFUBP00006226	AFUB_064000	B0Y5N4	Methionine aminopeptidase 2-2 (MAP 2-2) (MetAP 2-2) (EC 3.4.11.18) (Peptidase M)	1	12
CADAFUBP00007137	AFUB_073300	B0Y7C2	Methionine aminopeptidase (EC 3.4.11.18)	1	12
CADAFUBP00001837	AFUB_018820	B0XTJ7	Methionine aminopeptidase 2-1 (MAP 2-1) (MetAP 2-1) (EC 3.4.11.18) (Peptidase M)	1	12
CADAFUBP00008394	AFUB_086160	B0YAX5	Methionine aminopeptidase 2-3 (MAP 2-3) (MetAP 2-3) (EC 3.4.11.18) (Peptidase M)	1	12
CADAFUBP00008389	AFUB_086110	B0YAX0	Methionine aminopeptidase (EC 3.4.11.18)	1	12
CADAFUBP00005206	AFUB_053170	B0Y372	60S ribosomal protein L23	1	12
CADAFUBP00007200	AFUB_073930	B0Y7I5	Lactoylglutathione lyase (EC 4.4.1.5) (Glyoxalase I)	1	13
CADAFUBP00006104	AFUB_062500	B0Y2V5	Glyoxalase family protein	1	13
CADAFUBP00005223	AFUB_053340	B0Y389	Ubiquitin ligase subunit HrtA, putative	1	13
CADAFUBP00009582	AFUB_098610	B0YEB7	3-hydroxybutyryl-CoA dehydrogenase, putative	1	13
CADAFUBP00004219	AFUB_043010	B0XZ35	Glutaryl-CoA dehydrogenase, putative	1	13
CADAFUBP00005641	AFUB_057660	B0Y0K7	3-hydroxyacyl-CoA dehydrogenase, putative	1	13
CADAFUBP00004221	AFUB_043030	B0XZ37	Glyoxalase family protein	1	13
CADAFUBP00008852	AFUB_091050	B0YCQ7	Electron transfer flavoprotein alpha subunit, putative	1	13
CADAFUBP00004399	AFUB_044840	B0XZQ2	Enoyl-CoA hydratase/isomerase family protein	1	13
CADAFUBP00004492	AFUB_045790	B0Y0D4	Hybrid PKS/NRPS enzyme EqiS-like, putative	1	13
CADAFUBP00004044	AFUB_041280	B0XYL0	T-complex protein 1 subunit delta	1	13
CADAFUBP00008915	AFUB_091680	B0YB61	Acyl-coenzyme A oxidase	1	13
CADAFUBP00003954	AFUB_040370	B0XYC0	Endosome-associated ubiquitin isopeptidase (AmsH), putative	1	13
CADAFUBP00008261	AFUB_084850	B0YAJ2	Acyl-CoA oxidase, putative	1	13
CADAFUBP00001164	AFUB_011850	B0XR10	WD repeat protein	1	13
CADAFUBP00001454	AFUB_014830	B0XN74	Polysaccharide deacetylase family protein	1	13
CADAFUBP00003167	AFUB_032310	B0XV93	Acyl-CoA dehydrogenase	1	13
CADAFUBP00001257	AFUB_012780	B0XRB6	SAGA complex component (Sgf29), putative	1	13
CADAFUBP00008381	AFUB_086030	B0YAW2	Hybrid NRPS/PKS enzyme, putative	1	13
CADAFUBP00007316	AFUB_075110	B0Y7V1	Oxidoreductase, short-chain dehydrogenase/reductase family	1	13
CADAFUBP00008954	AFUB_092070	B0YBA0	Acyl-CoA dehydrogenase, putative	1	13
CADAFUBP00003781	AFUB_038620	B0XXU7	3-oxoacyl-(Acyl-carrier-protein) reductase, putative	1	13
CADAFUBP00001410	AFUB_014390	B0XN30	Acyl-CoA dehydrogenase, putative	1	13
CADAFUBP00001191	AFUB_012120	B0XR37	3-ketoacyl-CoA ketothiolase (Kat1), putative	1	13

CADAFUBP00005525	AFUB_056430	B0Y441	D-arabinitol dehydrogenase ArbD, putative	1	13
CADAFUBP00009161	AFUB_094240	B0YD46	Peroxisomal D3,D2-enoyl-CoA isomerase	1	13
CADAFUBP00008316	AFUB_085400	B0YAP7	Enoyl-CoA hydratase/isomerase family protein	1	13
CADAFUBP00005634	AFUB_057590	B0Y0K0	Acyl-CoA dehydrogenase, putative	1	13
CADAFUBP00004072	AFUB_041560	B0XYN8	Enoyl-CoA hydratase/isomerase family protein	1	13
CADAFUBP00002611	AFUB_026710	B0XSC6	Enoyl-CoA hydratase/isomerase family protein	1	13
CADAFUBP00000222	AFUB_002270	B0XRF3	Enoyl-CoA hydratase/isomerase family protein	1	13
CADAFUBP00009215	AFUB_094810	B0YDA0	Nonribosomal peptide synthase, putative	1	13
CADAFUBP00006647	AFUB_068270	B0Y6V5	L-aminoadipate-semialdehyde dehydrogenase (EC 1.2.1.95) (Alpha-aminoadipate reductase)	1	13
CADAFUBP00009654	AFUB_099340	B0YEI9	Oxidoreductase, short-chain dehydrogenase/reductase family	1	13
CADAFUBP00006405	AFUB_065800	B0Y663	Short chain dehydrogenase, putative	1	13
CADAFUBP00002784	AFUB_028450	B0XT07	Polyketide synthase, putative	1	13
CADAFUBP00003259	AFUB_033250	B0XVQ2	1,3,6,8-tetrahydroxynaphthalene reductase	1	13
CADAFUBP00005529	AFUB_056470	B0Y445	3-methylcrotonyl-CoA carboxylase, beta subunit (MccB), putative	1	13
CADAFUBP00008428	AFUB_086700	B0YBI3	Polyketide synthase, putative	1	13
CADAFUBP00005602	AFUB_057200	B0Y4B8	Small monomeric GTPase (Gtr1), putative	1	13
CADAFUBP00002359	AFUB_024160	B0XWA8	NEDD8-like protein (RubA), putative	1	13
CADAFUBP00003887	AFUB_039680	B0XY53	15-hydroxyprostaglandin dehydrogenase (NAD(+))	1	13
CADAFUBP00004448	AFUB_045350	B0Y022	Oxidoreductase, short chain dehydrogenase/reductase family	1	13
CADAFUBP00003376	AFUB_034520	B0XZV6	Polyketide synthase, putative	1	13
CADAFUBP00003885	AFUB_039660	B0XY51	3-oxoacyl-(Acyl-carrier-protein) reductase	1	13
CADAFUBP00005878	AFUB_060150	B0Y1F5	Short chain dehydrogenase/reductase family	1	13
CADAFUBP00007610	AFUB_078070	B0Y8P5	Nonribosomal peptide synthase, putative	1	13
CADAFUBP00008577	AFUB_088230	B0YBY2	Polyketide synthase, putative	1	13
CADAFUBP00000212	AFUB_002170	B0XRE3	Short chain oxidoreductase/dehydrogenase, putative	1	13
CADAFUBP00008740	AFUB_089900	B0YCE5	Alcohol dehydrogenase	1	13
CADAFUBP00007631	AFUB_078290	B0Y8R6	PH domain protein	1	13
CADAFUBP00009569	AFUB_098480	B0YEA4	Short chain dehydrogenase/reductase, putative	1	13
CADAFUBP00009211	AFUB_094770	B0YD96	Short-chain dehydrogenase/reductase family protein, putative	1	13
CADAFUBP00004843	AFUB_049510	B0Y1J4	Oxidoreductase, short-chain dehydrogenase/reductase family, putative	1	13
CADAFUBP00004903	AFUB_050120	B0Y1X0	Oxidoreductase, short chain dehydrogenase/reductase family	1	13
CADAFUBP00007490	AFUB_076860	B0Y8C5	Short chain dehydrogenase/reductase family	1	13
CADAFUBP00006384	AFUB_065590	B0Y642	Acyl-CoA dehydrogenase, putative	1	13
CADAFUBP00009701	AFUB_099810	B0YEN6	Short-chain dehydrogenase/reductase family protein, putative	1	13
CADAFUBP00000360	AFUB_003670	B0XMX8	Short chain dehydrogenase/reductase, putative	1	13
CADAFUBP00007899	AFUB_081110	B0Y9I0	Short-chain dehydrogenase/reductase family protein, putative	1	13
CADAFUBP00009262	AFUB_095300	B0YDE7	Short chain dehydrogenase/reductase, putative	1	13
CADAFUBP00003047	AFUB_031090	B0XUJ3	Sorbitol/xylulose reductase Sou1-like, putative	1	13
CADAFUBP00002019	AFUB_020700	B0XUN5	Oxidoreductase, short-chain dehydrogenase/reductase family	1	13
CADAFUBP00006118	AFUB_062930	B0Y5C6	Malonyl CoA-acyl carrier protein transacylase, putative	1	13
CADAFUBP00007527	AFUB_077230	B0Y8G2	3-oxoacyl-(Acyl-carrier-protein) reductase	1	13
CADAFUBP00002308	AFUB_023650	B0XW57	Short chain dehydrogenase/reductase, putative	1	13
CADAFUBP00007461	AFUB_076570	B0Y896	3-oxoacyl-acyl carrier protein reductase	1	13
CADAFUBP00001362	AFUB_013900	B0XMR5	2-deoxy-D-gluconate 3-dehydrogenase, putative	1	13
CADAFUBP00007406	AFUB_076020	B0Y841	Oxidoreductase, short chain dehydrogenase/reductase family	1	13
CADAFUBP00008065	AFUB_082820	B0Y9Z6	Oxidoreductase, short-chain dehydrogenase/reductase family, putative	1	13
CADAFUBP00007751	NA	NA	NA	1	13
CADAFUBP00006997	AFUB_071800	B0Y591	Polyketide synthase, putative	1	13

CADAFUBP00003312	AFUB_033880	B0XZH4	Short chain dehydrogenase family protein, putative	1	13
CADAFUBP00003096	AFUB_031590	B0XUV7	Uncharacterized protein	1	13
CADAFUBP00002484	AFUB_025420	B0XRZ9	Biotin apo-protein ligase, putative	1	13
CADAFUBP00001476	AFUB_015060	B0XNG4	Urea amidolyase, putative	1	13
CADAFUBP00000933	AFUB_009510	B0XQC9	3-oxoacyl-(Acyl-carrier-protein) reductase	1	13
CADAFUBP00001379	AFUB_014070	B0XMT2	Oxidoreductase, short-chain dehydrogenase/reductase family	1	13
CADAFUBP00008404	AFUB_086260	B0YAY5	Short-chain dehydrogenase, putative	1	13
CADAFUBP00009805	AFUB_100980	B0YEZ0	Short chain dehydrogenase, putative	1	13
CADAFUBP00002110	AFUB_021610	B0XV45	Oxidoreductase, short-chain dehydrogenase/reductase family	1	13
CADAFUBP00002947	AFUB_030080	B0XU25	Oxidoreductase, short-chain dehydrogenase/reductase family	1	13
CADAFUBP00004606	AFUB_046990	B0XWQ9	Polyketide synthase, putative	1	13
CADAFUBP00007980	AFUB_081970	B0Y9R1	Oxidoreductase, short chain dehydrogenase/reductase family	1	13
CADAFUBP00006057	AFUB_062020	B0Y2J3	Short chain dehydrogenase/reductase family protein	1	13
CADAFUBP00005526	AFUB_056440	B0Y442	3-methylcrotonyl-CoA carboxylase subunit alpha (MccA), putative	1	13
CADAFUBP00005398	AFUB_055130	B0Y3R4	Methylmalonyl-CoA decarboxylase, alpha subunit, putative	1	13
CADAFUBP00003691	AFUB_037690	B0XXK1	Mitochondrial methylglutaconyl-CoA hydratase (Auh), putative	1	13
CADAFUBP00003295	AFUB_033620	B0XW03	Integral membrane protein	1	13
CADAFUBP00009224	AFUB_094910	B0YDA9	Short-chain dehydrogenase/reductase, putative	1	13
CADAFUBP00004477	AFUB_045640	B0Y051	Polyketide synthase, putative	1	13
CADAFUBP00006330	AFUB_065050	B0Y5Y8	Oxidoreductase, 2-nitropropane dioxygenase family, putative	1	13
CADAFUBP00007094	AFUB_072860	B0Y779	Short chain dehydrogenase/reductase family protein	1	13
CADAFUBP00006564	AFUB_067450	B0Y6M2	Polyubiquitin UbiD/Ubi4, putative	1	13
CADAFUBP00001322	AFUB_013440	B0XRP8	Short-chain oxidoreductase, putative	1	13
CADAFUBP00004898	AFUB_050070	B0Y1W5	Polyglutamate biosynthesis protein, putative	1	13
CADAFUBP00009862	AFUB_101550	B0YF47	Short chain dehydrogenase, putative	1	13
CADAFUBP00007055	AFUB_072470	B0Y740	Proteasome component (Ecm29), putative	1	13
CADAFUBP00005366	AFUB_054810	B0Y3N2	COP9 signalosome subunit 6 (CsnF), putative	1	13
CADAFUBP00003130	AFUB_031930	B0XUZ1	COP9 signalosome subunit 5 (CsnE), putative	1	13
CADAFUBP00009851	AFUB_101440	B0YF36	Oxidoreductase, short chain dehydrogenase/reductase family, putative	1	13
CADAFUBP00001670	AFUB_017030	B0XPK9	Short chain dehydrogenase, putative	1	13
CADAFUBP00003077	AFUB_031390	B0XUT8	Short chain dehydrogenase/oxidoreductase, putative	1	13
CADAFUBP00001364	AFUB_013920	B0XMR7	3-ketoacyl-acyl carrier protein reductase	1	13
CADAFUBP00004384	AFUB_044690	B0XZN7	PKS-like enzyme, putative	1	13
CADAFUBP00008398	AFUB_086200	B0YAX9	Polyketide synthase, putative	1	13
CADAFUBP00009081	AFUB_093440	B0YCW6	Short chain dehydrogenase/reductase family protein	1	13
CADAFUBP00009780	AFUB_100730	B0YEW5	Polyketide synthase, putative	1	13
CADAFUBP00001709	AFUB_017520	B0XST3	Short-chain dehydrogenase/reductase family protein, putative	1	13
CADAFUBP00004803	AFUB_049110	B0Y192	Peroxisomal dehydratase, putative	1	13
CADAFUBP00006024	AFUB_061680	B0Y289	Oxidoreductase, short chain dehydrogenase/reductase family	1	13
CADAFUBP00001643	AFUB_016750	B0XPB5	3-oxoacyl-(Acyl-carrier-protein) reductase, putative	1	13
CADAFUBP00004620	AFUB_047130	B0XWS3	Short-chain dehydrogenase/reductase family protein, putative	1	13
CADAFUBP00001792	AFUB_018370	B0XT84	Polyketide synthase, putative	1	13
CADAFUBP00003302	AFUB_033690	B0XW10	Short chain dehydrogenase/oxidoreductase CpoX2	1	13
CADAFUBP00006061	AFUB_062060	B0Y2J7	Oxidoreductase, short-chain dehydrogenase/reductase family, putative	1	13
CADAFUBP00001568	AFUB_016000	B0XNX4	Short chain dehydrogenase family protein	1	13
CADAFUBP00006909	AFUB_070930	B0Y503	Short chain dehydrogenase/reductase family protein	1	13
CADAFUBP00008802	AFUB_090540	B0YCK7	3-ketoacyl-acyl carrier protein reductase	1	13
CADAFUBP00003161	AFUB_032250	B0XV87	Short chain dehydrogenase/reductase, putative	1	13

CADAFUBP00003915	AFUB_039980	B0XY81	Oxidoreductase, short chain dehydrogenase/reductase family protein, putative	1	13
CADAFUBP00008495	AFUB_087420	B0YBQ0	Short chain dehydrogenase/reductase, putative	1	13
CADAFUBP00002511	AFUB_025690	B0XS26	Oxidoreductase, 2-nitropropane dioxygenase family, putative	1	13
CADAFUBP00008176	AFUB_083990	B0YAA7	Short-chain dehydrogenase/reductase 2, putative	1	13
CADAFUBP00001302	AFUB_013230	B0XRM8	Pentafunctional AROM polypeptide [Includes: 3-dehydroquinate synthase (DHQS) (EC 4.2.3.4); 3-phosphoshikimate 1-carboxyvinyltransferase (EC 2.5.1.19) (5-enolpyruvylshikimate-3-phosphate synthase) (EPSP synthase) (EPSPS); Shikimate kinase (SK) (EC 2.7.1.71); 3-dehydroquinate dehydratase (3-dehydroquinase) (EC 4.2.1.10); Shikimate dehydrogenase (EC 1.1.1.25)]	1	13
CADAFUBP00001803	AFUB_018480	B0XTG3	Short chain dehydrogenase/oxidoreductase, putative	1	13
CADAFUBP00007791	AFUB_079910	B0Y976	Short-chain dehydrogenase/reductase, putative	1	13
CADAFUBP00008995	AFUB_092480	B0YBE1	Short chain dehydrogenase/reductase, putative	1	13
CADAFUBP00003263	AFUB_033290	B0XVQ6	Polyketide synthetase PksP	1	13
CADAFUBP00000654	AFUB_006660	B0XPJ1	Short-chain dehydrogenase/reductase family protein, putative	1	13
CADAFUBP00000150	AFUB_001550	B0XLZ5	Oxidoreductase, short-chain dehydrogenase/reductase family	1	13
CADAFUBP00000089	AFUB_000900	B0XM47	Oxidoreductase, short chain dehydrogenase/reductase family	1	13
CADAFUBP00009699	AFUB_099790	B0YEN4	Oxidoreductase, short-chain dehydrogenase/reductase family	1	13
CADAFUBP00005387	AFUB_055020	B0Y3Q3	Oxidoreductase, short-chain dehydrogenase/reductase family	1	13
CADAFUBP00005552	AFUB_056700	B0Y468	Oxidoreductase, short-chain dehydrogenase/reductase family	1	13
CADAFUBP00005711	AFUB_058390	B0Y0S7	Oxidoreductase, short chain dehydrogenase/reductase family	1	13
CADAFUBP00008712	AFUB_089620	B0YCB7	3-ketoacyl-CoA thiolase (POT1), putative	1	13
CADAFUBP00006621	AFUB_068010	B0Y6S9	3-ketoacyl-coA thiolase peroxisomal A	1	13
CADAFUBP00002650	AFUB_027110	B0XSG5	Peroxisomal 3-ketoacyl-coA thiolase (Kat1), putative	1	13
CADAFUBP00008757	AFUB_090080	B0YCG2	Short chain dehydrogenase/reductase family	1	13
CADAFUBP00005124	AFUB_052350	B0Y2Z0	Oxidoreductase, short chain dehydrogenase/reductase family	1	13
CADAFUBP00008282	AFUB_085060	B0YAL3	Oxidoreductase, short chain dehydrogenase/reductase family	1	13
CADAFUBP00001675	AFUB_017080	B0XPL4	Short chain dehydrogenase, putative	1	13
CADAFUBP00006121	AFUB_062960	B0Y5C9	Oxidoreductase, short-chain dehydrogenase/reductase family	1	13
CADAFUBP00007023	AFUB_072060	B0Y5B7	Short chain dehydrogenase, putative	1	13
CADAFUBP00009153	AFUB_094160	B0YD38	Short chain dehydrogenase/reductase family protein	1	13
CADAFUBP00009776	AFUB_100680	B0YEW1	Short-chain dehydrogenase, putative	1	13
CADAFUBP00007882	AFUB_080930	B0Y9G3	Oxidoreductase, short chain dehydrogenase/reductase family	1	13
CADAFUBP00008134	AFUB_083570	B0YA65	Acetyl-CoA acetyltransferase, putative	1	13
CADAFUBP00008057	AFUB_082740	B0Y9Y8	Acetoacetyl-CoA synthase	1	13
CADAFUBP00007846	AFUB_080560	B0Y9C7	Hydroxymethylglutaryl-CoA synthase, putative	1	13
CADAFUBP00004309	AFUB_043930	B0XZC5	Uncharacterized protein	1	13
CADAFUBP00003769	AFUB_038500	B0XXT5	Hydroxymethylglutaryl-CoA synthase, putative	1	13
CADAFUBP00001112	AFUB_011330	B0XQV8	Acetoacetyl-CoA synthase	1	13
CADAFUBP00000055	AFUB_000550	B0XMC1	Acetyl-CoA-acetyltransferase, putative	1	13
CADAFUBP00001024	AFUB_010430	B0XQM0	Short chain oxidoreductase/dehydrogenase, putative	1	13
CADAFUBP00008199	AFUB_084240	B0YAD0	PKS-like enzyme, putative	1	13
CADAFUBP00001789	AFUB_018340	B0XT81	HLH transcription factor, putative	1	13
CADAFUBP00005647	AFUB_057720	B0Y0L3	Hybrid NRPS/PKS enzyme, putative	1	13
CADAFUBP00000081	AFUB_000820	B0XM39	Polyketide synthase, putative	1	13
CADAFUBP00004541	AFUB_046300	B0Y0I3	Short-chain dehydrogenase/reductase, putative	1	13
CADAFUBP00009632	AFUB_099120	B0YEG7	Peroxisomal multifunctional beta-oxidation protein (MFP), putative	1	13

CADAFUBP00007032	AFUB_072240	B0Y717	Histone-lysine N-methyltransferase (EC 2.1.1.43)	1	13
CADAFUBP00005084	AFUB_051950	B0Y2T9	PHD transcription factor (Rum1), putative	1	13
CADAFUBP00009283	AFUB_095530	B0YDG8	Short-chain dehydrogenase/reductase family protein, putative	1	13
CADAFUBP00005565	AFUB_056830	B0Y481	NAD-dependent 15-hydroxyprostaglandin dehydrogenase	1	13
CADAFUBP00007474	AFUB_076700	B0Y8A9	ATP citrate lyase subunit (Acl), putatibe	1	13
CADAFUBP00007473	AFUB_076690	B0Y8A8	ATP citrate lyase, subunit 1, putative	1	13
CADAFUBP00005597	AFUB_057150	B0Y4B3	2-nitropropane dioxygenase family oxidoreductase, putative	1	13
CADAFUBP00002402	AFUB_024590	B0XRR7	Acetyl-CoA carboxylase	1	13
CADAFUBP00002226	AFUB_022790	B0XVU4	3-oxoacyl-[acyl-carrier-protein] synthase	1	13
CADAFUBP00005576	AFUB_056940	B0Y492	Carbonyl reductase, putative	1	13
CADAFUBP00004404	AFUB_044900	B0XZQ7	Nonribosomal peptide synthase SidE	1	13
CADAFUBP00006864	AFUB_070470	B0Y4V8	Short chain dehydrogenase/reductase family	1	13
CADAFUBP00004295	AFUB_043770	B0XZB1	Fatty acid synthase alpha subunit FasA, putative	1	13
CADAFUBP00004294	AFUB_043760	B0XZB0	Fatty acid synthase beta subunit, putative	1	13
CADAFUBP00001609	AFUB_016410	B0XP81	UDP-glucose dehydrogenase	1	14
CADAFUBP00004232	AFUB_043140	B0XZ48	Transcription elongation factor SPT6, putative	1	14
CADAFUBP00008594	AFUB_088400	B0YBZ9	UTP-glucose-1-phosphate uridylyltransferase Ugp1, putative	1	14
CADAFUBP00008343	AFUB_085660	B0YAS4	UDP-glucose dehydrogenase Ugd1, putative	1	14
CADAFUBP00006941	AFUB_071250	B0Y535	UDP-glucose 4-epimerase	1	14
CADAFUBP00005710	AFUB_058380	B0Y0S6	UDP-glucose 4-epimerase	1	14
CADAFUBP00004037	AFUB_041210	B0XYK3	UDP-glucose 4-epimerase, putative	1	14
CADAFUBP00002670	AFUB_027310	B0XSI5	Galactose-1-phosphate uridylyltransferase (EC 2.7.7.12)	1	14
CADAFUBP00009105	AFUB_093680	B0YCZ0	NADH-ubiquinone oxidoreductase B14 subunit, putative	1	15
CADAFUBP00007284	AFUB_074770	B0Y7R9	NADH-ubiquinone oxidoreductase 304 kDa subunit	1	15
CADAFUBP00006212	AFUB_063860	B0Y5M0	Cytochrome b-c1 complex subunit 7	1	15
CADAFUBP00005019	AFUB_051280	B0Y2F6	Cytochrome c oxidase subunit Va, putative	1	15
CADAFUBP00004205	AFUB_042870	B0XZ21	Cytochrome c oxidase subunit VIa, putative	1	15
CADAFUBP00001960	AFUB_020070	B0XUA7	Cytochrome c subunit Vb, putative	1	15
CADAFUBP00001531	AFUB_015620	B0XNT7	Mitochondrial F1F0-ATP synthase g subunit, putative	1	15
CADAFUBP00001276	AFUB_012970	B0XRK2	Ubiquinol-cytochrome C reductase complex subunit UcrQ, putative	1	15
CADAFUBP00000344	AFUB_003510	B0XMW2	ATP synthase delta chain, mitochondrial, putative	1	15
CADAFUBP00000688	AFUB_007000	B0XPN4	Acyl carrier protein	1	15
CADAFUBP00000172	AFUB_001770	B0XM00	Nuclear distribution protein nudF (Lissencephaly-1 homolog) (LIS-1)	2	1
CADAFUBP00006407	AFUB_065820	B0Y665	Lysophospholipase 1 (EC 3.1.1.5) (Phospholipase B 1)	2	1
CADAFUBP00004876	NA	NA	NA	2	1
CADAFUBP00009732	AFUB_100130	B0YER7	Lysophospholipase NTE1 (EC 3.1.1.5) (Intracellular phospholipase B)	2	1
CADAFUBP00009079	AFUB_093420	B0YCW4	MBOAT family protein, putative	2	1
CADAFUBP00007627	AFUB_078250	B0Y8R2	Glycerophosphoryl diester phosphodiesterase family protein	2	1
CADAFUBP00003378	AFUB_034540	B0XZV8	Lysophospholipase 3 (EC 3.1.1.5) (Phospholipase B 3)	2	1
CADAFUBP00001763	AFUB_018080	B0XT55	Glycerophosphoryl diester phosphodiesterase family protein	2	1
CADAFUBP00009777	AFUB_100690	B0YEW2	Fatty acid oxygenase, putative	2	1
CADAFUBP00006604	AFUB_067850	B0Y6R2	Psi-producing oxygenase A (Fatty acid oxygenase ppoA) [Includes: Linoleate 8R-lipoxygenase (EC 1.13.11.60); 9,12-octadecadienoate 8-hydroperoxide 8R-isomerase (EC 5.4.4.5)]	2	1
CADAFUBP00006379	AFUB_065540	B0Y637	Patatin-like phospholipase domain-containing protein (EC 3.1.1.-)	2	1
CADAFUBP00003628	AFUB_037060	B0XX73	Fatty acid oxygenase PpoC, putative	2	1
CADAFUBP00002707	AFUB_027690	B0XSM2	Lysophospholipase (EC 3.1.1.5)	2	1
CADAFUBP00000655	AFUB_006670	B0XPJ2	Small nucleolar ribonucleoprotein complex subunit	3	1

			(SOF1), putative		
CADAFUBP00004164	AFUB_042470	B0XYY0	WD repeat protein	3	1
CADAFUBP00007043	AFUB_072350	B0Y728	rRNA processing protein (Rrp20), putative	3	1
CADAFUBP00001840	AFUB_018850	B0XTK0	Small nucleolar ribonucleoprotein complex subunit Utp15, putative	3	1
CADAFUBP00001234	AFUB_012550	B0XR93	U3 small nucleolar ribonucleoprotein protein IMP4, putative	3	1
CADAFUBP00004222	AFUB_043040	B0XZ38	RNA processing protein Emg1, putative	3	1
CADAFUBP00001934	AFUB_019810	B0XU13	60S ribosomal protein L24b	3	1
CADAFUBP00009106	AFUB_093690	B0YCZ1	DNA-directed RNA polymerase I and III 14 kDa polypeptide	3	1
CADAFUBP00008055	AFUB_082720	B0Y9Y6	Ribosome biogenesis protein, putative	3	1
CADAFUBP00007233	AFUB_074260	B0Y7L8	DNA-directed RNA polymerase subunit beta (EC 2.7.7.6)	3	1
CADAFUBP00006298	AFUB_064720	B0Y5V6	Ribosome biogenesis protein ytm1	3	1
CADAFUBP00002875	AFUB_029350	B0XTN4	DNA-directed RNA polymerase I and III subunit Rpc40, putative	3	1
CADAFUBP00002245	AFUB_022990	B0XVW3	SnRNP and snoRNP protein (Snu13), putative	3	1
CADAFUBP00001285	AFUB_013060	B0XRL1	Small nuclear ribonucleoprotein complex protein Nhp2, putative	3	1
CADAFUBP00005189	AFUB_053000	B0Y355	Nucleolar RNAse III, putative	3	1
CADAFUBP00002371	AFUB_024280	B0XWC0	U3 small nucleolar ribonucleoprotein subunit (Imp3), putative	3	1
CADAFUBP00002197	AFUB_022500	B0XVJ8	DNA-directed RNA polymerase subunit	3	1
CADAFUBP00007038	AFUB_072300	B0Y723	Methyltransferase, putative	3	1
CADAFUBP00005236	AFUB_053470	B0Y3A2	RNA processing factor 1	3	1
CADAFUBP00004303	AFUB_043850	B0XZB9	rRNA processing protein Bystin, putative	3	1
CADAFUBP00008747	AFUB_089980	B0YCF2	Ribosome biogenesis protein (Rrs1), putative	3	1
CADAFUBP00006172	AFUB_063470	B0Y5I0	Ribosomal biogenesis protein Gar2	3	1
CADAFUBP00005976	AFUB_061170	B0Y241	60S acidic ribosomal protein P0, putative	3	1
CADAFUBP00003207	AFUB_032720	B0XVD3	60S ribosome subunit biogenesis protein NIP7	3	1
CADAFUBP00002789	AFUB_028500	B0XT12	Pre-rRNA processing protein Tsr1, putative	3	1
CADAFUBP00002131	AFUB_021840	B0XV66	Pre-rRNA processing protein, putative	3	1
CADAFUBP00001039	AFUB_010590	B0XQN5	Sas10/Utp3 family protein	3	1
CADAFUBP00000254	AFUB_002590	B0XRI5	60S ribosome biogenesis protein Brx1, putative	3	1
CADAFUBP00005585	AFUB_057030	B0Y4A1	ABC transporter, putative	4	1
CADAFUBP00009863	AFUB_101559	B0YF48	ABC multidrug transporter, putative	4	1
CADAFUBP00007208	AFUB_074010	B0Y7J3	ABC transporter, putative	4	1
CADAFUBP00007131	AFUB_073240	B0Y7B6	ABC transporter (Adp1), putative	4	1
CADAFUBP00005583	AFUB_057010	B0Y499	ABC transporter, putative	4	1
CADAFUBP00004970	AFUB_050790	B0Y2A7	ABC multidrug transporter, putative	4	1
CADAFUBP00004817	AFUB_049250	B0Y1A6	ABC multidrug transporter, putative	4	1
CADAFUBP00004607	AFUB_047000	B0XWR0	ABC multidrug transporter, putative	4	1
CADAFUBP00004094	AFUB_041770	B0XYR0	ABC multidrug transporter, putative	4	1
CADAFUBP00002904	AFUB_029650	B0XTR3	ABC transporter, putative	4	1
CADAFUBP00001360	AFUB_013880	B0XMR3	ABC transporter, putative	4	1
CADAFUBP00006218	AFUB_063920	B0Y5M6	Coenzyme A synthetase, putative	5	1
CADAFUBP00000576	AFUB_005860	B0XP43	Mandelate racemase/muconate lactonizing enzyme family protein	5	1
CADAFUBP00001238	AFUB_012590	B0XR97	4-coumarate-CoA ligase, putative	5	1
CADAFUBP00002507	AFUB_025650	B0XS22	Mandelate racemase/muconate lactonizing enzyme family protein	5	1
CADAFUBP00008564	AFUB_088100	B0YBW9	AMP-binding enzyme, putative	5	1
CADAFUBP00002538	AFUB_025970	B0XS53	Phenylacetyl-CoA ligase, putative	5	1
CADAFUBP00005881	AFUB_060180	B0Y1F8	Adenylate-forming enzyme AfeA	5	1
CADAFUBP00005483	AFUB_056010	B0Y3Z9	AMP-binding enzyme, putative	5	1
CADAFUBP00006053	AFUB_061980	B0Y2I9	AMP dependent CoA ligase	5	1
CADAFUBP00000082	AFUB_000830	B0XM40	Adenylate-forming enzyme, putative	5	1
CADAFUBP00008739	AFUB_089890	B0YCE4	Mandelate racemase/muconate lactonizing enzyme family protein	5	1

CADAFUBP00001626	AFUB_016580	B0XP98	Long-chain-fatty-acid-CoA ligase, putative	5	1
CADAFUBP00000625	AFUB_006370	B0XPG2	AMP-binding domain protein, putative	5	1

Supplementary Table 3 | Sub-module 1 cluster 1, significantly enriched GO terms (biological processes)

GO-ID	Term	Count	Size	p-value
GO:0006694	steroid biosynthetic process	14	21	2.97353E-12
GO:0055114	oxidation-reduction process	39	211	1.52684E-10
GO:0016128	phytosteroid metabolic process	11	17	1.41806E-09
GO:0044107	cellular alcohol metabolic process	11	17	1.41806E-09
GO:0006696	ergosterol biosynthetic process	11	17	1.41806E-09
GO:0016125	sterol metabolic process	11	18	3.43632E-09
GO:1902652	secondary alcohol metabolic process	12	23	7.25617E-09
GO:0046165	alcohol biosynthetic process	13	32	7.24412E-08
GO:0044255	cellular lipid metabolic process	17	71	3.83852E-06
GO:0008202	steroid metabolic process	4	4	1.15868E-05
GO:0006633	fatty acid biosynthetic process	6	15	0.00039
GO:0044699	single-organism process	50	513	0.00127
GO:0044711	single-organism biosynthetic process	26	206	0.00156
GO:0016104	triterpenoid biosynthetic process	2	2	0.00541

Supplementary Table 4 | Sub-module 1 cluster 2, significantly enriched GO terms (biological processes)

GO-ID	Term	Count	Size	p-value
GO:0006979	response to oxidative stress	6	28	3.25040E-06
GO:1901700	response to oxygen-containing compound	5	16	3.40598E-06
GO:0006801	superoxide metabolic process	3	3	3.70766E-06
GO:0007571	age-dependent general metabolic decline	3	3	4.54815E-06
GO:0019363	pyridine nucleotide biosynthetic process	3	4	1.79959E-05
GO:0009435	NAD biosynthetic process	3	4	1.79959E-05
GO:0006468	protein phosphorylation	4	17	0.00014
GO:0006586	indolalkylamine metabolic process	3	7	0.00015
GO:0001320	age-dependent response to reactive oxygen species involved in chronological cell aging	2	2	0.00029
GO:0001315	age-dependent response to reactive oxygen species	2	2	0.00029
GO:0070887	cellular response to chemical stimulus	6	63	0.00041
GO:0034354	'de novo' NAD biosynthetic process from tryptophan	2	3	0.00087
GO:0006796	phosphate-containing compound metabolic process	7	104	0.00103
GO:0036211	protein modification process	5	50	0.00116
GO:0044106	cellular amine metabolic process	3	13	0.00117
GO:0044699	single-organism process	15	513	0.00139
GO:0010035	response to inorganic substance	3	14	0.00147
GO:0042436	indole-containing compound catabolic process	2	4	0.00172
GO:0038202	TORC1 signaling	2	4	0.00172
GO:0006569	tryptophan catabolic process	2	4	0.00172
GO:0050896	response to stimulus	5	70	0.00178
GO:0009072	aromatic amino acid family metabolic process	3	17	0.00266
GO:0042402	cellular biogenic amine catabolic process	2	5	0.00284
GO:0010648	negative regulation of cell communication	2	5	0.00284
GO:0023057	negative regulation of signaling	2	5	0.00284
GO:1901293	nucleoside phosphate biosynthetic process	3	18	0.00315
GO:0001302	replicative cell aging	2	6	0.00422
GO:0043620	regulation of DNA-templated transcription in response to stress	2	6	0.00422
GO:0070301	cellular response to hydrogen peroxide	2	6	0.00422
GO:0019438	aromatic compound biosynthetic process	6	97	0.00428
GO:0018130	heterocycle biosynthetic process	6	106	0.00467
GO:0051188	cofactor biosynthetic process	3	23	0.00648
GO:0046496	nicotinamide nucleotide metabolic process	3	26	0.00921
GO:0072524	pyridine-containing compound metabolic process	3	26	0.00921

Supplementary Table 5 | Sub-module 1 cluster 3, significantly enriched GO terms (biological processes)

GO-ID	Term	Count	Size	p-value
GO:0006897	endocytosis	5	6	2.40413E-08
GO:0030029	actin filament-based process	5	6	2.40413E-08
GO:0007010	cytoskeleton organization	5	9	4.82736E-07
GO:0007015	actin filament organization	4	5	1.06152E-06
GO:0016311	dephosphorylation	4	7	7.20793E-06
GO:0000147	actin cortical patch assembly	3	3	1.08019E-05
GO:0046856	phosphatidylinositol dephosphorylation	3	3	1.08019E-05
GO:0030258	lipid modification	4	8	1.41981E-05
GO:1902589	single-organism organelle organization	5	16	1.50689E-05
GO:0051234	establishment of localization	5	24	7.00960E-05
GO:0006650	glycerophospholipid metabolic process	4	12	9.44658E-05
GO:0030242	pexophagy	2	3	0.00152
GO:0045324	late endosome to vacuole transport	2	3	0.00152
GO:0016043	cellular component organization	7	92	0.00317
GO:0072665	protein localization to vacuole	2	5	0.00492
GO:0030010	establishment of cell polarity	2	5	0.00492
GO:0016236	macroautophagy	2	6	0.00728
GO:0070727	cellular macromolecule localization	4	37	0.0086

Supplementary Table 6 | Sub-module 1 cluster 4, significantly enriched GO terms (biological processes)

GO-ID	Term	Count	Size	p-value
GO:0008299	isoprenoid biosynthetic process	4	5	1.28999E-08
GO:0044255	cellular lipid metabolic process	6	71	3.10319E-05

Supplementary Table 7 | Sub-module 1 cluster 5, significantly enriched GO terms (biological processes)

GO-ID	Term	Count	Size	p-value
GO:0036211	protein modification process	8	50	2.07199E-08
GO:0044700	single organism signaling	5	17	2.66968E-08
GO:0006468	protein phosphorylation	5	13	3.21622E-08
GO:0044764	multi-organism cellular process	6	20	4.40182E-08
GO:0048315	conidium formation	5	12	7.14998E-08
GO:0051347	positive regulation of transferase activity	4	5	8.85073E-08
GO:0042327	positive regulation of phosphorylation	4	5	8.85073E-08
GO:0065007	biological regulation	7	96	1.35817E-07
GO:0000909	sporocarp development involved in sexual reproduction	5	13	1.93373E-07
GO:0019954	asexual reproduction	6	26	2.52492E-07
GO:0031401	positive regulation of protein modification process	4	6	2.63635E-07
GO:0030582	reproductive fruiting body development	5	14	2.98841E-07
GO:0010562	positive regulation of phosphorus metabolic process	4	7	6.10773E-07
GO:0031135	negative regulation of conjugation	4	7	6.10773E-07
GO:0048608	reproductive structure development	5	16	6.43530E-07
GO:0044707	single-multicellular organism process	5	16	6.43530E-07
GO:0048731	system development	5	16	6.43530E-07
GO:0045930	negative regulation of mitotic cell cycle	4	8	1.21285E-06
GO:0009628	response to abiotic stimulus	6	34	1.40746E-06
GO:0051716	cellular response to stimulus	7	83	1.71546E-06
GO:0007050	cell cycle arrest	3	3	1.78677E-06
GO:0032147	activation of protein kinase activity	3	3	1.78677E-06
GO:0071474	cellular hyperosmotic response	3	3	1.78677E-06
GO:0043902	positive regulation of multi-organism process	4	9	2.16759E-06
GO:2000242	negative regulation of reproductive process	4	10	3.58691E-06
GO:0051247	positive regulation of protein metabolic process	4	10	3.58691E-06
GO:0048522	positive regulation of cellular process	5	34	6.45735E-06
GO:0010647	positive regulation of cell communication	3	4	7.09360E-06
GO:0023056	positive regulation of signaling	3	4	7.09360E-06
GO:1902533	positive regulation of intracellular signal transduction	3	4	7.09360E-06
GO:0044267	cellular protein metabolic process	7	90	7.61259E-06
GO:0071840	cellular component organization or biogenesis	8	112	1.40847E-05
GO:0051128	regulation of cellular component organization	4	17	1.70837E-05
GO:0032505	reproduction of a single-celled organism	4	15	2.24945E-05
GO:0034293	sexual sporulation	4	15	2.24945E-05
GO:0031139	positive regulation of conjugation with cellular fusion	3	6	2.63568E-05
GO:0044093	positive regulation of molecular function	3	6	2.64440E-05
GO:0007154	cell communication	3	14	3.32168E-05
GO:0006950	response to stress	6	71	3.41614E-05
GO:0019953	sexual reproduction	3	11	4.93026E-05
GO:0070787	conidiophore development	3	7	6.06849E-05
GO:0043455	regulation of secondary metabolic process	3	7	6.06849E-05
GO:0042325	regulation of phosphorylation	2	2	6.92127E-05
GO:0051174	regulation of phosphorus metabolic process	2	2	6.95215E-05
GO:0030435	sporulation resulting in formation of a cellular spore	4	20	7.70249E-05
GO:0048584	positive regulation of response to stimulus	4	20	7.70249E-05
GO:0000165	MAPK cascade	2	2	8.85934E-05
GO:1901990	regulation of mitotic cell cycle phase transition	3	8	9.63681E-05
GO:0009889	regulation of biosynthetic process	4	26	0.00011
GO:0010605	negative regulation of macromolecule metabolic process	4	22	0.00011
GO:1903533	regulation of protein targeting	2	2	0.00016
GO:1902074	response to salt	2	2	0.00016

GO:0045936	negative regulation of phosphate metabolic process	2	2	0.00016
GO:0010969	regulation of pheromone-dependent signal transduction involved in conjugation with cellular fusion	2	2	0.00016
GO:1903936	cellular response to sodium arsenite	2	2	0.00016
GO:0060240	negative regulation of signal transduction involved in conjugation with cellular fusion	2	2	0.00016
GO:0043406	positive regulation of MAP kinase activity	2	2	0.00016
GO:0000768	syncytium formation by plasma membrane fusion	2	2	0.00016
GO:0046685	response to arsenic-containing substance	2	2	0.00016
GO:0070302	regulation of stress-activated protein kinase signaling cascade	2	2	0.00016
GO:0071851	mitotic G1 cell cycle arrest in response to nitrogen starvation	2	2	0.00016
GO:0030437	ascospore formation	3	11	0.00021
GO:0051726	regulation of cell cycle	2	3	0.00021
GO:1903506	regulation of nucleic acid-templated transcription	4	26	0.00023
GO:0051252	regulation of RNA metabolic process	4	26	0.00023
GO:0006796	phosphate-containing compound metabolic process	6	102	0.00026
GO:0071310	cellular response to organic substance	3	12	0.00037
GO:0010515	negative regulation of induction of conjugation with cellular fusion	2	3	0.0004
GO:0006366	transcription from RNA polymerase II promoter	3	14	0.00046
GO:0051171	regulation of nitrogen compound metabolic process	3	17	0.00047
GO:1901989	positive regulation of cell cycle phase transition	2	3	0.00048
GO:0060341	regulation of cellular localization	2	3	0.00048
GO:0019932	second-messenger-mediated signaling	2	3	0.00048
GO:0090087	regulation of peptide transport	2	3	0.00048
GO:1901362	organic cyclic compound biosynthetic process	7	132	0.00051
GO:2000112	regulation of cellular macromolecule biosynthetic process	4	35	0.00052
GO:0080090	regulation of primary metabolic process	3	28	0.00054
GO:0045229	external encapsulating structure organization	3	14	0.0006
GO:0031399	regulation of protein modification process	2	5	0.00068
GO:0043900	regulation of multi-organism process	2	5	0.00069
GO:1903046	meiotic cell cycle process	3	16	0.00069
GO:0019438	aromatic compound biosynthetic process	6	97	0.00071
GO:0007163	establishment or maintenance of cell polarity	3	15	0.00074
GO:0045931	positive regulation of mitotic cell cycle	2	4	0.0008
GO:0009651	response to salt stress	2	4	0.0008
GO:0009893	positive regulation of metabolic process	2	5	0.00091
GO:1900436	positive regulation of filamentous growth of a population of unicellular organisms in response to starvation	2	4	0.00095
GO:0019236	response to pheromone	2	4	0.00095
GO:0032101	regulation of response to external stimulus	2	4	0.00095
GO:0032106	positive regulation of response to extracellular stimulus	2	4	0.00095
GO:0032107	regulation of response to nutrient levels	2	4	0.00095
GO:1902749	regulation of cell cycle G2/M phase transition	2	4	0.00095
GO:0090066	regulation of anatomical structure size	2	4	0.00095
GO:0090033	positive regulation of filamentous growth	2	4	0.00095
GO:0071470	cellular response to osmotic stress	2	6	0.00102
GO:0010468	regulation of gene expression	4	38	0.00103
GO:0042221	response to chemical	4	57	0.00151
GO:0010648	negative regulation of cell communication	2	5	0.00158
GO:0023057	negative regulation of signaling	2	5	0.00158
GO:0031329	regulation of cellular catabolic process	2	5	0.00158
GO:0030448	hyphal growth	3	21	0.00207
GO:1903047	mitotic cell cycle process	3	21	0.00207
GO:0090304	nucleic acid metabolic process	5	79	0.00217
GO:0043620	regulation of DNA-templated transcription in response to stress	2	6	0.00235

GO:0051170	nuclear import	2	6	0.00235
GO:0043937	regulation of sporulation	2	6	0.00235
GO:0044271	cellular nitrogen compound biosynthetic process	6	121	0.00236
GO:0035556	intracellular signal transduction	2	12	0.0024
GO:0033044	regulation of chromosome organization	2	7	0.00274
GO:1900428	regulation of filamentous growth of a population of unicellular organisms	2	7	0.00327
GO:0032269	negative regulation of cellular protein metabolic process	2	7	0.00327
GO:0048585	negative regulation of response to stimulus	2	7	0.00327
GO:0042542	response to hydrogen peroxide	2	8	0.00432
GO:0022414	reproductive process	2	16	0.00444
GO:0048468	cell development	2	13	0.00522
GO:0018130	heterocycle biosynthetic process	5	106	0.00523
GO:0048285	organelle fission	2	10	0.00684
GO:0051246	regulation of protein metabolic process	2	15	0.00693
GO:0031505	fungal-type cell wall organization	2	11	0.00699
GO:0010564	regulation of cell cycle process	2	11	0.00702
GO:0035690	cellular response to drug	3	32	0.00712
GO:0031668	cellular response to extracellular stimulus	3	33	0.00777
GO:1902582	single-organism intracellular transport	2	11	0.0083
GO:0045893	positive regulation of transcription, DNA-templated	2	12	0.00989
GO:0040008	regulation of growth	2	12	0.00989
GO:1902680	positive regulation of RNA biosynthetic process	2	12	0.00989
GO:0034248	regulation of cellular amide metabolic process	2	12	0.00989

Supplementary Table 8 | Sub-module 1 cluster 6, significantly enriched GO terms (biological processes)

GO-ID	Term	Count	Size	p-value
GO:0006082	organic acid metabolic process	111	191	6.43205E-29
GO:0006520	cellular amino acid metabolic process	51	66	4.18215E-22
GO:1901607	alpha-amino acid biosynthetic process	26	30	3.36716E-13
GO:0046394	carboxylic acid biosynthetic process	52	93	1.91656E-11
GO:0006807	nitrogen compound metabolic process	112	293	7.17667E-10
GO:0055114	oxidation-reduction process	88	211	9.17399E-10
GO:0046395	carboxylic acid catabolic process	29	44	5.79554E-09
GO:0009066	aspartate family amino acid metabolic process	18	21	6.17613E-09
GO:0044710	single-organism metabolic process	75	236	7.19873E-08
GO:0044283	small molecule biosynthetic process	56	124	7.66122E-08
GO:1901565	organonitrogen compound catabolic process	21	30	1.53891E-07
GO:0006099	tricarboxylic acid cycle	12	14	2.87753E-06
GO:0044282	small molecule catabolic process	30	58	6.39517E-06
GO:1901566	organonitrogen compound biosynthetic process	51	125	9.3497E-06
GO:1901606	alpha-amino acid catabolic process	14	20	2.09419E-05
GO:0044272	sulfur compound biosynthetic process	11	14	3.14417E-05
GO:0072350	tricarboxylic acid metabolic process	10	12	3.22308E-05
GO:0045333	cellular respiration	14	21	6.02294E-05
GO:0009064	glutamine family amino acid metabolic process	6	6	0.00019
GO:0009086	methionine biosynthetic process	7	8	0.00038
GO:0000096	sulfur amino acid metabolic process	5	5	0.00089
GO:0006536	glutamate metabolic process	5	5	0.00092
GO:0044273	sulfur compound catabolic process	5	5	0.00098
GO:0009098	leucine biosynthetic process	5	5	0.00098
GO:0009065	glutamine family amino acid catabolic process	5	5	0.00098
GO:0006767	water-soluble vitamin metabolic process	5	5	0.00098
GO:0006526	arginine biosynthetic process	5	5	0.00098
GO:0043648	dicarboxylic acid metabolic process	6	7	0.00104
GO:0006637	acyl-CoA metabolic process	8	11	0.00117
GO:0006575	cellular modified amino acid metabolic process	6	7	0.00123
GO:0006067	ethanol metabolic process	6	7	0.00134
GO:0006534	cysteine metabolic process	6	7	0.00134
GO:0009082	branched-chain amino acid biosynthetic process	4	4	0.00354
GO:0042398	cellular modified amino acid biosynthetic process	4	4	0.00379
GO:1902221	erythrose 4-phosphate/phosphoenolpyruvate family amino acid metabolic process	4	4	0.00395
GO:0006108	malate metabolic process	4	4	0.00395
GO:0009110	vitamin biosynthetic process	4	4	0.00395
GO:0006570	tyrosine metabolic process	4	4	0.00395
GO:0006537	glutamate biosynthetic process	4	4	0.00395
GO:0006085	acetyl-CoA biosynthetic process	5	6	0.00467
GO:0009092	homoserine metabolic process	5	6	0.00467
GO:0035384	thioester biosynthetic process	5	6	0.00467
GO:0006749	glutathione metabolic process	5	6	0.00467
GO:0006567	threonine catabolic process	5	6	0.00467
GO:0019752	carboxylic acid metabolic process	8	19	0.00829
GO:0009072	aromatic amino acid family metabolic process	6	9	0.00915
GO:0006090	pyruvate metabolic process	10	20	0.01369

Supplementary Table 9 | Sub-module 1 cluster 7, significantly enriched GO terms (biological processes)

GO-ID	Term	Count	Size	p-value
GO:0005975	carbohydrate metabolic process	32	41	7.89252E-34
GO:0044723	single-organism carbohydrate metabolic process	28	43	8.66977E-19
GO:0016052	carbohydrate catabolic process	17	18	2.58370E-16
GO:0005984	disaccharide metabolic process	13	16	4.32191E-10
GO:0006165	nucleoside diphosphate phosphorylation	10	10	7.04419E-10
GO:0072524	pyridine-containing compound metabolic process	15	26	3.13769E-08
GO:0046031	ADP metabolic process	10	12	3.71878E-08
GO:0009185	ribonucleoside diphosphate metabolic process	10	12	3.71878E-08
GO:0009135	purine nucleoside diphosphate metabolic process	10	12	3.71878E-08
GO:0006096	glycolytic process	8	8	4.47368E-08
GO:0006733	oxidoreduction coenzyme metabolic process	15	27	6.31905E-08
GO:0005992	trehalose biosynthetic process	8	9	4.09715E-07
GO:0009312	oligosaccharide biosynthetic process	8	9	4.09715E-07
GO:0005976	polysaccharide metabolic process	11	18	8.53380E-07
GO:0044712	single-organism catabolic process	27	89	2.07454E-06
GO:0046835	carbohydrate phosphorylation	6	6	3.55357E-06
GO:0044275	cellular carbohydrate catabolic process	9	14	4.59487E-06
GO:0034637	cellular carbohydrate biosynthetic process	7	9	8.26346E-06
GO:0033500	carbohydrate homeostasis	7	9	1.23321E-05
GO:0001678	cellular glucose homeostasis	7	9	1.23322E-05
GO:0005996	monosaccharide metabolic process	9	16	1.65953E-05
GO:0046034	ATP metabolic process	10	21	7.27782E-05
GO:0009199	ribonucleoside triphosphate metabolic process	10	21	7.27782E-05
GO:0009144	purine nucleoside triphosphate metabolic process	10	21	7.27782E-05
GO:0006098	pentose-phosphate shunt	6	9	0.00022
GO:0046164	alcohol catabolic process	6	9	0.00022
GO:0055086	nucleobase-containing small molecule metabolic process	17	56	0.00022
GO:0005987	sucrose catabolic process	4	4	0.00024
GO:0006013	mannose metabolic process	4	4	0.00024
GO:0000272	polysaccharide catabolic process	4	4	0.00024
GO:0006091	generation of precursor metabolites and energy	14	42	0.00029
GO:0006006	glucose metabolic process	6	10	0.00036
GO:0065008	regulation of biological quality	14	43	0.00038
GO:0051186	cofactor metabolic process	15	49	0.00049
GO:0046365	monosaccharide catabolic process	5	7	0.0005
GO:0009313	oligosaccharide catabolic process	5	7	0.0005
GO:0009225	nucleotide-sugar metabolic process	5	7	0.0005
GO:0006793	phosphorus metabolic process	26	113	0.0007
GO:0044419	interspecies interaction between organisms	14	46	0.00083
GO:0009126	purine nucleoside monophosphate metabolic process	10	27	0.00089
GO:0019405	alditol catabolic process	4	5	0.00102
GO:0071554	cell wall organization or biogenesis	8	19	0.00114
GO:0046364	monosaccharide biosynthetic process	5	8	0.0012
GO:0009161	ribonucleoside monophosphate metabolic process	10	29	0.00169
GO:0019725	cellular homeostasis	8	20	0.0017
GO:0051817	modification of morphology or physiology of other organism involved in symbiotic interaction	6	12	0.00172
GO:0044416	induction by symbiont of host defense response	6	12	0.00172
GO:0052031	modulation by symbiont of host defense response	6	12	0.00172
GO:0052510	positive regulation by organism of defense response of other organism involved in symbiotic interaction	6	12	0.00172
GO:0031347	regulation of defense response	6	12	0.00172
GO:0070596	(1->3)-alpha-glucan biosynthetic process	3	3	0.00195

GO:0000025	maltose catabolic process	3	3	0.00195
GO:0006002	fructose 6-phosphate metabolic process	3	3	0.00195
GO:0006007	glucose catabolic process	3	3	0.00195
GO:0046348	amino sugar catabolic process	3	3	0.00195
GO:0009298	GDP-mannose biosynthetic process	3	3	0.00195
GO:0009056	catabolic process	13	53	0.00222
GO:0006163	purine nucleotide metabolic process	10	30	0.00227
GO:0052173	response to defenses of other organism involved in symbiotic interaction	6	13	0.00286
GO:0009259	ribonucleotide metabolic process	10	31	0.003
GO:1901135	carbohydrate derivative metabolic process	10	37	0.0034
GO:0075136	response to host	6	14	0.0045
GO:0043207	response to external biotic stimulus	6	14	0.0045
GO:0009117	nucleotide metabolic process	6	15	0.00505
GO:0006094	gluconeogenesis	4	7	0.00619
GO:0006073	cellular glucan metabolic process	3	4	0.00627
GO:0008152	metabolic process	67	606	0.00646
GO:0030978	alpha-glucan metabolic process	3	4	0.00709
GO:0046349	amino sugar biosynthetic process	3	4	0.00709
GO:0048584	positive regulation of response to stimulus	7	20	0.00798

Supplementary Table 10 | Sub-module 1 cluster 8, significantly enriched GO terms (biological processes)

GO-ID	Term	Count	Size	p-value
GO:0046903	secretion	2	3	7.31764E-05
GO:0048856	anatomical structure development	3	38	0.00065
GO:0046488	phosphatidylinositol metabolic process	2	8	0.00068
GO:0022402	cell cycle process	3	40	0.00075
GO:0046486	glycerolipid metabolic process	2	13	0.00186
GO:0006644	phospholipid metabolic process	2	19	0.00246
GO:0032505	reproduction of a single-celled organism	2	15	0.00249
GO:0034293	sexual sporulation	2	15	0.00249
GO:1902589	single-organism organelle organization	2	16	0.00284
GO:0048646	anatomical structure formation involved in morphogenesis	2	21	0.00492
GO:0030448	hyphal growth	2	21	0.00492
GO:0006793	phosphorus metabolic process	3	106	0.00586
GO:1902578	single-organism localization	2	27	0.00811
GO:0030154	cell differentiation	2	29	0.00934

Supplementary Table 11 | Sub-module 1 cluster 9, significantly enriched GO terms (biological processes)

GO-ID	Term	Count	Size	p-value
GO:0006544	glycine metabolic process	2	2	0.00026
GO:0044710	single-organism metabolic process	7	236	0.00281
GO:0006563	L-serine metabolic process	2	6	0.00477
GO:0055114	oxidation-reduction process	9	211	0.00715
GO:1901605	alpha-amino acid metabolic process	5	75	0.0096

Supplementary Table 12 | Sub-module 1 cluster 10, significantly enriched GO terms (biological processes)

GO-ID	Term	Count	Size	p-value
GO:0043632	modification-dependent macromolecule catabolic process	20	23	4.774952E-16
GO:0051603	proteolysis involved in cellular protein catabolic process	20	24	2.56519E-15
GO:0009059	macromolecule biosynthetic process	33	68	9.62940E-15
GO:0030163	protein catabolic process	17	21	5.97956E-13
GO:0006518	peptide metabolic process	26	51	9.83876E-12
GO:0043604	amide biosynthetic process	26	54	5.39206E-11
GO:0071840	cellular component organization or biogenesis	38	112	3.80132E-10
GO:0044267	cellular protein metabolic process	24	65	1.95054E-09
GO:0034641	cellular nitrogen compound metabolic process	52	203	9.13732E-09
GO:0051649	establishment of localization in cell	17	34	9.21593E-08
GO:0015833	peptide transport	17	35	1.59928E-07
GO:0006457	protein folding	8	9	3.47631E-07
GO:0070727	cellular macromolecule localization	17	37	4.46409E-07
GO:0034622	cellular macromolecular complex assembly	15	30	5.80739E-07
GO:0070647	protein modification by small protein conjugation or removal	11	17	6.84696E-07
GO:0010468	regulation of gene expression	17	38	7.20769E-07
GO:0006511	ubiquitin-dependent protein catabolic process	7	8	1.79224E-06
GO:0009889	regulation of biosynthetic process	13	26	2.44077E-06
GO:1901566	organonitrogen compound biosynthetic process	31	118	5.67628E-06
GO:0006412	translation	7	10	1.53858E-05
GO:0022613	ribonucleoprotein complex biogenesis	6	7	1.88432E-05
GO:0043161	proteasome-mediated ubiquitin-dependent protein catabolic process	6	7	1.89654E-05
GO:0051246	regulation of protein metabolic process	9	16	2.94784E-05
GO:0051169	nuclear transport	10	19	3.02865E-05
GO:0071826	ribonucleoprotein complex subunit organization	10	19	3.02865E-05
GO:0070585	protein localization to mitochondrion	5	5	3.34880E-05
GO:0001731	formation of translation preinitiation complex	5	5	3.34880E-05
GO:0042026	protein refolding	5	5	3.34880E-05
GO:0006886	intracellular protein transport	12	28	4.58274E-05
GO:0090304	nucleic acid metabolic process	7	11	5.47941E-05
GO:0072594	establishment of protein localization to organelle	9	17	7.43742E-05
GO:0006974	cellular response to DNA damage stimulus	6	8	9.33122E-05
GO:0055085	transmembrane transport	7	11	0.00011
GO:0051171	regulation of nitrogen compound metabolic process	5	6	0.00011
GO:0051179	localization	14	40	0.00013
GO:0022607	cellular component assembly	15	44	0.00017
GO:0070972	protein localization to endoplasmic reticulum	5	6	0.00018
GO:0050658	RNA transport	8	15	0.00018
GO:0006403	RNA localization	8	15	0.00018
GO:0009266	response to temperature stimulus	8	15	0.00018
GO:0015931	nucleobase-containing compound transport	8	15	0.00018
GO:0006950	response to stress	23	86	0.00021
GO:0090150	establishment of protein localization to membrane	4	4	0.00027
GO:0030433	ubiquitin-dependent ERAD pathway	4	4	0.00027
GO:0043254	regulation of protein complex assembly	4	4	0.00027
GO:0006289	nucleotide-excision repair	4	4	0.00027
GO:0006996	organelle organization	10	25	0.00031
GO:0045184	establishment of protein localization	8	17	0.00035
GO:0006413	translational initiation	5	7	0.00039
GO:0044249	cellular biosynthetic process	45	243	0.00067
GO:0051716	cellular response to stimulus	27	116	0.0007

GO:0071705	nitrogen compound transport	9	23	0.00074
GO:0051168	nuclear export	7	14	0.0008
GO:2000112	regulation of cellular macromolecule biosynthetic process	10	28	0.00087
GO:1902582	single-organism intracellular transport	6	11	0.0011
GO:0016567	protein ubiquitination	4	5	0.0011
GO:0034976	response to endoplasmic reticulum stress	4	5	0.0012
GO:1901659	glycosyl compound biosynthetic process	4	5	0.0012
GO:0042455	ribonucleoside biosynthetic process	4	5	0.0012
GO:0071822	protein complex subunit organization	7	15	0.00122
GO:0070271	protein complex biogenesis	7	15	0.00134
GO:0051173	positive regulation of nitrogen compound metabolic process	8	19	0.00136
GO:0002183	cytoplasmic translational initiation	3	3	0.00184
GO:0010558	negative regulation of macromolecule biosynthetic process	3	3	0.00197
GO:0031323	regulation of cellular metabolic process	6	13	0.00205
GO:0044089	positive regulation of cellular component biogenesis	3	3	0.00211
GO:0042128	nitrate assimilation	3	3	0.00211
GO:2001057	reactive nitrogen species metabolic process	3	3	0.00211
GO:0030150	protein import into mitochondrial matrix	3	3	0.00211
GO:0070897	DNA-templated transcriptional preinitiation complex assembly	3	3	0.00211
GO:0045899	positive regulation of RNA polymerase II transcriptional preinitiation complex assembly	3	3	0.00211
GO:0070682	proteasome regulatory particle assembly	3	3	0.00211
GO:2000144	positive regulation of DNA-templated transcription, initiation	3	3	0.00211
GO:0060260	regulation of transcription initiation from RNA polymerase II promoter	3	3	0.00211
GO:1902292	cell cycle DNA replication initiation	3	3	0.00211
GO:0006450	regulation of translational fidelity	3	3	0.00211
GO:0006446	regulation of translational initiation	3	3	0.00211
GO:0046131	pyrimidine ribonucleoside metabolic process	3	3	0.00211
GO:0046134	pyrimidine nucleoside biosynthetic process	3	3	0.00211
GO:0006620	posttranslational protein targeting to endoplasmic reticulum membrane	3	3	0.00211
GO:1902975	mitotic DNA replication initiation	3	3	0.00211
GO:0006997	nucleus organization	3	3	0.00211
GO:0006725	cellular aromatic compound metabolic process	36	183	0.00246
GO:0000280	nuclear division	5	9	0.00273
GO:0010033	response to organic substance	7	17	0.00324
GO:0007264	small GTPase mediated signal transduction	4	6	0.00324
GO:0000070	mitotic sister chromatid segregation	4	6	0.00324
GO:0098813	nuclear chromosome segregation	4	6	0.00324
GO:0009116	nucleoside metabolic process	4	6	0.00324
GO:0071824	protein-DNA complex subunit organization	4	6	0.00324
GO:0046483	heterocycle metabolic process	36	186	0.00336
GO:0044260	cellular macromolecule metabolic process	6	20	0.00384
GO:0051276	chromosome organization	7	18	0.00434
GO:1901293	nucleoside phosphate biosynthetic process	7	18	0.00474
GO:0031325	positive regulation of cellular metabolic process	9	28	0.00602
GO:0044770	cell cycle phase transition	4	7	0.00629
GO:0034654	nucleobase-containing compound biosynthetic process	11	40	0.00654
GO:0017038	protein import	4	7	0.00681
GO:0044238	primary metabolic process	74	473	0.00691
GO:0031327	negative regulation of cellular biosynthetic process	3	4	0.00716
GO:0042787	protein ubiquitination involved in ubiquitin-dependent protein catabolic process	3	4	0.00765
GO:1901988	negative regulation of cell cycle phase transition	3	4	0.00765
GO:0051054	positive regulation of DNA metabolic process	3	4	0.00765
GO:0031146	SCF-dependent proteasomal ubiquitin-dependent protein	3	4	0.00765

	catabolic process			
GO:0010564	regulation of cell cycle process	6	15	0.00768
GO:0036211	protein modification process	13	50	0.0077
GO:0009156	ribonucleoside monophosphate biosynthetic process	5	11	0.00806
GO:0016070	RNA metabolic process	11	42	0.00972
GO:1903047	mitotic cell cycle process	5	12	0.00993

Supplementary Table 13 | Sub-module 1 cluster 11, significantly enriched GO terms (biological processes)

GO-ID	Term	Count	Size	p-value
GO:0000375	RNA splicing, via transesterification reactions	6	7	2.70567E-09
GO:0045292	mRNA cis splicing, via spliceosome	5	5	1.61354E-08
GO:0016070	RNA metabolic process	9	68	7.10973E-05
GO:0010467	gene expression	10	91	0.00013

Supplementary Table 14 | Sub-module 1 cluster 12, significantly enriched GO terms (biological processes)

GO-ID	Term	Count	Size	p-value
GO:0006508	proteolysis	5	33	4.74361E-08
GO:1902086	fumagillin biosynthetic process	2	4	0.00015
GO:0097176	epoxide metabolic process	2	4	0.00015
GO:0043170	macromolecule metabolic process	5	176	0.00027

Supplementary Table 15 | Sub-module 1 cluster 13, significantly enriched GO terms (biological processes)

GO-ID	Term	Count	Size	p-value
GO:1900815	monodictyphenone biosynthetic process	13	13	5.33344E-10
GO:0042180	cellular ketone metabolic process	16	21	2.70294E-08
GO:0042537	benzene-containing compound metabolic process	13	15	3.82228E-08
GO:0050761	depsipeptide metabolic process	9	9	4.13701E-07
GO:1900557	emericellamide biosynthetic process	9	9	4.13701E-07
GO:1901334	lactone metabolic process	9	9	4.13701E-07
GO:1900584	o-orsellinic acid biosynthetic process	8	8	2.15750E-06
GO:0018958	phenol-containing compound metabolic process	12	17	6.10997E-06
GO:0019748	secondary metabolic process	17	34	2.86743E-05
GO:0045461	sterigmatocystin biosynthetic process	13	22	4.76086E-05
GO:0009404	toxin metabolic process	13	22	4.76086E-05
GO:1901376	organic heteropentacyclic compound metabolic process	14	25	5.44246E-05
GO:2001307	xanthone-containing compound biosynthetic process	5	5	0.0003
GO:1900554	asperfuranone biosynthetic process	5	5	0.0003
GO:1902644	tertiary alcohol metabolic process	5	5	0.0003
GO:0019184	nonribosomal peptide biosynthetic process	7	10	0.00077
GO:1901617	organic hydroxy compound biosynthetic process	18	45	0.00116
GO:0030638	polyketide metabolic process	5	6	0.0015
GO:0034311	diol metabolic process	5	6	0.0015
GO:0034440	lipid oxidation	4	4	0.00152
GO:0006635	fatty acid beta-oxidation	4	4	0.00152
GO:0008152	metabolic process	162	748	0.00172
GO:0006629	lipid metabolic process	28	90	0.00518
GO:0036184	asperthecin biosynthetic process	3	3	0.00776
GO:1900793	shamixanthone biosynthetic process	3	3	0.00776
GO:1900587	arugosin biosynthetic process	3	3	0.00776
GO:1900575	emodin biosynthetic process	3	3	0.00776
GO:0046173	polyol biosynthetic process	5	8	0.00983

Supplementary Table 16 | Sub-module 1 cluster 14, significantly enriched GO terms (biological processes)

GO-ID	Term	Count	Size	p-value
GO:0006012	galactose metabolic process	2	5	0.00051
GO:0009100	glycoprotein metabolic process	2	5	0.00051
GO:0070085	glycosylation	2	5	0.00051
GO:0036211	protein modification process	3	50	0.00475

Supplementary Table 17 | Sub-module 1 cluster 15, significantly enriched GO terms (biological processes)

GO-ID	Term	Count	Size	p-value
GO:0046034	ATP metabolic process	5	21	2.17781E-07
GO:0009199	ribonucleoside triphosphate metabolic process	5	21	2.17781E-07
GO:0009144	purine nucleoside triphosphate metabolic process	5	21	2.17781E-07
GO:0009126	purine nucleoside monophosphate metabolic process	5	27	8.49463E-07
GO:0009161	ribonucleoside monophosphate metabolic process	5	29	1.24250E-06
GO:0006163	purine nucleotide metabolic process	5	30	1.48677E-06
GO:0009259	ribonucleotide metabolic process	5	31	1.76766E-06
GO:0042773	ATP synthesis coupled electron transport	3	6	8.98325E-06
GO:0006753	nucleoside phosphate metabolic process	5	47	1.52431E-05
GO:0022900	electron transport chain	3	7	1.56552E-05
GO:0006123	mitochondrial electron transport, cytochrome c to oxygen	2	2	6.84496E-05
GO:1901135	carbohydrate derivative metabolic process	5	65	7.78922E-05
GO:0009142	nucleoside triphosphate biosynthetic process	2	3	0.0002
GO:0009206	purine ribonucleoside triphosphate biosynthetic process	2	3	0.0002
GO:0015986	ATP synthesis coupled proton transport	2	3	0.0002
GO:0009060	aerobic respiration	3	18	0.00035
GO:0098660	inorganic ion transmembrane transport	2	4	0.00041
GO:0098655	cation transmembrane transport	2	4	0.00041
GO:1902600	hydrogen ion transmembrane transport	2	4	0.00041
GO:0006818	hydrogen transport	2	5	0.00068
GO:0015672	monovalent inorganic cation transport	2	5	0.00068
GO:0006793	phosphorus metabolic process	5	113	0.00115
GO:0009168	purine ribonucleoside monophosphate biosynthetic process	2	9	0.00239
GO:0043933	macromolecular complex subunit organization	3	35	0.0026
GO:0006811	ion transport	2	10	0.00297
GO:0006139	nucleobase-containing compound metabolic process	5	139	0.00303
GO:0055085	transmembrane transport	2	11	0.00362
GO:0009152	purine ribonucleotide biosynthetic process	2	11	0.00362
GO:0009124	nucleoside monophosphate biosynthetic process	2	11	0.00362
GO:0016310	phosphorylation	3	41	0.00413
GO:0006461	protein complex assembly	2	14	0.00448
GO:0022607	cellular component assembly	3	44	0.00507
GO:0046390	ribose phosphate biosynthetic process	2	14	0.00591
GO:0072522	purine-containing compound biosynthetic process	2	16	0.00772
GO:0009165	nucleotide biosynthetic process	2	18	0.00975

Supplementary Table 18 | Sub-module 2, significantly enriched GO terms (biological processes)

GO-ID	Term	Count	Size	p-value
GO:0044275	cellular carbohydrate catabolic process	3	21	0.00208

Supplementary Table 19 | Sub-module 3, significantly enriched GO terms (biological processes)

GO-ID	Term	Count	Size	p-value
GO:0009395	phospholipid catabolic process	4	5	8.85073E-08
GO:0016042	lipid catabolic process	5	18	1.24586E-06
GO:0046503	glycerolipid catabolic process	3	4	7.09360E-06
GO:0042173	regulation of sporulation resulting in formation of a cellular spore	2	2	0.00016
GO:1901568	fatty acid derivative metabolic process	2	2	0.00016
GO:0001516	prostaglandin biosynthetic process	2	2	0.00016
GO:0046456	icosanoid biosynthetic process	2	2	0.00016
GO:0034306	regulation of sexual sporulation	2	2	0.00016
GO:0006692	prostanoid metabolic process	2	2	0.00016
GO:0031408	oxylipin biosynthetic process	2	2	0.00016
GO:0043941	positive regulation of sexual sporulation resulting in formation of a cellular spore	2	2	0.00016
GO:0043942	negative regulation of sexual sporulation resulting in formation of a cellular spore	2	2	0.00016
GO:0043944	negative regulation of asexual sporulation resulting in formation of a cellular spore	2	2	0.00016
GO:0043945	positive regulation of asexual sporulation resulting in formation of a cellular spore	2	2	0.00016
GO:0006650	glycerophospholipid metabolic process	3	12	0.00037
GO:0045596	negative regulation of cell differentiation	2	3	0.00048
GO:0045597	positive regulation of cell differentiation	2	3	0.00048
GO:0072338	cellular lactam metabolic process	2	3	0.00048
GO:0016999	antibiotic metabolic process	2	3	0.00048
GO:0006636	unsaturated fatty acid biosynthetic process	2	3	0.00048
GO:0043938	positive regulation of sporulation	2	3	0.00048
GO:0043939	negative regulation of sporulation	2	3	0.00048
GO:0042316	penicillin metabolic process	2	3	0.00048
GO:0006629	lipid metabolic process	2	8	0.00049
GO:0010913	regulation of sterigmatocystin biosynthetic process	2	4	0.00095
GO:1903046	meiotic cell cycle process	3	17	0.00109
GO:0034305	regulation of asexual sporulation	2	5	0.00158
GO:0006793	phosphorus metabolic process	6	113	0.00164
GO:0048646	anatomical structure formation involved in morphogenesis	3	21	0.00207
GO:0044702	single organism reproductive process	3	21	0.00207
GO:0019954	asexual reproduction	3	23	0.0021
GO:0006071	glycerol metabolic process	2	7	0.00327
GO:0043455	regulation of secondary metabolic process	2	7	0.00327
GO:0045787	positive regulation of cell cycle	2	8	0.00432
GO:0003006	developmental process involved in reproduction	3	27	0.00436
GO:0006979	response to oxidative stress	3	28	0.00485
GO:0044699	single-organism process	9	492	0.00496
GO:0050793	regulation of developmental process	2	9	0.00551
GO:2000243	positive regulation of reproductive process	2	9	0.00551
GO:2000242	negative regulation of reproductive process	2	10	0.00684
GO:0019953	sexual reproduction	3	34	0.00846

Supplementary Table 20 | Sub-module 4, significantly enriched GO terms (biological processes)

GO-ID	Term	Count	Size	p-value
GO:0034660	ncRNA metabolic process	16	23	2.42560E-24
GO:0006396	RNA processing	18	35	8.24804E-22
GO:0090304	nucleic acid metabolic process	22	79	1.50567E-20
GO:0044085	cellular component biogenesis	21	73	1.19110E-19
GO:0006364	rRNA processing	11	18	6.93887E-16
GO:0044260	cellular macromolecule metabolic process	16	80	1.99584E-13
GO:0042274	ribosomal small subunit biogenesis	10	15	2.40306E-13
GO:0006725	cellular aromatic compound metabolic process	22	183	5.73612E-12
GO:0046483	heterocycle metabolic process	22	186	8.22252E-12
GO:0034641	cellular nitrogen compound metabolic process	22	203	5.6265E-11
GO:0090501	RNA phosphodiester bond hydrolysis	6	6	3.94421E-10
GO:0000478	endonucleolytic cleavage involved in rRNA processing	6	6	3.94421E-10
GO:1901360	organic cyclic compound metabolic process	22	224	4.76599E-10
GO:0000447	endonucleolytic cleavage in ITS1 to separate SSU-rRNA from 5.8S rRNA and LSU-rRNA from tricistronic rRNA transcript (SSU-rRNA, 5.8S rRNA, LSU-rRNA)	5	5	1.61354E-08
GO:0071826	ribonucleoprotein complex subunit organization	7	19	3.66270E-07
GO:0042273	ribosomal large subunit biogenesis	4	5	1.60276E-06
GO:0000027	ribosomal large subunit assembly	4	5	3.09590E-06
GO:0070925	organelle assembly	5	12	1.10630E-05
GO:0034622	cellular macromolecular complex assembly	7	30	1.18883E-05
GO:0000463	maturation of LSU-rRNA from tricistronic rRNA transcript (SSU-rRNA, 5.8S rRNA, LSU-rRNA)	3	3	2.37560E-05
GO:1902626	assembly of large subunit precursor of preribosome	3	3	2.37560E-05
GO:0097659	nucleic acid-templated transcription	6	30	0.00014
GO:0071166	ribonucleoprotein complex localization	4	12	0.00027
GO:0006405	RNA export from nucleus	4	13	0.00038
GO:0006611	protein export from nucleus	4	13	0.00038
GO:0000462	maturation of SSU-rRNA from tricistronic rRNA transcript (SSU-rRNA, 5.8S rRNA, LSU-rRNA)	3	7	0.00042
GO:0033753	establishment of ribosome localization	3	6	0.00045
GO:0071428	rRNA-containing ribonucleoprotein complex export from nucleus	3	6	0.00045
GO:0000466	maturation of 5.8S rRNA from tricistronic rRNA transcript (SSU-rRNA, 5.8S rRNA, LSU-rRNA)	2	2	0.00057
GO:0050657	nucleic acid transport	4	15	0.00069
GO:0051236	establishment of RNA localization	4	15	0.00069
GO:0098781	ncRNA transcription	2	2	0.00074
GO:0051640	organelle localization	3	7	0.00077
GO:0000154	rRNA modification	2	2	0.0008
GO:0042797	tRNA transcription from RNA polymerase III promoter	2	2	0.00086
GO:0000055	ribosomal large subunit export from nucleus	2	2	0.00086
GO:0000472	endonucleolytic cleavage to generate mature 5'-end of SSU-rRNA from (SSU-rRNA, 5.8S rRNA, LSU-rRNA)	2	2	0.00086
GO:0001510	RNA methylation	2	2	0.00086
GO:0034471	ncRNA 5'-end processing	2	2	0.00086
GO:0010467	gene expression	3	23	0.00093
GO:0016043	cellular component organization	8	81	0.00097
GO:0044238	primary metabolic process	22	473	0.00142
GO:0006913	nucleocytoplasmic transport	4	19	0.0018
GO:0006402	mRNA catabolic process	2	3	0.00253
GO:0010605	negative regulation of macromolecule metabolic process	4	22	0.00319
GO:0006360	transcription from RNA polymerase I promoter	2	4	0.00461
GO:0034654	nucleobase-containing compound biosynthetic process	6	58	0.00550

Supplementary Table 21 | Sub-module 5, significantly enriched GO terms (biological processes)

GO-ID	Term	Count	Size	p-value
GO:0006810	transport	7	60	4.10111E-07
GO:0051179	localization	7	67	9.03711E-07
